# TFPI1 Mediates Resistance to Doxorubicin in Breast Cancer Cells by Inducing a Hypoxic-Like Response

**DOI:** 10.1371/journal.pone.0084611

**Published:** 2014-01-28

**Authors:** Gerald F. Davies, Arnie Berg, Spike D. L. Postnikoff, Heather L. Wilson, Terra G. Arnason, Anthony Kusalik, Troy A. A. Harkness

**Affiliations:** 1 Department of Anatomy and Cell Biology, University of Saskatchewan, Saskatoon, Saskatchewan, Canada; 2 Department of Medicine, University of Saskatchewan, Saskatoon, Saskatchewan, Canada; 3 Department of Computer Science, University of Saskatchewan, Saskatoon, Saskatchewan, Canada; 4 Vaccine and Infectious Disease Organization (VIDO), University of Saskatchewan, Saskatoon, Saskatchewan, Canada; Ospedale Pediatrico Bambino Gesu', Italy

## Abstract

Thrombin and hypoxia are important players in breast cancer progression. Breast cancers often develop drug resistance, but mechanisms linking thrombin and hypoxia to drug resistance remain unresolved. Our studies using Doxorubicin (DOX) resistant MCF7 breast cancer cells reveals a mechanism linking DOX exposure with hypoxic induction of DOX resistance. Global expression changes between parental and DOX resistant MCF7 cells were examined. Westerns, Northerns and immunocytochemistry were used to validate drug resistance and differentially expressed genes. A cluster of genes involved in the anticoagulation pathway, with Tissue Factor Pathway Inhibitor 1 (TFPI1) the top hit, was identified. Plasmids overexpressing TFPI1 were utilized, and 1% O_2_ was used to test the effects of hypoxia on drug resistance. Lastly, microarray datasets from patients with drug resistant breast tumors were interrogated for TFPI1 expression levels. TFPI1 protein levels were found elevated in 3 additional DOX resistant cells lines, from humans and rats, indicating evolutionarily conservation of the effect. Elevated TFPI1 in DOX resistant cells was active, as thrombin protein levels were coincidentally low. We observed elevated HIF1α protein in DOX resistant cells, and in cells with forced expression of TFPI1, suggesting TFPI1 induces HIF1α. TFPI1 also induced c-MYC, c-SRC, and HDAC2 protein, as well as DOX resistance in parental cells. Growth of cells in 1% O_2_ induced elevated HIF1α, BCRP and MDR-1 protein, and these cells were resistant to DOX. Our *in vitro* results were consistent with *in vivo* patient datasets, as tumors harboring increased BCRP and MDR-1 expression also had increased TFPI1 expression. Our observations are clinically relevant indicating that DOX treatment induces an anticoagulation cascade, leading to inhibition of thrombin and the expression of HIF1α. This in turn activates a pathway leading to drug resistance.

## Introduction

The development of drug resistant cancer is a major challenge impeding cancer therapy. Although many molecular mechanisms are known to cause drug resistance, very little is known regarding how to terminally impair the growth of these cells [1]. In order to understand the cellular changes involved in the development of drug resistance, we analyzed the temporal changes in gene signatures in breast cancer cells as they were experimentally induced and selected to become resistant to the chemotherapy drug Doxorubicin (DOX). Among a variety of changes induced as cells progressed towards DOX resistance, a number of upregulated anticoagulant genes were of particular interest. It is well established that cancer is associated with coagulation alterations, with increased coagulation through tissue factor (TF) and thrombin expression increasing angiogenesis, metastasis and tumor invasiveness [2], [3]. Hypercoagulation in the peripheral circulation, due to activation of platelets and/or tumoral release of procoagulant molecules, has been identified in specific malignancies [4], [5]. Furthermore, a recent study also supported a role for the coagulation pathway in cancer development, as tumor-derived TF protein, expressed within the tumor microenvironment but not by unaffected surrounding cells, is important for cancer progression [6]. Therefore, expression of anticoagulant proteins should at least act as tumor suppressors. This appears to be the case for Tissue Factor Pathway Inhibitor 2 (TFPI2), as methylated, and silenced TFPI2 DNA is used as a biomarker for metastatic cancer [7]. Interestingly, while TFPI2 mRNA was not altered in our study, the related TFPI1 mRNA was.

Through a cascade of interacting factors, TF leads to the generation of thrombin [8]. Once thrombin is produced, fibrinogen is cleaved to fibrin, activating platelets in the coagulation-dependent pathway. Importantly, thrombin also cleaves and turns on protease-activated receptors leading to transcription of angiogenic factors promoting new blood vessel formation. Thus, inhibiting the TF/thrombin pathway has been an attractive target for anticancer therapy to limit new blood supply. For example, the anticoagulant Heparin has been used to prevent cancer-associated venous thromboembolism, yet appears to also antagonize cancer metastasis [9], [10]. However, it has recently been reported that the benefit provided by anticoagulation drugs on cancer development is short-lived and can eventually induce “evasive resistance” via hypoxic induction of cancer stem cells [11], [12]. The TF/thrombin coagulation pathway clearly plays a complex role in cancer metastasis, which requires further clarification.

TFPI2 appears to be a perfect example of how an endogenous thrombin inhibitor can serve to normally keep the coagulation cascade in check, ultimately fulfilling a tumor suppressor role [13], [14]. TFPI1, however, has been found elevated in many aggressive cancers [15]–[19]. It has been proposed that fibrin, generated as a result of thrombin activity, may provide a natural defense mechanism against tumor metastasis [20]. One outcome of TFPI1 induction is decreased fibrin levels, potentially offering cancer cells a means to bypass the protective effect afforded by fibrin [15]. TFPI1, like TFPI2, is a serine protease inhibitor that prevents TF/factor VIIa activation of factor X, thereby blocking the generation of thrombin and fibrin, which is primarily the reason why the endothelium provides an antithrombotic interface with circulating blood. TFPI1 is alternatively spliced and capable of producing three isoforms in humans termed TFPI1α, TFPI1β and TFPI1δ [13]. TFPI1α is the major isoform in human endothelium, being approximately 10-fold more abundant compared to TFPI1β [21]. Interestingly, TFPI1α overexpression *in vitro* resulted in elevated mRNA levels of many genes tightly associated with cancer, including genes encoding immune and inflammatory factors [22]. It is clearly of interest to determine the precise role that TFPI1 plays in cancer.

Although both TFPI1 and TFPI2 are thrombin inhibitors, they have different functions, which may reflect their different cellular locations. The majority of TFPI2 is released and found in the extracellular matrix [23]. On the other hand, the majority of TFPI1 associates with the endothelial cell surface via a glycosyl-phosphatidylinositol (GPI) anchor [15], [19]. Studies in melanoma tumors highlight the different functions of TFPI1 and 2, both of which are overexpressed in addition to TF. The anticoagulant and endothelial-like nature of highly aggressive melanoma tumors is controlled by TF and TFPI1, but not TFPI2; TFPI1 associates with the tumor cell surface and inhibits TF, while TFPI2 is secreted by the tumor cell into the extracellular matrix and assists in plasticity of the vascular phenotype [24]. The anticoagulant activity of TFPI1 on the cell surface, and the subsequent inhibition of TF, may play important roles in tumor progression.

The concept that TFPI1 imparts increased tumorigenic potential is controversial, as literature also exists suggesting TFPI1, like TFPI2, is a tumor suppressor [25]–[27]. Recent studies support a suppressive role, as increased TFPI1 protein is associated with apoptosis and inhibits cell line invasiveness, whereas TFPI1 silencing increased metastatic growth [28]–[30]. One can envision arguments for either case. On the one hand, elevated TFPI1 expression may be an attempt by the cell to combat the tumorigenic potential of increased thrombin levels. On the other hand, increased TFPI1 at the tumor site may reduce thrombin, leading to hypoxia and the subsequent expression of HIF1α, a potent driver of angiogenesis and invasive cancer. Our *in vitro* and *in vivo* results support the hypothesis that TFPI1 is tightly linked with the development of drug resistance by generating a hypoxic-like environment, leading to the induction of HIF1α and the onset of drug resistance.

## Methods

### Cell lines, reagents and permits

All cell lines used in this study were purchased from the American Type Culture Collection (ATCC) in Manassas, VA, USA. DMEM-F12 media was purchased from Sigma Aldrich Canada. Fetal bovine serum and antibiotics were purchased from Invitrogen. Molecular biology grade skim milk powder was purchased from BioRad Laboratories Canada (Mississauga, ON) while DOX and all other reagents were purchased from Sigma Aldrich Canada. All necessary permits were obtained for the described study, which complied with all relevant regulations.

### Selection of Doxorubicin resistant cells

MCF7 cells were selected for Doxorubicin (DOX) resistance based on our previous published methods [31]. Specific applications of our methods are explained as follows. MCF7 cells were cultured in the presence of 1 µM DOX for 48 hours. At the end of this period the cells were washed twice with sterile phosphate-buffered saline (PBS) to remove the DOX and fresh DOX-free growth medium was added. Following a 3-day recovery period 100 nM DOX was added to the cells for 2 weeks with washes and media changes every 3 days. At the end of the 2-week selection period the cells were subjected to MDR-1 and BCRP Western and ICC analysis to verify DOX resistance, presumably a multiple drug resistant (MDR) state, as previously induced in K562 leukemia cells [32]. Chemoresistant F98 rat glioblastoma multiforme cells and control C6 glioma cells were purchased from ATCC. Human Colo 201 colorectal cancer cells were cultured in the presence of 1 µM DOX for 72 hours. Cell cultures were washed 3 times following the induction period and were allowed to recover for 3 days in normal media. Following this recovery period selection pressure was reinstituted with 50 nM DOX for a period of 2 weeks with media and DOX changes every 3 days. Following the 2-week selection period resistant Colo201 cells were tested by MDR-1 and BCRP Western analysis to verify MDR induction. All MDR cell lines used in this study were free of DOX selection prior to experimentation.

### Standard methods

Immunocytochemistry (ICC) was performed using MCF7 and MCF7/DOX cells. The cells were fixed in 4% paraformaldehyde in PBS and permeabilized using 0.1% Triton X-100. Samples were then blocked using 10% serum for 2 hours at 25°C, then incubated with the primary antibodies at a 1/50 dilution for 12 hours at 4°C. A rabbit anti-mouse polyclonal conjugated to Alexa Fluor 594 was used as the secondary at a 1/50 dilution. ELISAs were performed as follows. Thrombin-Antithrombin Complex (TAT) concentrations in the supernatants from MCF7 parent and selected cells were determined using the TAT complex ELISA Kit as indicated by the manufacturer (USCN Life Sciences, Inc. ABIN365773). Western and Northern analyses, MTT cell viability assays, RNA silencing, and RNA extraction were described previously [31]–[33]. Antibodies against TFPI1, PAR-1, thrombin, H2AX^phos^, c-MYC, c-SRC, HDAC1 and HDAC2 were obtained from Santa Cruz Biotechnology (Santa Cruz, CA). Antibodies against GAPDH, MDR-1, actin and tubulin were purchased from Sigma-Aldrich (St Louis, MI). Antibodies against BCRP, GFP and H2B^tot^ were obtained from Abcam (Cambridge, MA). Antibodies against HIF1α (Enzo Life Sciences; Brockville, Ontario), SerpinA5 (Assay Biotech; Sunnyvale, CA), H3K9/14^Ac^ and H3K14^Ac^ (Cell Signaling; Danvers, MA), H3^tot^ (Millipore; Billerica, MA), and secondary HRP antibodies (Bio-Rad Laboratories; Hercules, CA) were purchased from the suppliers indicated. For the Northern analysis, a TOPO/TFPI plasmid was obtained from Dr. Nina Iverson (University of Oslo), with a 3,000 bp TFPI1 cDNA fragment released by restriction endonuclease digestion. For TFPI1 RNA silencing, siRNA duplex solutions (fluorescein-conjugated, scrambled siRNA control and TFPI1 siRNAs) were prepared by adding 50 nM of TFPI1 siRNA to the transfection reagent LipofectamineRNAiMax (Invitrogen). Hypoxia experiments were performed as previously described [34], using a modular hypoxia chamber (Billups–Rothenberg, Del Mar, CA, USA).

### Microarray hybridization

Total RNA was shipped on dry ice and sent to the Laboratory for Advanced Genome Analysis at the Vancouver Prostate Centre for microarray analysis (http://www.mafpc.ca/). Total RNA was used as a template to create labeled cDNA using MessageAmp™ Premier RNA Amplification Kit and MessageAmp™ III RNA Amplification Kit (Applied Biosystems) according to the manufacturer's instructions. Labeled cDNA was hybridized on Illumina HumanHT-12 BeadChip Microarrays, which are comprised of more than 25,000 annotated genes. Scanning and data acquisition were obtained using the Illumina iScan scanner, raw data (idat files) were loaded into Illumina BeadStudio, without background subtraction, and exported for analysis. The data files have been deposited with ArrayExpress (accession # E-MTAB-1643).

### Data mining

The tab-delimited text files as exported by the Illumina BeadStudio package were imported into FlexArray [35]. In addition to the data file, a control file containing 783 control features was also imported as provided by Illumina BeadStudio. The TargetID of each probe was supplemented with annotation information provided by Illumina. Each imported sample consisted of raw signal, bead count, bead standard deviation, and detection p-value. The experimental design analyzed three samples of MCF7 control arrays, two of MCF7 treated with DOX for 48 hours, and three of DOX selected samples. All samples were filtered prior to analysis using the default FlexArray standard detection p-value of .05 and proportion per group threshold of .5 (i.e. probes must be “detected” in at least half the samples in the group). This reduced the count of reported probes from 48,794 to 16,079. No background normalization was done on the raw data.

The DOX 48 hr treatment of the filtered data was then contrasted to the MCF7 cell samples, and the DOX selected samples were contrasted to the DOX 48 hr samples. The *lumi* algorithm [36] was applied to the samples, using the Variance Stabilization Transformation (VST) on the individual microarrays to take advantage of the normalization available by virtue of the multiple bead count information on the arrays [37]. In addition to the VST normalization within arrays, the Loess normalization method was applied to normalize between arrays. The resulting data was a normalized expression value for each gene on each array.

**Figure 1:**
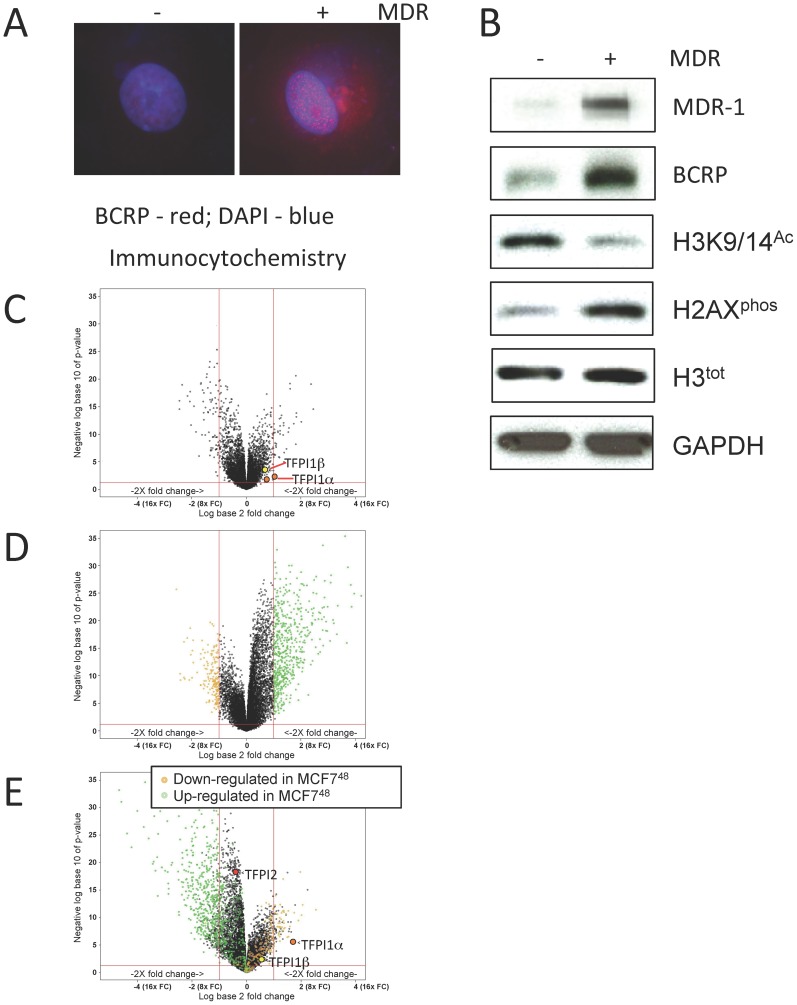
DOX resistant MCF7 breast cancer cells are associated with chromatin alterations and DNA damage. (**A**) MCF7 cells before and after DOX selection were stained with DAPI to visualize DNA (blue) and with antibodies against BCRP (red). (**B**) Protein lysates were prepared from parental and selected MCF7 cells and analyzed by Western analyses using the antibodies shown. (**C**) Volcano plot of genes differentially expressed following the full DOX selection protocol compared to starting parental cells. The X-axis denotes expression changes with positive to the right and negative to the left. The Y-axis shows statistical significance (p-value) of the changes observed. The vertical red lines define the threshold for 2-fold positive and negative changes. (**D**) Volcano plot of differentially expressed genes following acute exposure to 1 µM DOX for 48 hours. The dots in green and yellow define the genes that were up-regulated and down-regulated, respectively. (**E**) Volcano plot demonstrating the differential expression of genes following the 2-week chronic exposure phase after the 48-hour treatment. The Tissue Factor Pathway Inhibitor family members are shown (TFPI1α, TFPI1β and TFPI2).

To identify differentially expressed genes, a two-sample Bayesian *t*-test was run for each contrast generated by the *lumi* algorithm. This test determines the mean and standard deviation of the samples in each treatment and calculates the fold change and p-value for each gene of each contrasting treatment. Applying fold change thresholds of 2.0 and −2.0, and a maximum p-value of .05 produced volcano plots illustrating first the up-regulation of a large proportion of expressed genes in the MCF7 cells treated with DOX for 48 hr, then the down-regulation of an equally large proportion of expressed genes in the DOX 48 hr selected cells sampled after a two week period. To assess the overlap between the large proportion of genes up and down-regulated in the two treatments, those genes down-regulated in the volcano plot of the first treatment corresponding to genes up-regulated in the second treatment, and vice versa, were marked in yellow and green, respectively, in Figs. 1D and 1E.

### *In vivo* interrogation of human cancer microarray datasets

In order to compare our findings to *in vivo* gene expression, tumor datasets for three cancers (breast, colon and ovarian) were downloaded from The Cancer Genome Atlas (TCGA; https://tcga-data.nci.nih.gov/tcga/). Molecular tumor gene expression data are provided per patient sample as gathered from Agilent microarray gene expression assays. We analyzed data from 529 breast cancer patients, 539 ovarian cancer patients and 155 colon cancer patients.

In the data sets, expression values were obtained using differential hybridization of cancer RNA and Universal Reference RNA (URR) cell lines. URR is high-quality total RNA from ten cell lines for human microarray gene-expression profiling that acts as a consistent control for standard data set comparisons, giving broad gene coverage. URR is hybridized on the microarray along with the patient sample RNA. The expression data is also subjected to lowess normalization and pooling steps as set out and described by TCGA.

The downloaded data, consisting of expression levels for genes per patient sample from combined normalized probes, were processed to extract the data for the genes of interest. The dataset for each type of cancer was divided into two subsets, depending on the drug-resistant state of the patient's tumor. That state was inferred from the differential expression of BCRP (ABCG2). In other words, data points for all genes were collected for those patients where BCRP was observed to have elevated expression, and also for those patients where BCRP was observed to have reduced expression. In total, 4 breast, 2 ovarian, and 13 colon tumors expressed BCRP at elevated levels compared to URR samples. We combined the 19 samples where BCRP was up (BCRP Up) into one pooled set, and the 1204 samples where BCRP was not elevated (BCRP Down) into another pooled set.

The genes of primary concern for comparison purposes were thrombin (F2), HIF1α, TFPI1 (TFPI) and TFPI2. From our *in vitro* analyses, thrombin and TFPI2 should be low and relatively unchanged in expression, whereas HIF1α,and TFPI1 should be elevated in drug resistant cells. To act as controls, a set of housekeeping genes, not part of our differentially expressed gene lists, were selected as previously described [38]. These genes included actin (ACTB), tubulin (TUBA1B), RPL27, and RPL22. Additionally, two genes that showed reductions in expression in MCF7 drug-resistant cells, MT2A and MUC1, were selected. MDR-1 (ABCB1) was also included as a marker to confirm drug-resistance.

Using a collection of software components, including an R script, the gene expression data for each selected gene for each cancer type were plotted in boxplots. Each boxplot illustrates the gene expression for the set of data where BCRP expression was elevated, compared to the set of data where BCRP expression was reduced. The left and right boundaries of each box define the ends of the first (25 percentile) and third (75 percentile) quartiles, respectively, around the median. The left whisker represents the 25th percentile minus the product of 1.5 times IQR (the InterQuartile Range or 50% of data). The right whisker represents the 75th percentile plus the product of 1.5 times IQR. Any data points outside the whisker range are reported as outliers. Statistical significance is shown using notched boxplots. The ends of the notches mark plus or minus 1.58 times the interquartile range divided by the square root of n about the median, where n is the number of sample points. If the notches of two boxplots do not overlap, we can conclude that the medians are different with at least 95% confidence.

## Results

### Chronic DOX exposure induces gene expression changes coincident with drug resistance

For this study, we followed our established protocol for selection and propagation of DOX resistant cancer cell lines that incorporates the use of an acute (48 hour) pulse of high dose DOX (1 µM) to initiate drug resistance followed by a chronic (2 week) low dose DOX (100 nM) selection to establish drug resistance in surviving cells. The DOX resistance status of these cells was confirmed by increased expression of the drug transporters MDR-1 and BCRP (Figs. 1A and 1B) [31], [32]. A further potential marker of DOX resistance is decreased histone acetylation, a hallmark of aggressive cancer [39]. Here we show that histone H3 acetylation was reduced in DOX selected cells, while histone H2AX phosphorylation (H2AX^phos^), a measure of DNA damage [40], increased (Fig. 1B). Thus, MCF7 DOX selected cells exhibit signs of drug resistance.

We were interested in determining what gene expression changes were associated with MCF7 cells following the acute and chronic selection phases. Therefore, samples were prepared for microarray analyses from (i) parental MCF7 breast cancer cells before, and (ii) after acute DOX exposure (DOX^48^), and (iii) after chronic DOX exposure (DOX^Sel^). A volcano plot demonstrates that many genes were differentially expressed, both up and down, when comparing DOX^Sel^ with parental cells (Fig. 1C; Table S1; 73 genes down-regulated and 47 up-regulated). To identify those genes specifically associated with the establishment of DOX resistance, we compared genes differentially expressed after the 48 hour acute phase with those following the chronic phase, and found that many genes differentially expressed in DOX^48^ cells returned to their baseline expression in DOX^Sel^ cells (compare the volcano plots in Figs. 1D and 1E; Table S2). The genes that returned to their baseline state are listed in Table S3. Therefore, those genes that revert reflect an acute and transient response to high dose DOX exposure and are therefore not considered integral to DOX selection.

**Figure 2:**
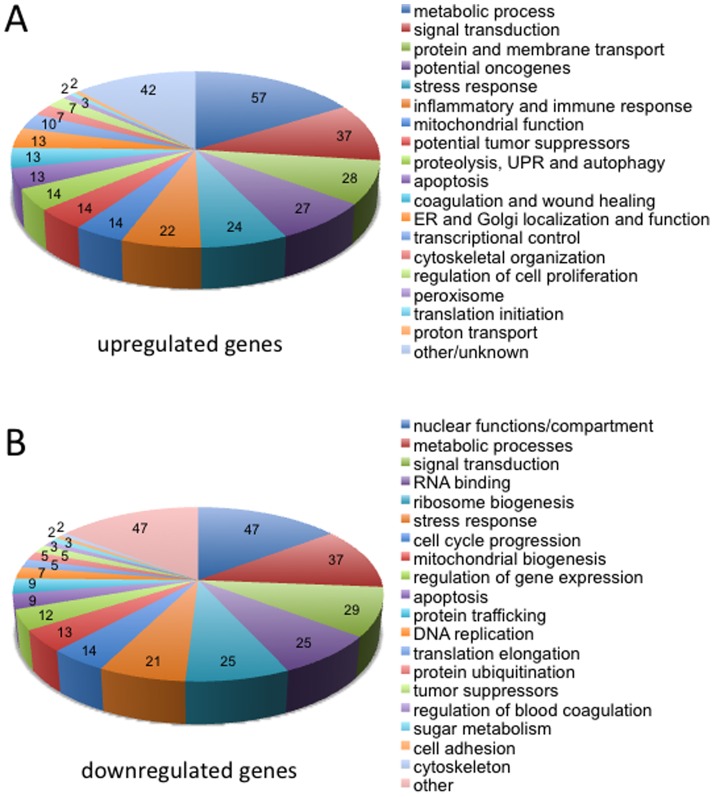
Gene ontology list of differentially altered functions following selection of DOX resistant MCF7 cells. (**A**) Cellular functions involving up-regulated genes. (**B**) Cellular functions affected by the down-regulated genes. The number of genes in each category is shown.

We focused on those gene signatures that remained, or became, differentially expressed specifically during chronic exposure to DOX (Figure S1, Table S4). In total, 207 genes were up-regulated (154 acute, 53 chronic) and 220 down-regulated (118 acute, 102 chronic). The complete lists of up and down-regulated processes involved in DOX resistance are shown in Tables 1 and 2, and Tables S5, S6, S7, S8. The pie charts presented in Fig. 2 demonstrate the full range of functions impacted by DOX selection (also see Figs. S2 and S3). A Venn diagram was used to show the overlap of the categories involved in metabolic processes, which encompassed the largest proportion of genes affected both up and down (94 out of 410 genes; 22.9%; Fig. S4). Protein and steroid metabolic functions were found to contain both up- and down-regulated genes (Table S9). Overall, the differentially expressed genes strongly support a transcriptional program involved in the establishment of aggressive cancer.

**Table 1:** Combined up-regulated processes during DOX selection.

Category	Count	Genes
metabolic process	57	ALDH4A1, RPS27L, FBXO22, PINK1, PEPD, SELS, RPN1, RPN2, ACADVL, BMP1, GADD45G, GADD45A, ECH1, POLR2L, SQSTM1, GNS, GABARAPL2, PIGT, ISG20, DNAJB2, AGPAT2, G6PD, IRAK1, UBE2F, RNASET2, REXO2, DPM3, DDX24, TSC22D1, PSAP, MRPL41, RRBP1, PSMB4, PSMB6, PSMD8, BTG2, PNPO, CSGLCA-T, GLB1, PKM2, ATIC, APOD, CD36, CD24, SQLE, TDG, ALDH3B2, QPRT, GMFB, DHRS2, XYLT2, AKR1C3, AKR1A1, NDUFA8, CTSL2, WBSCR22, HIST1H1C
signal transduction	37	IRAK1, AGPAT2, RHOC, SQSTM1, GADD45G, GADD45A, BSG/CD147, RPS27L, PINK1, CALML5, CD14, SELS, GRN, IKBKG/NEMO, PRKAB1, LASP1, PEPD, C19ORF10/IL27, YWHAG (14-3-3 family), ATP1B3, ESRRA, GHITM, AHCYL1, LRP10, ZNF622, IMP3, CD36, CD24, GMFB, CEACAM6, C1QTNF6, WBP2, BCAS3, RAB17, CENTA1, FGD3, RAB25
Protein and membrane transport	28	STX16, AP1S1, SQSTM1, CD14, SDF4, GABARAPL2, HTATIP2, PEX16, SELS, PTTG1IP, GLTP, HTATIP2, ATP6V0E1, AP1S1, ATP6AP1, PSAP, SQSTM1, P4HA2, YIF1A, RABAC1, CLTA, CLTB, ARMET/MANF, TMED9, TRAPPC2L, SCARB2, RAB17, RAB25
potential oncogenes	27	CD276, PTTG1IP, EIF4G1, WBSCR22, LASP1, C19ORF10/IL27, ANAX2, RHOC, HOXC13, PKM2, ZDHHC8, IMP3, MED19, PINK1, BNIPL, CD24, CD36, TFPI, IFI6, CEACAM6, C1QTNF6, AKR1C3, CA12, CCDC6, BCAS3, PRSS23, RAB25
stress response	24	PRDX5, G6PD, SELS, RPS27L, BTG2, GADD45G, GADD45A, ISG20, DNAJB2, IRAK1, SDF4, SERPINA3, PINK1, SQSTM1, EIF4G1, ANAX2, HLA-H, ALDH3B2, AKR1C1, AKR1C3, CCDC6, ANAPC13, RDH11, RBM42
Inflammatory and immune response	22	PRDX5, SERPINA3, SERPINB1, ISG20, C19ORF10/IL27, IRAK1, CD276, IKBKG/NEMO, TOR3A, CD14, HLA-A, HLA-A29.1, B2M, FKBP2, SDC4, TSC22D3, IFI6, IFI27, DHRS2, CD24, MPZL2, BSG/CD147
mitochondrial function	14	ACADVL, ECH1, MRPL41, MRPS12, PSAP, PRDX5, ALDH4A1, CYB5R1, PINK1, IFI6, NDUFA8, IFI27, HARS2, DHRS2/HEP27
potential tumor suppressors	14	NME1, BTG2, HTATIP2/TIP30, GAD45A, BASP1, RNASET2, SERPINA5, HSPB8, ID3, WBP2, HRASLS3, RAB17, ANAPC13, ZBTB4
proteolysis, UPR and autophagy	14	FBXO22, PSMB4, PSMB6, PSMD8, SQSTM1, SELS, RPN1, RPN2, UBE2F, CTSL2, PEPD, PSMA1, DNAJB2, LAMP1
apoptosis	13	CYFIP2, PKM2, GADD45A, CD14, MRPL41, PSAP, FAM129B/MINERVA, GHITM, ZDHHC8, BNIPL, IFI27, HSPB8, PLSCR3
coagulation and wound healing	13	VCL, FAM129B, SERPINA3, CD14, SDC4, CD36, TFPI1, SERPINA5, CD24, PLSCR3, BCAS3, GRN
ER and Golgi localization and function	13	B2M/CD147, STX16, TMEM115, ARPC2, GABARAPL2, SDC4, PSAP, BSG, HLA-A, RPN1, PEX16, DPM3, NOMO2
transcriptional control	10	POLR2L, TSC22D1, CKAP4/p63, HOXC13, MED19, SCAND1, TSC22D3, ZNF263, ID3, ZBTB4
cytoskeletal organization	7	ARPC2, BASP1, MAP1LC3B, LASP1, FAM129B, ACTG2, CNO
Regulation of cell proliferation	7	CAPNS1, NME1, LAMC1, CD276, TMEM115, BTG2, VCL
peroxisome	3	PRDX5, ECH1, PEX16
translation initiation	2	EIF3I, EIF4G1
proton transport	2	ATP6V0E1, ATP6AP1
other/unknown	42	STOM, SQSTM1, CHPF, SNTB2, MGC71993, PH-4, TMEM4, LOC729776, SPNS1, C9ORF89, CCDC92, RPRC1, C17ORF90, FAM58A, NOMO2, C10ORF116, C1ORF128, C6ORF52, C8ORF33, HS.568928, LOC401115, NENF, UNK, ZNF79L, ANXA2P1, C9ORF169, FAM127A, HS.531457, SLC41A3, TMEM115, TUBA4A, MGC4677, KRT80, COMMD3, ATP9A, CHURC1, FER1L3, POLR3C, RFTN1, TMEM87A, KRT86, ZNF467

**Table 2:** Combined down-regulated processes during DOX selection.

Category	Count	Genes
nuclear functions/compartment	47	RPS15, PCNA, POLR2F, SNRPB, ORC6L, ATRIP, AKT1, SUMO3, EDF1, RPS27, PGRMC1, PTTG1, RNPS1, EGR1, MSH3, EDF1, FOSB, HSPB1, F2R/PAR-1, H3F3A, H2AFZ, HIST1H2AM, TOP2A, TOP2B, CDCA5, NUP62, HIST1H1D, HIST1H4C, HIST1H1B, HIST1H4E, HIST1H3C, PRC1, TIMELESS, RBMX, MCM3, MCM6, MCM7, DEK, VEZF1, CENPN, GINS2, E2F2, MSH6, NFIC, STAT2, MYB, SSBP1
Metabolic processes	37	UGCG, UGDH, SF3B3, RPS27A, SAE1, RPL17, LSM5, RPS5, POLA2, RPL22, RPL4, NONO, SHFM1, HPRT1, PAICS, CASP2, NACA, HNRNPD, RBMX, AKR1C2, FBL, RFC4, TOP2A, MCM7, MCM3, MCM6, MSH6, ATP6V1B1, MT2A, SSBP1, TPM1, SPDEF, ESD, GGCT, ANP32B, AKR1C2, AKR7A2
Signal transduction	29	CKLF, GFRA1, TUBB, STC2, NUP6, NET1, RAMP3, FLNB, RPS6KB1, DEK, NUP62, IGFBP5, RFC4, TOP2A, STAT2, EVL, RAB11A, PPP1CC, CAV1, F2R/PAR-1, AKT1, ATRIP, PCNA, GNB2L1/RACK1, SHCBP1, LFNG, PPP1CA, ROCK2, RET
RNA binding	25	XBP1, LSM1, LSM5, RPS5, RPL22, HNRPR, HNRNPD, RLP4, FBL, SF3B3, RBMX, NONO, NUP62, RPS27A, RPLP2, FAU, RPS19, RPS15, RPS10, RPS27, RPS24, RPL41, RPL35, RPL38, SNRPB
Ribosome biogenesis	25	TINP1, NOL11, RPL36AL, RPL4, RPL27A, GLTSCR2, RPL17, RPL22, RPS5, FAU, RPS19, RPS15, RPS10, RPS27, RPS24, RPL35, RPLP2, RPL38, RPL41, RPL32, WDR74, PGRMC1, POLR2F, HSPB1, GNB2L1/RACK1
stress response	21	VEZF1, TUBB, RFC4, TOP2A, MCM7, TPM1, MSH6, TIMELESS, STC2, NUP62, MT1A, MT2A, CKLF, RRM1, PCNA, HSPB1, ATRIP, AKT1, MSH3, F2R/PAR-1, KIAA0101/p15PAF
cell cycle progression	14	CSE1L, TIMELESS, TPD52L1, TOP2A, CDCA5, RPS27A, PRC1, MSH6, CCNB2, CAV1, ROCK2, AKT1, PTTG1/securin, PTTG3/meiotic securin
mitochondrial biogenesis	13	TOMM7, GGCT, SSBP1, TMEM14C, PMPCB, SDHA, PMPCA, COX4I1, COX7A2, RHOT2, ECHS1, CABC1, DNLZ
regulation of gene expression	12	RPS27A, TIMELESS, VEZF1, MYB, HNRNPD, DEK, NUP62, STAT2, NFIC, SFRS5, RNPS1, POLR2
apoptosis	9	CSE1L, NUP62, TOP2A, TUBB, RHOT2, AKT1, HSPB1, MIF, ARL6IP1
protein trafficking	9	RAB11A, NUP62, CSE1L, RAMP3, MYL6B, AKT1, GGA1, F2R/PAR-1, HGS
DNA replication	7	RFC4, MCM7, TOP2A, MCM3, MCM6, POLA2, GINS2
translation elongation	5	RPL17, RPS27A, RPL4, RPS5, RPL22
protein ubiquitination	5	SAE1, RPS27A, TTC3, PTTG1/mitotic securin, PTTG3/meiotic securin
tumor suppressors	5	CAV1, GLTSCR2, NBL1, CLUAP1, ITIH5
regulation of blood coagulation	3	F2R/PAR-1, EGR1, MATN2
sugar metabolism	3	AKT1, GAPDH, IMPA2
cell adhesion	2	CD44, PNN
cytoskeleton	2	TUBA1A, MYO5C
other	47	LOC642989, C14ORF173, HS.213061, STAG3L2, ILVBL, TMEM49, WDR54, CCNI, LOC340598, C15ORF15, C3ORF14, CCDC34, KIAA0101, SHFM1, TMEM64, LOC646723, TMEM109, PMPCA, LAIR, LOC441763, LOC401019, LOC643031, RN7SK, C19ORF31, LOC91561, HS.534061, OC643509, LOC645317, RN7SL, LOC399900, LOC440567, LOC440589, LOC441034, NAG18, TEX264, FAM177A1, IMAA, LOC388474, MGC16703, C1ORF63, LOC400963, LOC441246, LOC645895, NOL5A, C17ORF79, C20ORF117

### Coagulation pathway genes are differentially expressed at the mRNA level in DOX^sel^ cells

The genes defining the anticoagulation cascade are the focus of the remainder of this manuscript (Tables 1 and 2). The identified genes suggest that inhibition of thrombin activity and coagulation are associated with the establishment of DOX resistance. For example, TFPI1 mRNA, but not TFPI2, was up-regulated. Similarly, expression of the thrombin receptor, PAR-1/F2, was reduced. A network analysis of the coagulation genes identified in our study using the STRING database (version 9.05; http://string-db.org) demonstrated a large interacting network of many of the genes (Fig. S5A). While TFPI1 was highly embedded within this network, TFPI2's only link to this network was through tissue factor (F3). BCAS3 and PLCSR3 were part of a second network that was highly populated with cancer related and chromatin modifying genes (Fig. S5B). The controversial role of the thrombotic pathway in cancer progression, as it promotes cancer when active [2], [3], yet appears to generate untreatable forms of cancer when subsequently inhibited [11], [12], led us to focus our efforts on defining a role for TFPI1 expression in the establishment of DOX resistance.

### TFPI1 protein levels are elevated in DOX^Sel^ cells, leading to reduced thrombin protein

**Figure 3:**
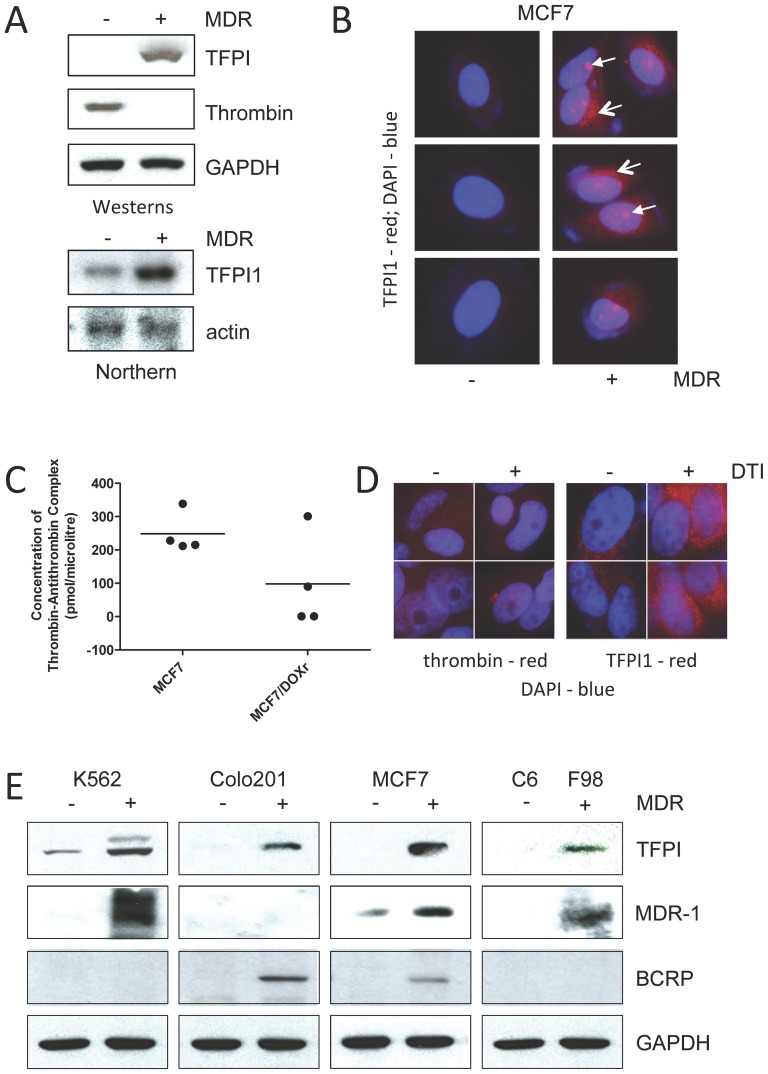
TFPI1 expression following DOX selection. (**A**) Westerns and Northerns were performed on cells before and after selection for DOX resistance. (**B**) Cells before and after DOX selection were stained with antibodies against TFPI1 (red) or DAPI for DNA (blue). Open arrows indicate perinuclear TFPI1 localization and closed arrows indicate nucleolar staining. (**C**) ELISA was used to detect thrombin protein in the spent media of MCF7 parental and DOX^Sel^ cells. (**D**) Parental MCF7 cells were stained before and after a 48-hour treatment of 20 µM Argatroban, a direct thrombin inhibitor (DTI), with antibodies against thrombin and TFPI1. Cells were stained with DAPI to visualize nuclear DNA. **E.** Four parental and drug resistant sets of cancer cells (K562 leukemia cells, Colo201 colorectal cells, MCF7 breast cancer cells and C6/F98 rat glioma cells) were prepared for Western analyses using antibodies shown.

The antithrombotic TFPI1 splice variant α (TFPI1α) mRNA was consistently and specifically elevated 3.4-fold in DOX^Sel^ cells, whereas mRNA from the TFPI1 splice variant β (TFPI1β) and from TFPI2 was essentially unchanged (Figure 1E, Table S4). To confirm this microarray result we performed a Northern analysis using total RNA extracted from parental and DOX^Sel^ cells. TFPI1 mRNA was indeed elevated in DOX^Sel^ cells (Fig. 3A, lower panels). This was also observed at the protein level using Western and immunocytochemistry (ICC) analyses (Figs. 3A, upper panels, and 3B). ICC analysis demonstrated that the overall TFPI1 protein accumulation was not at the plasma membrane, but localized within the nucleus in what appears to be nucleoli, and also in a perinuclear pattern (Fig. 3B). Our observations contrast with those previously reported in which TFPI1 was shown to be associated with the cell surface [13], [14]. In line with elevated TFPI1 levels, our results using Westerns, ELISA and ICC demonstrate that thrombin protein levels are indeed reduced in DOX^Sel^ cells (Figs. 3A and 3C; Fig. S6). Next, we asked what reciprocal effect inhibiting thrombin might have on TFPI1 protein levels. We treated parental MCF7 cells with 20 µM of the direct thrombin inhibitor (DTI) Argatroban for 48 hours (Fig. 3D). The cells were then prepared for ICC using antibodies against thrombin and TFPI1. Inhibition of thrombin activity by Argatroban correlated with increased intracellular TFPI1. Thus, a negative feedback loop may exist between TFPI1 and thrombin, such that DOX selection may either elevate TFPI1 or reduce thrombin protein levels.

To determine whether increased TFPI1 protein levels are a common feature of drug resistance, we prepared DOX resistant Colo201 colon cancer cells and K562 leukemia cells [32]. We also investigated rat C8 glioma cells and their drug resistant F98 partner cells. Protein lysates were prepared and analyzed with the antibodies shown (Fig. 3E). Strikingly, TFPI1 was low in all parental cells tested and significantly elevated in all DOX resistant cells. DOX resistance was confirmed by showing that either MDR-1 or BCRP protein levels, but not necessarily both, were elevated in DOX selected cells. Thus, TFPI1 appears to be a mainstay in DOX drug resistant cells.

### TFPI is associated with the development, but not maintenance, of DOX resistance

**Figure 4:**
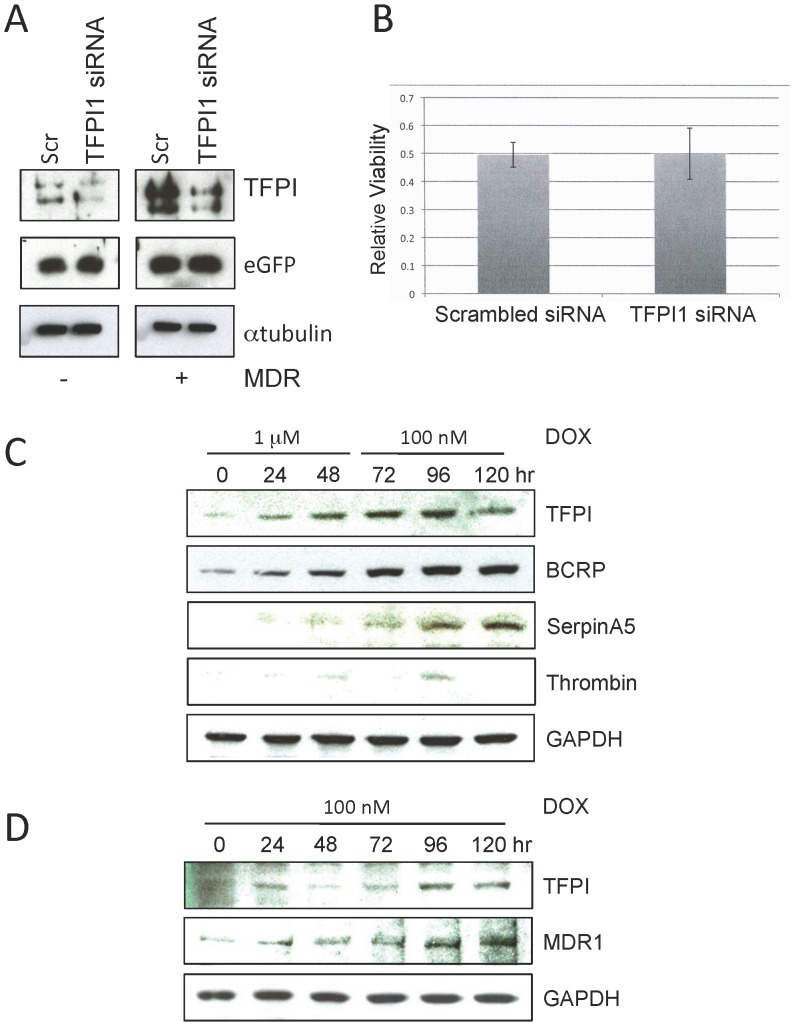
TFPI1 is expressed early in the drug selection process, but is not required for maintenance of the MDR state. (**A**) Parental and DOX selected MCF7 cells were treated with scrambled (S) or TFPI1 siRNA (+). A Western analysis using antibodies against TFPI1 show that silencing of TFPI1 was effective. eGFP was transfected along with the siRNA constructs and show that transfection efficiency was consistent. αTubulin was used as a load control. (**B**) Following 24 hours of siRNA treatment in DOX^Sel^ cells, 1 µM DOX was added for 48 hours. MTT was performed to determine cell killing. (**C**) Parental MCF7 cells were treated with 1 µM DOX for 48 hours, then maintained in 100 nM DOX for an additional 3 days. Protein samples were prepared every 24 hours and analyzed by Western blotting with the antibodies shown. (**D**) Parental MCF7 cells were incubated in 100 nM DOX for 5 days with samples removed every 24 hours for Western blotting with the antibodies indicated.

To clarify the role of TPFI1 in DOX resistance, we asked whether elevated TFPI1 levels are required for maintenance of DOX resistance. To test this hypothesis, we lowered TFPI1 levels before and after DOX selection using specific siRNA oligonucleotides, and subsequently measured the sensitivity of these cells to DOX re-exposure. Although TFPI1 was effectively silenced in parental and selected MCF7 cells (Fig. 4A), resistance to 1 µM DOX was unaffected (Fig. 4B). Therefore, elevated TFPI1 is apparently not required to maintain DOX resistance once established.

To determine whether TFPI1 is required for the establishment of DOX resistance, we attempted to silence TFPI1 expression during the DOX selection period. However, siRNA expression could not be effectively maintained throughout the prolonged selection process (data not shown). Instead, we performed a time course analysis of TFPI1 expression during DOX selection. Parental MCF7 cells were treated with 1 µM DOX for 48 hours, followed by maintenance in 100 nM DOX for an additional 3 days. Samples were taken for Western analyses every 24 hours. TFPI1 protein levels were found to be elevated after 24 hours of acute DOX exposure and reached an early plateau by 72 hours (Fig. 4C). Thrombin levels remained low throughout the experiment, indicating that DOX specifically induced TFPI1 protein expression, rather than reducing thrombin levels. The drug resistant marker BCRP showed the same early increase in protein expression as TFPI1. SerpinA5, a potential anticoagulaent, identified as up-regulated in our microarray, also showed an increase in protein levels, but it was delayed compared to BCRP and TFPI1. Our data supports the hypothesis that TFPI1 may play an early role in the transition to drug resistance.

To establish the importance of acute 1 µM DOX exposure on developing drug resistance, we repeated the time course in cells exposed to 100 nM DOX (Fig. 4D). A modest change only was observed in TFPI1 levels over the course of this experiment. Thus, maximal TFPI1 expression is not reached without high dose acute DOX exposure. In contrast, MDR-1 protein levels accumulated over time upon 100 nM DOX exposure, but this occurred much later in the time course. These experiments indicate that clinical outcomes may benefit from considering lower doses of chemotherapeutic drugs via a reduced risk of potential side effects, as well as decreased risk of developing drug resistance.

### Increased expression of HIF1α in DOX selected cells

**Figure 5:**
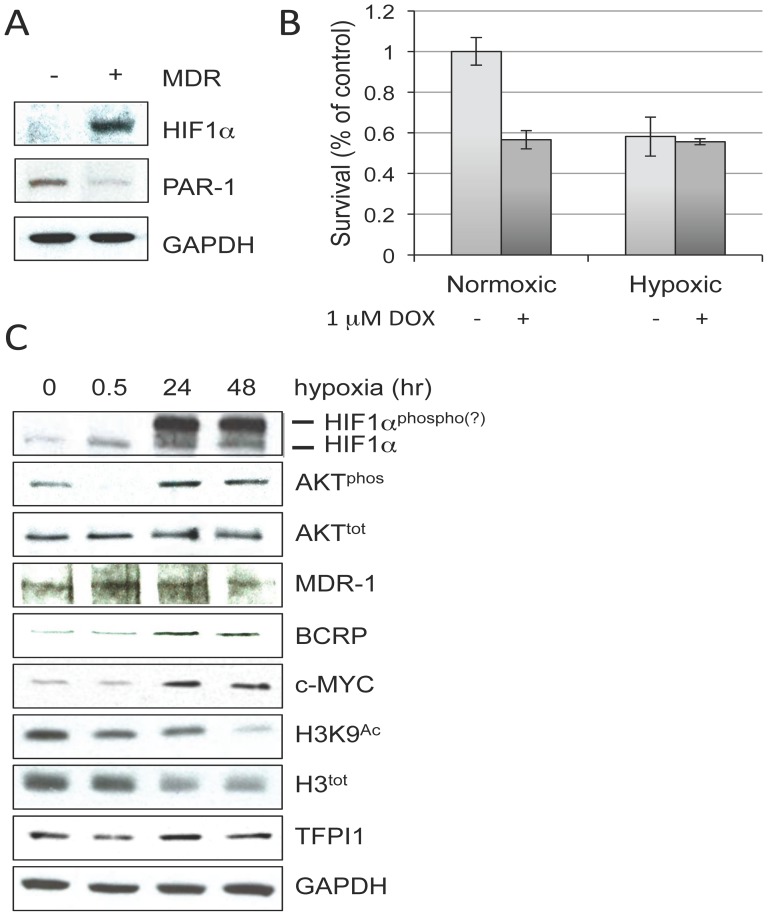
DOX selection induces HIF1α while hypoxia induces DOX resistance. (**A**) Protein lysates prepared from parental and DOX^Sel^ MCF7 cells were analyzed using HIF1α, PAR-1 and GAPDH antibodies. (**B**) Cells were exposed to 1% oxygen for 48 hours to induce hypoxia in the presence and absence of 1 µM DOX. Normoxic DOX treated cells (21% O_2_) were used as controls. Survival was determined using an MTT assay. The experiment was performed twice with MTT assays done in triplicate. Standard error of the mean is shown. (**C**) A hypoxia time course was performed with protein lysates prepared at the times indicated. Westerns were performed using the antibodies shown.

The failure of remission and the development of drug resistant cancer following antiangiogenic therapy has been linked to the unintentional induction of a hypoxic tumor microenvironment [41], [42]. It is estimated that between 40 and 50% of breast cancer tumors are immersed in a hypoxic environment [43]. Hypoxia Inducible Factor 1 alpha (HIF1α), often found elevated in solid tumors, responds to hypoxia and drives angiogenesis to promote new blood vessel growth to supply tumors [44], [45]. HIF1α can also be activated by nitric oxide, cytokines, insulin growth factors, expression of oncogenes, or mutation to tumor suppressors [46]. Considering this, we determined HIF1α levels in our cells. We observed that HIF1α protein was indeed elevated in DOX^Sel^ MCF7 cells, under normoxic conditions, compared to untreated parental cells (Fig. 5A). This was opposed to HIF1α mRNA levels, which were not altered by DOX^Sel^ in our microarray study (data not shown). PAR-1/F2R, a plasma membrane receptor that mediates thrombin-dependent angiogenesis, is often found expressed in tumor cells [47] and has been shown to protect hypoxic cells from cell death [48]. In our study, PAR-1 mRNA was reduced in our microarray of DOX^Sel^ cells, which we validated at the protein level (Fig. 5A). Down-regulation of PAR-1 in DOX^Sel^ MCF7 cells provides further evidence that thrombin is not involved in the establishment of DOX resistance, and is consistent with the creation of a hypoxic-like environment that drives secondary angiogenesis through HIF1α in a thrombin/PAR-1-independent manner.

### Hypoxia induces DOX resistance

To determine whether the hypoxic-like environment observed in DOX selected MCF7 cells plays a role in establishing DOX resistance, we grew MCF7 parental cells in 1% O_2_ for 48 hours to induce hypoxia, in the presence or absence of 1 µM DOX. Only 60% of the cells survived hypoxia, compared to the normoxia control (21% O_2_; Fig. 5B). This is consistent with tumors that favor fermentation and respiration for energy generation due to limited O_2_ and glucose within the tumor microenvironment, suffering when either glucose or O_2_ are deprived [49]. While roughly 58% of normoxic cells survived 1 µM DOX, all of the hypoxic cells survived DOX exposure (Fig. 5B). The epistatic interaction between hypoxia and DOX treatment suggests DOX and hypoxia work through similar cytotoxic pathways.

To confirm the cells were hypoxic, we extracted proteins from cells exposed to hypoxia at various timepoints for Western analyses. HIF1α protein levels were indeed elevated (Fig. 5C), as previously reported [50]. After 30 minutes, HIF1α levels began to increase, and after 24 hours HIF1α was dramatically increased and was modified. HIF1α can be phosphorylated by several kinases, including PKA and p38 [51], [52]. Here, we showed that AKT may also be involved, as the AKT phosphorylation state, and presumably activity, was increased with hypoxia (Fig. 5C). To further validate whether hypoxia drives cells towards drug resistance, we showed that MDR-1, BCRP and c-MYC protein levels all increased with hypoxic treatment. Moreover, considering that reduced H3 acetylation is linked with the establishment of aggressive tumors (Fig. 1B) [53], [54], total and acetylated H3 were also reduced by hypoxia. TFPI1, however, was not affected by hypoxia. Thus, although DOX selection induces both HIF1α and TFPI1, induction of hypoxia does not coincide with increased TFPI1 protein.

### Overexpression of TFPI1 in parental MCF7 cells induces HIF1α protein expression and increases resistance to DOX

**Figure 6:**
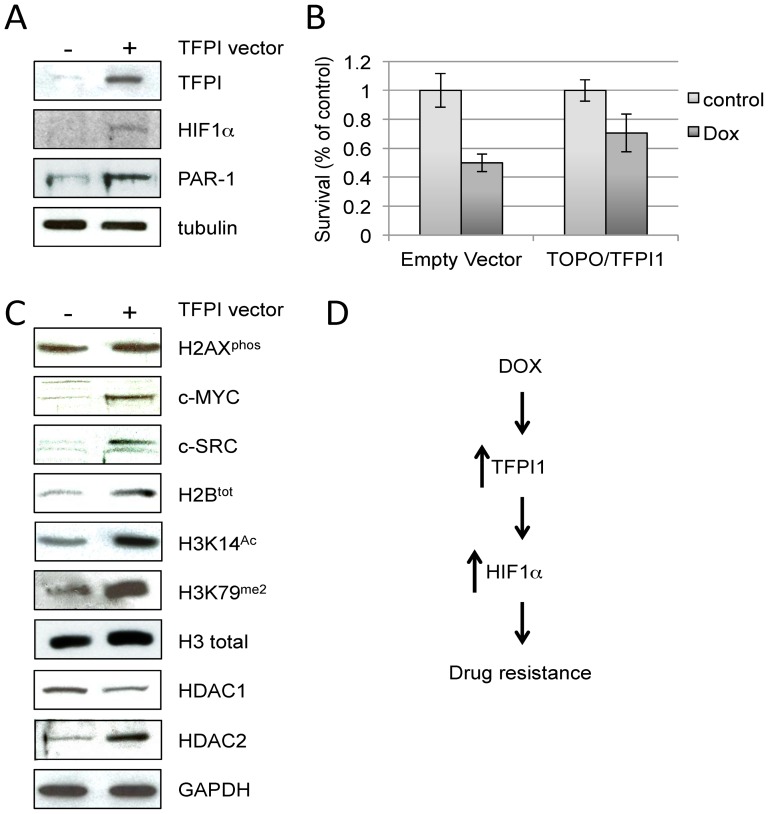
TFPI1 overexpression increases DOX resistance and levels of procancer proteins, consistent with TFPI1 playing an early role in the MDR transition. (**A**) Parental MCF7 cells were transfected with an empty vector construct, or a construct overexpressing TFPI1. After 24 hours, the cells were harvested and prepared for protein analyses using the antibodies shown. (**B**) Cells were transfected with a TFPI1 expressing vector or the empty vector, and left for 24 hours. Next, the cells were treated with 1 µM DOX for an additional 24 hours. MTT was performed to determine cell killing. The MTT assay was done in triplicate with the standard error of the mean indicated. (**C**) The lysates used above were used to assess levels of the proteins shown. (**D**) A schematic representation of a possible model for how DOX exposure leads to DOX resistance. Increased TFPI1 protein could be p53-dependent (see Discussion), while elevated HIF1α protein could be through HIF1α stabilization.

MCF7 DOX^Sel^ cells in culture are grown in a monolayer and are not hypoxic. To determine whether the anticoagulation pathway has the capacity to induce a hypoxic-like state under normoxic conditions, we first performed a network analysis using the STRING database (version 9.05). This analysis indicated that HIF1α and TFPI1 may be closely connected functionally through p53 and thrombospondin 1 (THBS1; Fig. S7; THBS1 and p53 (TP53) mRNAs were not altered in our study). We hypothesized that DOX exposure may trigger a hypoxic-like environment via TFPI1. To test this, we transfected parental MCF7 cells with a TFPI1 overexpression vector; TFPI1 protein was elevated in these cells (Fig. 6A; the vectors were generous gifts from N. Iversen, Oslo University). Cells expressing TFPI1 exhibited increased HIF1α and PAR-1 expression (Fig. 6A). The increased PAR-1 protein levels were consistent with the notion that TFPI1 alone can promote early angiogenesis through the survival mechanisms associated with PAR-1 [48], [54]. The concomitant expression of HIF1α and PAR-1 when TFPI1 is overexpressed may facilitate the onset of drug resistance. To test this idea, MCF7 parental cells expressing TFPI1 for 24 hours, or the control empty vector, were treated with 1 µM DOX for an additional 48 hours. Cells overexpressing TFPI1 had a higher survival rate than control cells (Fig. 6B). In support of this, we observed that c-MYC and c-SRC, proteins elevated in breast cancers [55], were also elevated following TFPI1 expression (Fig. 6C).

### Histone modifications are induced by TFPI1 expression in parental MCF7 cells

Alterations to chromatin structure are tightly linked to cancer progression [53], [56]. Thus, we examined histone post-translational modifications following TFPI1 overexpression in parental MCF7 cells. Certain histone modifications respond to DNA damage, including phosphorylated H2AX (H2AX^phos^) [40]. A second histone modification, H3 lysine 79 dimethylation (H3K79^me2^), is likely involved in global genomic repair (GGR) and may activate DNA damage checkpoints in yeast [57]. Although H2AX^phos^ was markedly elevated after DOX selection (Fig. 1B), H2AX^phos^ was unaffected by experimentally raising TFPI1 protein levels (Fig. 6C). This is in contrast to elevated H3K79^me2^ levels in TFPI1 expressing cells. These observations suggest that the DNA damage checkpoint may be activated following TFPI1 expression, prior to H2AX^phos^, suggesting a possible temporal order of events leading in drug resistance.

TFPI1 expression for 24 hours *in vitro* leads to increased levels of selected proteins. To determine whether increased expression of proteins following 24 hours of TFPI1 expression may be facilitated by altered histone post-translational modifications, we performed westerns with antibodies that specifically recognize H3K14^Ac^, a modification tightly linked to transcriptional activation [58]. We observed increased total H3 and H2B levels, as well as H3K14^Ac^ (Fig. 6C). Proteins that control histone deacetylation, such as HDAC1 and HDAC2, are elevated in cancer cells [59], [60]. HDAC1 and HDAC2 repressive transcriptional activity is generally carried out by HDAC1/HDAC2 heterodimers that interact with a number of corepressor complexes [61]. We observed that HDAC1 levels were reduced, while HDAC2 levels were increased following TFPI1 expression (Fig. 6C). This suggests that HDAC1/HDAC2 complex stoichiometry may be altered when TFPI1 is overexpressed. Interestingly, a recent report demonstrated that HDAC1 and HDAC2 homodimers are preferentially formed during mitotic progression in MCF7 cells [62]. Taken together, our data strongly indicates that elevated TFPI1 protein levels occur in response to DOX exposure, thereby increasing the expression of multiple genes involved in establishing MDR, such as HIF1α (Fig. 6D).

### Human tumor datasets with high BCRP and MDR-1 expression also exhibit elevated TFPI1 expression

**Figure 7:**
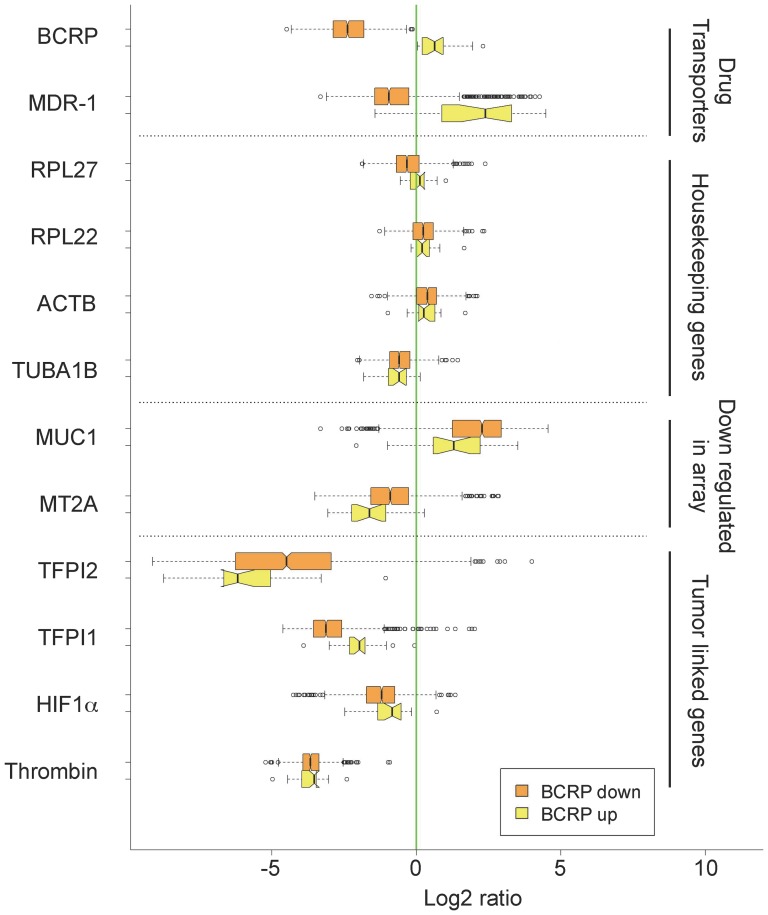
TFPI1 mRNA is elevated in human tumor samples when BCRP and MDR-1 are also elevated. 1223 datasets from patients with breast (529), ovarian (539) or colon (155) tumors were gathered from an Agilent expression study. mRNA expression of BCRP was followed in these samples. Those with reduced and increased expression levels were segregated and pooled forming the sets “BCRP down” (1204) and “BCRP up” (19), respectively. In each pool, the expression of the shown genes was determined. See methods for an explanation of the box plot.

To determine whether TFPI1 expression correlated with potential MDR human patients, we screened 1204 patient expression datasets (529 breast, 539 ovarian and 155 colon cancer patients) gathered from Agilent microarray expression assays. Differential gene expression was determined by comparing all tumor samples with Universal Reference RNA (URR), which is high-quality total RNA from ten cell lines. Thus, where mean expression is below negative in Fig. 7, and vice versa, expression of that gene in the tumor is below the expression of that gene in the URR sample. The expression of BCRP (ABCG2) was determined in all sets. We pooled datasets where BCPR expression was high (BCRP up) and where BCRP expression was low (BCRP down). A total of 19 datasets from the initial 1223 datasets met the condition of elevated BCRP. We chose BCRP as a marker for MDR, as it is highly correlated with aggressive breast cancer [63]. As a point of validation, when BCRP was up, so was MDR-1 expression, providing support that these tumors likely represented MDR tumors. As controls we assessed the expression of several housekeeping genes (RPL27, RPL22, ACTB, and TUBA1B), which did not depend on BCRP expression, nor were they significantly different from the URR samples (Fig. 7). We next tested two genes that were down-regulated in our microarray analysis, MUC1 and MT2A. Both of these were down-regulated in patient samples when BCRP was up-regulated. Lastly, we assessed the expression levels of RNAs critical to our study. When BCRP was up, so was TFPI1. As expected from our study, TFPI2, predicted to play a tumor suppressor role in tumor development, was down-regulated when BCRP was up. Thrombin levels were low, and did not change when BCRP was up. In our *in vitro* studies, HIF1α protein expression was elevated in MDR cells. However, HIF1α mRNA expression in tumors was relatively unchanged *in vivo* when BCRP was elevated. This is likely due to the fact that HIF1α activity is controlled primarily through post-translational mechanisms, such as through targeted degradation via the von Hippel-Lindau (VHL) ubiquitin-protein ligase under normoxic conditions [51]. Since HIF1α mRNA expression did not change in our MDR microarray, it is likely that increased HIF1α is through stabilization of the protein following DOX exposure. Taken together, results from human tumors where BCRP and MDR-1 mRNA expression is elevated, supports our hypothesis that TFPI1 is involved with the establishment of MDR.

## Discussion

A temporal transcript profiling strategy was used to identify genes whose expression was specifically altered when MCF7 breast cancer cells were selected for drug resistance to a commonly used chemotherapy agent, Doxorubicin (DOX). We identified 73 down-regulated and 47 up-regulated genes in response to the selection process (Table S1). We applied a more comprehensive analysis comparing differential gene expression profiles following the initial acute DOX exposure phase with the expression profiles following the two-week chronic DOX exposure phase. This identified genes differentially expressed during the acute phase that remained so during the chronic phase, and those genes that became differentially expressed specifically during the chronic phase (201 genes up-regulated and 209 down-regulated; Table S4). This analysis revealed elevations in genes involved in various stress responses, including those that induce a hypoxic-like environment and those that provide survival benefits during hypoxia. This provided a potential explanation for how tumor cells can develop resistance to antiproliferative agents so rapidly.

### TFPI1 elevates the transcription of key cancer promoters

Anticancer drugs target many unique aspects of tumor biology to specifically prevent tumor progression, while attempting to minimize effects on noncancerous tissues. One approach has been to limit the formation of new blood supply stimulated by growing tumors using antiangiogenic compounds. This approach has been found to initially halt tumor growth, but the subsequent induction of hypoxia is believed to inevitably lead to the later development of invasive and metastatic cancers [41], [42]. Antiangiogenic agents typically inhibit thrombin, a potent inducer of VEGF, which drives angiogenesis and resistance to radiation therapy and chemotherapy [8]. Inhibition of thrombin or VEGF may promote an intratumoral hypoxic environment, which may ultimately favor the re-establishment of angiogenesis and new blood vessel formation. Here, we show that TFPI1 is up-regulated during DOX selection of all cancer cell lines examined (Fig. 3E). Furthermore, we also observed TFPI1 to be specifically elevated in patient tumor datasets where transcripts encoding the MDR specific proteins BCRP and MDR-1 were elevated (Fig. 7). This indicates a strong association of TFPI1 with MDR tumors. As a natural inhibitor of the TF/thrombin pathway, increased TFPI1 protein levels may contribute to the establishment of an initial hypoxic-like environment in response to DOX, which is supported by elevated HIF1α, as well as c-SRC, c-MYC, HDAC2 and PAR-1 protein expression following TFPI1 overexpression (Figs. 6A and 6C). How DOX increases TFPI1 mRNA, and protein, remains unknown. A network analysis (Fig. S7) presents a possible mechanism. Stress conditions, such as DOX exposure, could activate p53. Thrombospondin 1 (THBS1) is a target of p53 that forms a complex with TFPI1, increasing its inhibitory effects on Factor VIIa·TF [64], [65].

A recent microarray paper describing overexpression of TFPI1α in SK-BR-3 breast cancer cells provides further evidence that TFPI1α does indeed drive the proliferation of cancer cells [22]. The top genes expressed in that study were almost all associated with cancer cell proliferation, with a heavy reliance on interferon pathway genes. Expression of inflammatory and immune response genes is often elevated in tumor cells [66]. In fact, the induction of angiogenesis by hypoxia can also be mediated through the expression of inflammatory cytokines and interferons [67]. Our observation that a variety of inflammatory and immune response genes were elevated during DOX selection (Table 1) is consistent with the establishment of an angiogenic environment that is conducive to ongoing invasive and malignant growth.

### Hypocoagulation is important for the onset of drug resistance

Our hypothesis that TFPI1 is a critical component of the drug resistance mechanism is supported by the detection of the differential expression of many genes critical to coagulation regulation, such as SerpinA5 and CD36 (thrombospondin receptor). SerpinA5, up-regulated in our microarray study, and at the protein level during DOX selection (Fig. 4C), is a serine protease that associates with cellular membranes and inhibits activated protein C (APC) [68]. APC is a natural anticoagulant, like TFPI1, that may serve as an important anticancer target [69]. Thus, the result of APC inhibition by SerpinA5 is consistent with generation of a procoagulant state. However, contrary to the idea of APC playing a purely anticoagulant role, recent work indicates that APC cleaves plasma membrane-associated TFPI1, blocking TFPI1's ability to inhibit thrombin [70]. Thus, SerpinA5 may help maintain TFPI1 on the cell surface by inhibiting APC-dependent cleavage of TFPI1.

In a network analysis (STRING, version 9.05) of the anticoagulation genes identified in our study a great deal of connectivity with thrombospondin 1 (THBS1) was observed (Fig. S5A). As discussed above, TFPI1 may be connected to HIF1α expression through THBS1 (Fig. S7). Although THBS1 was not identified as altered in our microarray analysis, its receptor, CD36, also an anticoagulent, was induced 2.6 fold (Table S4). THBS1 peptides have been considered as a therapeutic approach to block *de novo* blood vessel formation supplying tumors [71]. Increased levels of CD36 during chronic DOX exposure are consistent with the development of a hypocoagulant environment that would trigger a hypoxic-like response, stabilizing HIF1α protein, and subsequent DOX resistance. TFPI1 appears to be the middle-man tying acute DOX exposure with anticoagulation. TFPI1 expression is induced early, within 24 hours of acute DOX exposure (1 µM), but not when exposed to chronic doses (100 nM) only (Figs. 4C and 4D). The observation that approximately 40% of parental MCF7 cells do not survive hypoxia (Fig. 5B) is consistent with the Warburg effect where depriving cells of either oxygen or glucose will have a negative impact on tumor cells. It was proposed that tumor cells must be deprived of both oxygen and glucose to effectively kill them [49]. Furthermore, since DOX no longer has an effect on cells that survive hypoxia (Fig. 5B), one interpretation of this observation is that DOX and hypoxia induce similar drug resistant mechanisms, by blocking access of the tumor to oxygen.

### Conclusions

The clinical relevance of using combination chemotherapy to minimize the induction of drug resistance becomes clear as one considers the effects of anticancer drugs that involve stress induction as their primary objective, especially hypoxia. Aggressive cancers may use the anticancer drug's own endgame to resurrect angiogenic transcriptional programs, resulting in the inevitable development of multiple drug resistant tumor cells. Recent studies demonstrate that using angiogenesis and HIF1α inhibitors in combination offer better outcomes than either agent alone [41]. In conclusion, a thorough understanding of how drug resistance occurs, and the processes involved, whether through innate mechanisms, or through a response to anticancer treatment, will allow the rational design of more strategically targeted therapies to have the maximum effect on the tumor cell, while limiting negative downstream short term and long term side effects.

## Supporting Information

Figure S1 **A schematic representation of our experimental plan for analyzing differentially expressed genes.** 1 and 2 refer to genes that do not undergo expression changes under acute exposure to 1 µM DOX for 48 hours. 3 and 4 refer to genes that are up or downregulated, respectively, during the acute phase. 5 and 6 refer to genes that do not change during chronic exposure to 100 nM DOX for 2 weeks, whereas 7 and 8 define genes that are down or up-regulated, respectively, during chronic exposure. Genes that fit, for example, a 3–5 category are up-regulated during acute exposure and remain so during the chronic phase.
(DOCX)

Figure S2 **A gene ontology determination of cellular processes up-regulated (A) and down-regulated (B) during acute exposure to 1 µM DOX.**
(DOCX)

Figure S3 **A gene ontology determination of cellular processes up-regulated (A) and down-regulated (B) during chronic exposure to 100 nM DOX.**
(DOCX)

Figure S4 **A Venn diagram comparing the myriad of ‘metabolic processes’ associated with up- or down-regulated gene expression.** Only two of the 14 metabolic functions overlapped, protein and steroid metabolism.
(DOCX)

Figure S5 **Network analysis of coagulation genes differentially expressed during DOX selection of MCF7 cells.** (**A**) Using String 9.05 (string-db.org), the gene interactions among thrombin regulatory pathways were plotted using the action view option. Genes identified in our microarray, such as TFPI1, CD36, CD44, F2R, SERPIN5A, EGR1, and SDC4, are part of a much larger network. Select gene names are given for clarity. TFPI2 was added to illustrate that TFPI1 and TFPI2 interact with very different networks that intersect only at F3. (**B**) BCAS3 and PLSCR3 do not interact with the thrombin network, but interact together in a cancer related network. Select gene names are shown for clarity.
(DOCX)

Figure S6 **Immunohistochemistry analysis of thrombin protein expression in parental and DOX selected MCF7 cells.** DNA in each cell was stained with DAPI in blue, while thrombin was imaged with red. Thrombin expression in parental cells was low, and barely above background in selected cells.
(DOCX)

Figure S7 **Network analysis of TFPI1 connections to HIF1α.** Using String 9.05 (string-db.org), TFPI1 and HIF1α are found to be part of network via p53 (TP53) and the anticoagulant Thrombospondin 1 (THBS1). p53 activates the transcription of THBS1 [64], [72], which forms a complex with TFPI1 and increases its inhibitory effects on Factor VIIa·TF [65]. p53 binds to unphosphorylated HIF1α, leading to p53-dependent apoptosis [73]. SIRT1 may have an inhibitory effect on TFPI1 activity by deacetylating p53 leading to inactivation of p53 under DNA damaging conditions [74]. The networks shown in Figs. S4A and S4B connect to this network through TP53 and THBS1.
(DOCX)

Table S1 **Differential gene expression changes following selection of MCF7 cells for DOX resistance.** Genes differentially expressed over 2-fold (FC) are shown. The numbers in parenthesis reflect the total number of genes in each list. “DOX on MCF7” indicates that gene expression changes were compared between DOX selected MCF7 cells and parental MCF7 cells. The array did not contain probes for MDR-1 or BCRP.
(DOCX)

Table S2 **Gene expression changes in MCF7 cells comparing parental cells after a 48 hour treatment with 1 µM DOX, and comparing DOX selected cells with cells after the 48 hour treatment.** Edges 3, 4, 7 and 8 refer to the numbering system shown in Fig. S1.
(DOCX)

Table S3 **Reversion of gene expression changes following the 2-week chronic exposure to 1 nM DOX.** Edges 3–7 and 4–8 refer to the numbering system described in Fig. S1.
(DOCX)

Table S4 **Gene expression changes defining the acute and chronic phases of selection for DOX resistant MCF7 cells.** Edges 1–7, 2–4, 3–5 and 4–6 refer to the numbering system described in Fig. S1. For example, Edge 1–7 refers to genes that are unchanged during acute exposure and down-regulated during chronic exposure.
(DOCX)

Table S5 **Up-regulated processes during acute DOX exposure.**
(DOCX)

Table S6 **Up-regulated processes during chronic DOX exposure.**
(DOCX)

Table S7 **Down-regulated processes during acute DOX exposure.**
(DOCX)

Table S8 **Down-regulated processes during chronic DOX exposure.**
(DOCX)

Table S9 **A comparison of up- and down-regulated metabolic processes associated with selection of DOX resistant MCF7 cells.** The genes that make up each metabolic process are listed in the right column.
(DOCX)
