# Supplementary material for: TFPI1 Mediates Resistance to Doxorubicin in Breast Cancer Cells by Inducing a Hypoxic-Like Response
Source: PLoS One. 2014 Jan 28;9(1):e84611. doi: 10.1371/journal.pone.0084611 (PMC3904823; doi:10.1371/journal.pone.0084611)
Supplement: Figure S1 — A schematic representation of our experimental plan for analyzing differentially expressed genes. 1 and 2 refer to genes that do not undergo expression changes under acute exposure to 1 µM DOX for 48 hours. 3 and 4 refer to genes that are up or downregulated, respectively, during the acute phase. 5 and 6 refer to genes that do not change during chronic exposure to 100 nM DOX for 2 weeks, whereas 7 and 8 define genes that are down or up-regulated, respectively, during chronic exposure. Genes that fit, for example, a 3–5 category are up-regulated during acute exposure and remain so during the chronic phase. (DOCX) [file pone.0084611.s001.docx]

**Supplemental Figure 1** **A schematic representation of our experimental plan for analyzing differentially expressed genes.** 1 and 2 refer to genes that do not undergo expression changes under acute exposure to 1 μM DOX for 48 hours. 3 and 4 refer to genes that are up or downregulated, respectively, during the acute phase. 5 and 6 refer to genes that do not change during chronic exposure to 100 nM DOX for 2 weeks, whereas 7 and 8 define genes that are down or up-regulated, respectively, during chronic exposure. Genes that fit, for example, a 3-5 category are up-regulated during acute exposure and remain so during the chronic phase.
