# Supplementary material for: TFPI1 Mediates Resistance to Doxorubicin in Breast Cancer Cells by Inducing a Hypoxic-Like Response
Source: PLoS One. 2014 Jan 28;9(1):e84611. doi: 10.1371/journal.pone.0084611 (PMC3904823; doi:10.1371/journal.pone.0084611)
Supplement: Figure S3 — A gene ontology determination of cellular processes up-regulated (A) and down-regulated (B) during chronic exposure to 100 nM DOX. (DOCX) [file pone.0084611.s003.docx]

**Supplementary Figure 3** A gene ontology determination of cellular processes up-regulated (**A**) and down-regulated (**B**) during chronic exposure to 100 M DOX.
