# Supplementary material for: TFPI1 Mediates Resistance to Doxorubicin in Breast Cancer Cells by Inducing a Hypoxic-Like Response
Source: PLoS One. 2014 Jan 28;9(1):e84611. doi: 10.1371/journal.pone.0084611 (PMC3904823; doi:10.1371/journal.pone.0084611)
Supplement: Figure S4 — A Venn diagram comparing the myriad of ‘metabolic processes’ associated with up- or down-regulated gene expression. Only two of the 14 metabolic functions overlapped, protein and steroid metabolism. (DOCX) [file pone.0084611.s004.docx]

**Supplementary Figure 4** **A Venn diagram comparing the myriad of ‘metabolic processes’ associated with up- or down-regulated gene expression.** Only two of the 14 metabolic functions overlapped, protein and steroid metabolism.
