# Supplementary material for: TFPI1 Mediates Resistance to Doxorubicin in Breast Cancer Cells by Inducing a Hypoxic-Like Response
Source: PLoS One. 2014 Jan 28;9(1):e84611. doi: 10.1371/journal.pone.0084611 (PMC3904823; doi:10.1371/journal.pone.0084611)
Supplement: Figure S5 — Network analysis of coagulation genes differentially expressed during DOX selection of MCF7 cells. (A) Using String 9.05 (string-db.org), the gene interactions among thrombin regulatory pathways were plotted using the action view option. Genes identified in our microarray, such as TFPI1, CD36, CD44, F2R, SERPIN5A, EGR1, and SDC4, are part of a much larger network. Select gene names are given for clarity. TFPI2 was added to illustrate that TFPI1 and TFPI2 interact with very different networks that intersect only at F3. (B) BCAS3 and PLSCR3 do not interact with the thrombin network, but interact together in a cancer related network. Select gene names are shown for clarity. (DOCX) [file pone.0084611.s005.docx]

APPBP2 – amyloid beta precursor protein binding protein

PLSCR3 – phospholipid scramblase 3

ESR1 – estrogen receptor

BCAS3 – breast carcinoma amplified sequence 3

BCAS4 – breast carcinoma amplified sequence 4

BRCA1 – breast cancer 1

TP53 – p53

MTA1 – metastasis associated 1

HDAC1 – histone deacetylase 1

EP300 – histone acetyltransferase

KDM4C – histone H3K9 and H3K36 demethylase

F2 – thrombin

F2R – thrombin receptor

F3 – tissue factor (TF)

THBS1 – thrombospondin

CD36 – thrombospondin receptor

TFPI – tissue factor pathway inhibiitor 1

TFPI2 – tissue factor pathway inhibiitor 2

THBD – thrombomodulin

FGA – fibrinogen

F5 – coagulation factor V

F7 – coagulation factor VII

**Supplemental Figure 5** **Network analysis of coagulation genes differentially expressed during DOX selection of MCF7 cells.** (**A**) Using String 9.05 (string-db.org), the gene interactions among thrombin regulatory pathways were plotted using the action view option. Genes identified in our microarray, such as TFPI1, CD36, CD44, F2R, SERPIN5A, EGR1, and SDC4, are part of a much larger network. Select gene names are given for clarity. TFPI2 was added to illustrate that TFPI1 and TFPI2 interact with very different networks that intersect only at F3. (**B**) BCAS3 and PLSCR3 do not interact with the thrombin network, but interact together in a cancer related network. Select gene names are shown for clarity.
