# Supplementary material for: TFPI1 Mediates Resistance to Doxorubicin in Breast Cancer Cells by Inducing a Hypoxic-Like Response
Source: PLoS One. 2014 Jan 28;9(1):e84611. doi: 10.1371/journal.pone.0084611 (PMC3904823; doi:10.1371/journal.pone.0084611)
Supplement: Figure S6 — Immunohistochemistry analysis of thrombin protein expression in parental and DOX selected MCF7 cells. DNA in each cell was stained with DAPI in blue, while thrombin was imaged with red. Thrombin expression in parental cells was low, and barely above background in selected cells. (DOCX) [file pone.0084611.s006.docx]

**Supplementary Figure 6** **Immunohistochemistry analysis of thrombin protein expression in parental and DOX selected MCF7 cells.** DNA in each cell was stained with DAPI in blue, while thrombin was imaged with red. Thrombin expression in parental cells was low, and barely above background in selected cells.
