# Supplementary material for: TFPI1 Mediates Resistance to Doxorubicin in Breast Cancer Cells by Inducing a Hypoxic-Like Response
Source: PLoS One. 2014 Jan 28;9(1):e84611. doi: 10.1371/journal.pone.0084611 (PMC3904823; doi:10.1371/journal.pone.0084611)
Supplement: Figure S7 — Network analysis of TFPI1 connections to HIF1α. Using String 9.05 (string-db.org), TFPI1 and HIF1α are found to be part of network via p53 (TP53) and the anticoagulant Thrombospondin 1 (THBS1). p53 activates the transcription of THBS1 [64], [72], which forms a complex with TFPI1 and increases its inhibitory effects on Factor VIIa·TF [65]. p53 binds to unphosphorylated HIF1α, leading to p53-dependent apoptosis [73]. SIRT1 may have an inhibitory effect on TFPI1 activity by deacetylating p53 leading to inactivation of p53 under DNA damaging conditions [74]. The networks shown in Figs. S4A and S4B connect to this network through TP53 and THBS1. (DOCX) [file pone.0084611.s007.docx]

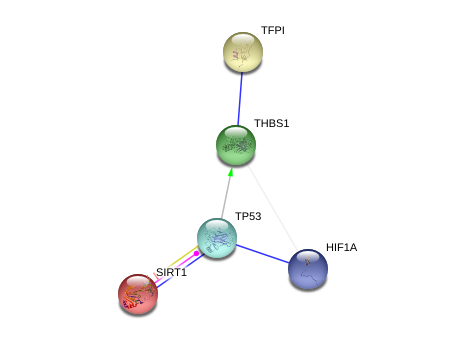


**Supplemental Figure 7** **Network analysis of TFPI1 connections to HIF1α.** Using String 9.05 (string-db.org), TFPI1 and HIF1α are found to be part of network via p53 (TP53) and the anticoagulant Thrombospondin 1 (THBS1). p53 activates the transcription of THBS1 (Dameron et al., 1994; Volpert et al., 1997), which forms a complex with TFPI1 and increases its inhibitory effects on Factor VIIa·TF (Mast et al., 2000). p53 binds to unphosphorylated HIF1α, leading to p53-dependent apoptosis (Suzuki et al. 2001). SIRT1 may have an inhibitory effect on TFPI1 activity by deacetylating p53 leading to inactivation of p53 under DNA damaging conditions (Stünkel and Campbell, 2011). The networks shown in Suppl. Figs. 4A and 4B connect to this network through TP53 and THBS1.
