# Supplementary material for: TFPI1 Mediates Resistance to Doxorubicin in Breast Cancer Cells by Inducing a Hypoxic-Like Response
Source: PLoS One. 2014 Jan 28;9(1):e84611. doi: 10.1371/journal.pone.0084611 (PMC3904823; doi:10.1371/journal.pone.0084611)
Supplement: Table S1 — Differential gene expression changes following selection of MCF7 cells for DOX resistance. Genes differentially expressed over 2-fold (FC) are shown. The numbers in parenthesis reflect the total number of genes in each list. “DOX on MCF7” indicates that gene expression changes were compared between DOX selected MCF7 cells and parental MCF7 cells. The array did not contain probes for MDR-1 or BCRP. (DOCX) [file pone.0084611.s008.docx]

**Supplementary Table 1 Differential gene expression changes following selection of MCF7 cells for DOX resistance.** Genes differentially expressed over 2-fold (FC) are shown. The numbers in parenthesis reflect the total number of genes in each list. “DOX on MCF7” indicates that gene expression changes were compared between DOX selected MCF7 cells and parental MCF7 cells. The array did not contain probes for MDR-1 or BCRP.

| **DOX on MCF7 (47) - UP** | |  |  | **DOX on MCF7 (73) - DOWN** | |  |
| --- | --- | --- | --- | --- | --- | --- |
|  |  |  |  |  |  |  |
| **Probe ID** | **TargetID** | **FC** |  | **Probe ID** | **TargetID** | **FC** |
| 3990170 | IFI27 | 5.6 |  | 6770438 | HIST1H1B | -5.4 |
| 4230201 | CDKN1A | 5.4 |  | 3890349 | HIST1H4C | -5.1 |
| 5090671 | GDF15 | 4.2 |  | 1510291 | PTTG1 | -5.0 |
| 2140121 | BASP1 | 3.7 |  | 450615 | MT2A | -4.4 |
| 5090215 | IFI6 | 3.7 |  | 6200402 | MT1A | -4.1 |
| 3310538 | CD36 | 3.6 |  | 2070494 | PRC1 | -4.1 |
| 7150634 | APOD | 3.5 |  | 2230619 | HIST1H4E | -4.0 |
| 2940291 | QPRT | 3.4 |  | 6520424 | HIST1H3C | -3.8 |
| 4150189 | CTSL1 | 3.1 |  | 7650026 | MUC1 | -3.5 |
| 7400377 | CEACAM6 | 3.1 |  | 4280017 | FOS | -3.3 |
| 4260386 | CTSL1 | 3.0 |  | 2680450 | HIST1H1D | -3.2 |
| 1010246 | IFI6 | 3.0 |  | 130022 | CDCA5 | -3.1 |
| 3290630 | SERPINA5 | 2.8 |  | 7200601 | MUC1 | -3.0 |
| 6280168 | SERPINA3 | 2.8 |  | 6020735 | GINS2 | -3.0 |
| 6180259 | HSPB8 | 2.6 |  | 5960224 | PTTG3 | -2.9 |
| 1010446 | C1QTNF6 | 2.6 |  | 650347 | NUDT1 | -2.9 |
| 2060291 | STOM | 2.6 |  | 5090754 | KIAA0101 | -2.9 |
| 7610131 | EPAS1 | 2.6 |  | 1470195 | MCM7 | -2.9 |
| 5270619 | FGD3 | 2.5 |  | 5360070 | CCNB2 | -2.8 |
| 6110392 | GNS | 2.4 |  | 4610608 | LOC731049 | -2.8 |
| 4070647 | ALDH3B2 | 2.4 |  | 5420309 | AURKA | -2.7 |
| 3940133 | FAM46A | 2.4 |  | 5900482 | HMGB2 | -2.7 |
| 1230025 | CYB5R1 | 2.4 |  | 2060674 | CA2 | -2.6 |
| 70634 | RABAC1 | 2.4 |  | 4260368 | UBE2C | -2.6 |
| 2710735 | RASD1 | 2.3 |  | 4730196 | TK1 | -2.6 |
| 1260497 | RFTN1 | 2.3 |  | 460358 | CA2 | -2.6 |
| 2100196 | ISG15 | 2.3 |  | 1500010 | CDC20 | -2.5 |
| 5960168 | C10ORF58 | 2.3 |  | 1070215 | CAV1 | -2.5 |
| 1300601 | ST3GAL1 | 2.2 |  | 6960022 | SEP5 | -2.5 |
| 460113 | MAGED1 | 2.2 |  | 5910349 | SPC24 | -2.5 |
| 7650358 | TGFBI | 2.2 |  | 2350685 | HEY2 | -2.5 |
| 7570324 | ID3 | 2.2 |  | 3990619 | TOP2A | -2.5 |
| 6200086 | PSAP | 2.1 |  | 1010470 | C9ORF140 | -2.5 |
| 4890671 | DHRS2 | 2.1 |  | 5310471 | UBE2C | -2.5 |
| 430465 | G6PD | 2.1 |  | 3890475 | CENPN | -2.5 |
| 6480059 | ACTA2 | 2.1 |  | 3120341 | RAMP3 | -2.5 |
| 1980288 | CTSH | 2.1 |  | 5340338 | E2F2 | -2.4 |
| 7650333 | PSAP | 2.1 |  | 5690687 | CTGF | -2.4 |
| 6350632 | TSC22D3 | 2.1 |  | 240086 | PHGDH | -2.4 |
| 1510424 | S100P | 2.1 |  | 1500553 | NUSAP1 | -2.4 |
| 1340039 | TFPI | 2.1 |  | 4210088 | CKS1B | -2.4 |
| 6560156 | DUSP3 | 2.1 |  | 7160239 | FOSB | -2.3 |
| 1110092 | CTSD | 2.0 |  | 2140524 | HIST1H3D | -2.3 |
| 60670 | LXN | 2.0 |  | 3120114 | HIST1H2AM | -2.3 |
| 2570079 | STAT1 | 2.0 |  | 5820754 | HIST1H2AJ | -2.3 |
| 7200041 | PPT1 | 2.0 |  | 780528 | CKS2 | -2.3 |
| 4830424 | MYLIP | 2.0 |  | 4730605 | AURKA | -2.3 |
|  |  |  |  | 2070220 | C21ORF58 | -2.2 |
|  |  |  |  | 2630433 | CDT1 | -2.2 |
|  |  |  |  | 730142 | LOC643287 | -2.2 |
|  |  |  |  | 5220022 | GFRA1 | -2.2 |
|  |  |  |  | 2450358 | CKS1B | -2.2 |
|  |  |  |  | 3130541 | CCNF | -2.2 |
|  |  |  |  | 2340370 | HIST1H2AI | -2.2 |
|  |  |  |  | 6330039 | HMG1L1 | -2.1 |
|  |  |  |  | 2640292 | CTGF | -2.1 |
|  |  |  |  | 150343 | HS.213061 | -2.1 |
|  |  |  |  | 7610537 | HMGB2 | -2.1 |
|  |  |  |  | 5260014 | CDKN3 | -2.1 |
|  |  |  |  | 2030315 | RFC4 | -2.1 |
|  |  |  |  | 7380670 | MYB | -2.1 |
|  |  |  |  | 6510176 | TUBA1B | -2.1 |
|  |  |  |  | 6040347 | RAMP3 | -2.1 |
|  |  |  |  | 4390398 | LCN2 | -2.1 |
|  |  |  |  | 5310044 | NBPF20 | -2.1 |
|  |  |  |  | 6200148 | PCP4 | -2.1 |
|  |  |  |  | 5090095 | KIFC1 | -2.0 |
|  |  |  |  | 2710292 | H2AFZ | -2.0 |
|  |  |  |  | 6370474 | CDCA3 | -2.0 |
|  |  |  |  | 3370703 | FEN1 | -2.0 |
|  |  |  |  | 7380162 | HIST1H2AK | -2.0 |
|  |  |  |  | 2190674 | IGFBP5 | -2.0 |
|  |  |  |  | 1500674 | HIST2H3C | -2.0 |
