# Supplementary material for: TFPI1 Mediates Resistance to Doxorubicin in Breast Cancer Cells by Inducing a Hypoxic-Like Response
Source: PLoS One. 2014 Jan 28;9(1):e84611. doi: 10.1371/journal.pone.0084611 (PMC3904823; doi:10.1371/journal.pone.0084611)
Supplement: Table S2 — Gene expression changes in MCF7 cells comparing parental cells after a 48 hour treatment with 1 µM DOX, and comparing DOX selected cells with cells after the 48 hour treatment. Edges 3, 4, 7 and 8 refer to the numbering system shown in Fig. S1. (DOCX) [file pone.0084611.s009.docx]

**Supplementary Table 2 Gene expression changes in MCF7 cells comparing parental cells after a 48 hour treatment with 1 μM DOX, and comparing DOX selected cells with cells after the 48 hour treatment.** Edges 3, 4, 7 and 8 refer to the numbering system shown in Suppl. Fig. 1.

| **DOX^48^ on MCF7 - UP (550; edge 3)** | | | **DOX^48^ on MCF7 - DOWN (186; edge 4)** | | | **DOX on DOX^48^ - UP (121; edge 8)** | | | **DOX on DOX^48^ - DOWN (498; edge 7)** | | |
| --- | --- | --- | --- | --- | --- | --- | --- | --- | --- | --- | --- |
|  |  |  |  |  |  |  |  |  |  |  |  |
| **Probe ID** | **TargetID** | **FC** | **Probe ID** | **TargetID** | **FC** | **Probe ID** | **TargetID** | **FC** | **Probe ID** | **TargetID** | **FC** |
| 1260020 | TP53I3 | 25.4 | 3170286 | LOC647000 | -6.0 | 450424 | CXCR7 | 6.2 | 50309 | DPYSL4 | -23.5 |
| 50309 | DPYSL4 | 22.8 | 3890349 | HIST1H4C | -5.5 | 3990170 | IFI27 | 5.1 | 3130220 | TMEM158 | -22.6 |
| 4560328 | FSCN1 | 21.8 | 6510176 | TUBA1B | -5.4 | 60670 | LXN | 4.4 | 4560328 | FSCN1 | -19.2 |
| 3130220 | TMEM158 | 21.0 | 4230091 | HNRPA2B1 | -5.0 | 5870474 | RHOBTB3 | 4.3 | 1260020 | TP53I3 | -17.1 |
| 6480059 | ACTA2 | 18.2 | 2510019 | EIF3E | -5.0 | 3170286 | LOC647000 | 4.3 | 6520139 | FGFR3 | -14.8 |
| 7330392 | TAP1 | 15.2 | 450424 | CXCR7 | -4.9 | 6860047 | HS.579631 | 4.2 | 2900274 | VASN | -12.7 |
| 610451 | HIST2H2AA3 | 14.1 | 6650079 | C3ORF14 | -4.6 | 4120553 | WISP2 | 4.2 | 1010333 | MSX1 | -12.3 |
| 2070360 | C17ORF82 | 13.9 | 6860047 | HS.579631 | -4.3 | 610437 | CD24 | 3.8 | 7330392 | TAP1 | -11.0 |
| 1010333 | MSX1 | 12.9 | 2070494 | PRC1 | -4.2 | 3290630 | SERPINA5 | 3.7 | 2070360 | C17ORF82 | -9.9 |
| 4230201 | CDKN1A | 12.6 | 6770438 | HIST1H1B | -4.2 | 4850731 | TFPI | 3.5 | 6480059 | ACTA2 | -9.7 |
| 2900274 | VASN | 12.1 | 2230619 | HIST1H4E | -4.1 | 1340039 | TFPI | 3.4 | 3710040 | SFN | -9.6 |
| 3870678 | HIST1H2AE | 10.0 | 6580577 | HNRNPD | -4.0 | 6180259 | HSPB8 | 3.3 | 610451 | HIST2H2AA3 | -9.0 |
| 1820592 | HIST2H2AA3 | 9.8 | 3190092 | LDHA | -3.9 | 2680128 | TMBIM4 | 3.3 | 3870678 | HIST1H2AE | -8.8 |
| 6520139 | FGFR3 | 9.0 | 270593 | GPER | -3.9 | 2510019 | EIF3E | 3.2 | 4290050 | ACOT7 | -8.7 |
| 270152 | SLC7A5 | 8.8 | 7650026 | MUC1 | -3.5 | 7150634 | APOD | 3.2 | 1510008 | GALR2 | -8.7 |
| 7610615 | SLC6A10P | 8.5 | 5870474 | RHOBTB3 | -3.2 | 6350189 | MGC4677 | 3.2 | 6100022 | HIST2H2AC | -8.3 |
| 1510008 | GALR2 | 8.4 | 4760243 | LOC648210 | -3.2 | 4070647 | ALDH3B2 | 3.1 | 5090750 | FOXC1 | -7.7 |
| 5090671 | GDF15 | 8.2 | 6650053 | MCM3 | -3.2 | 270593 | GPER | 3.1 | 7610615 | SLC6A10P | -7.1 |
| 7550064 | KCTD5 | 7.7 | 520463 | GPER | -3.2 | 4230091 | HNRPA2B1 | 3.1 | 7570484 | TFF3 | -7.0 |
| 3710040 | SFN | 7.6 | 1260162 | DNMT1 | -3.1 | 7570324 | ID3 | 3.0 | 780528 | CKS2 | -6.8 |
| 1510424 | S100P | 7.4 | 520209 | SRP9 | -3.1 | 7400377 | CEACAM6 | 3.0 | 7550064 | KCTD5 | -6.8 |
| 510373 | RHBDD2 | 7.4 | 610437 | CD24 | -3.0 | 3710154 | SLC7A2 | 2.9 | 5310411 | H2AFJ | -6.4 |
| 4260386 | CTSL1 | 7.2 | 1940593 | LOC653226 | -3.0 | 6650079 | C3ORF14 | 2.9 | 1820592 | HIST2H2AA3 | -6.4 |
| 6590592 | SDC1 | 7.1 | 3830131 | TUBA1C | -3.0 | 1010446 | C1QTNF6 | 2.8 | 3170100 | BMP7 | -6.1 |
| 4150189 | CTSL1 | 7.0 | 6280446 | LOC642989 | -3.0 | 4640689 | EIF4A2 | 2.8 | 830278 | GLIPR2 | -6.0 |
| 6650746 | RHBDD2 | 7.0 | 4640689 | EIF4A2 | -3.0 | 4830520 | KRT80 | 2.8 | 610201 | HES6 | -6.0 |
| 2710735 | RASD1 | 6.9 | 4540349 | LSM2 | -2.9 | 650168 | C17ORF58 | 2.8 | 4490475 | SLC30A3 | -5.4 |
| 2600463 | TNFRSF10B | 6.9 | 6960025 | DPY30 | -2.9 | 6580577 | HNRNPD | 2.8 | 5080192 | SERPINE2 | -5.3 |
| 990500 | AVPI1 | 6.8 | 1940164 | LSM5 | -2.9 | 4010347 | COPS5 | 2.7 | 6840075 | NP | -5.3 |
| 5490431 | SAT1 | 6.8 | 6760037 | SYTL2 | -2.8 | 520463 | GPER | 2.7 | 3990598 | DRAP1 | -5.1 |
| 2650521 | LRPAP1 | 6.7 | 5820601 | CCND1 | -2.8 | 5090215 | IFI6 | 2.7 | 6650746 | RHBDD2 | -5.0 |
| 5090750 | FOXC1 | 6.4 | 2710192 | MRPL3 | -2.8 | 1850468 | CCDC56 | 2.7 | 270152 | SLC7A5 | -5.0 |
| 2100196 | ISG15 | 6.4 | 1740136 | SLC38A2 | -2.7 | 1230523 | COMMD3 | 2.7 | 4780128 | ATF3 | -4.9 |
| 6840075 | NP | 6.3 | 6200402 | MT1A | -2.7 | 290603 | AARS | 2.7 | 4560717 | MDK | -4.7 |
| 3170162 | PRNP | 6.2 | 7330612 | ATP5G2 | -2.7 | 630243 | ATAD4 | 2.7 | 6020424 | LMNA | -4.7 |
| 4290050 | ACOT7 | 6.1 | 2680128 | TMBIM4 | -2.7 | 1470706 | C8ORF55 | 2.6 | 510373 | RHBDD2 | -4.7 |
| 10133 | SLC9A1 | 6.1 | 1470195 | MCM7 | -2.7 | 2710192 | MRPL3 | 2.6 | 2600463 | TNFRSF10B | -4.6 |
| 5080192 | SERPINE2 | 5.9 | 630243 | ATAD4 | -2.7 | 7320382 | SLC38A1 | 2.6 | 990500 | AVPI1 | -4.6 |
| 7570673 | UPP1 | 5.8 | 1430735 | NET1 | -2.7 | 4290605 | SLC44A1 | 2.6 | 6590592 | SDC1 | -4.6 |
| 4560717 | MDK | 5.7 | 7320382 | SLC38A1 | -2.7 | 6510176 | TUBA1B | 2.6 | 3290338 | POLR2A | -4.6 |
| 6400195 | MRPS6 | 5.6 | 450615 | MT2A | -2.7 | 6760037 | SYTL2 | 2.6 | 2650521 | LRPAP1 | -4.5 |
| 830278 | GLIPR2 | 5.5 | 6520424 | HIST1H3C | -2.6 | 3310538 | CD36 | 2.6 | 6200468 | CKS2 | -4.5 |
| 3940133 | FAM46A | 5.4 | 7200601 | MUC1 | -2.6 | 2000220 | HEATR6 | 2.6 | 6660132 | DCXR | -4.4 |
| 5860187 | PHYH | 5.4 | 5690066 | XBP1 | -2.6 | 6580131 | MRPL22 | 2.5 | 6650035 | LOC338758 | -4.3 |
| 3130079 | EIF2B2 | 5.3 | 3120341 | RAMP3 | -2.6 | 4860114 | CXCR7 | 2.5 | 3390730 | DOLK | -4.3 |
| 6650035 | LOC338758 | 5.2 | 1170170 | STC2 | -2.6 | 3190092 | LDHA | 2.5 | 4560164 | PDLIM7 | -4.3 |
| 5310411 | H2AFJ | 5.2 | 2190674 | IGFBP5 | -2.6 | 6840577 | KPNB1 | 2.5 | 5490431 | SAT1 | -4.3 |
| 2810367 | ABCB6 | 5.2 | 7380670 | MYB | -2.6 | 4390372 | BNIPL | 2.4 | 4570102 | BRMS1 | -4.3 |
| 4780128 | ATF3 | 5.2 | 2450156 | XBP1 | -2.6 | 1400070 | TBC1D9 | 2.4 | 7570673 | UPP1 | -4.2 |
| 3990598 | DRAP1 | 5.2 | 5340338 | E2F2 | -2.5 | 2120452 | MLPH | 2.4 | 6380717 | HSPA1A | -4.2 |
| 2140735 | RALGDS | 5.2 | 650168 | C17ORF58 | -2.5 | 1010246 | IFI6 | 2.4 | 6400195 | MRPS6 | -4.2 |
| 5420575 | SLC3A2 | 5.1 | 150343 | HS.213061 | -2.5 | 1940593 | LOC653226 | 2.4 | 5490673 | AP1S1 | -4.2 |
| 6560441 | XPC | 5.0 | 3060646 | C14ORF173 | -2.5 | 610324 | DDX1 | 2.4 | 5860187 | PHYH | -4.2 |
| 5870326 | SLC2A8 | 5.0 | 6840577 | KPNB1 | -2.5 | 520209 | SRP9 | 2.4 | 6220086 | DYNLL1 | -4.1 |
| 4280273 | GM2A | 4.9 | 6840056 | MYL6B | -2.5 | 3170519 | HARS2 | 2.4 | 2140735 | RALGDS | -4.1 |
| 5490673 | AP1S1 | 4.8 | 870148 | MSH6 | -2.5 | 7210632 | AKR1C3 | 2.4 | 3140750 | RBM38 | -4.0 |
| 6100022 | HIST2H2AC | 4.7 | 3710711 | STAG3L2 | -2.4 | 2940291 | QPRT | 2.4 | 4250458 | CFL1 | -4.0 |
| 3390730 | DOLK | 4.7 | 4120553 | WISP2 | -2.4 | 6960025 | DPY30 | 2.3 | 3170162 | PRNP | -4.0 |
| 7610131 | EPAS1 | 4.7 | 7000634 | CSE1L | -2.4 | 4900070 | GSTO1 | 2.3 | 5900592 | MGAT1 | -4.0 |
| 610201 | HES6 | 4.6 | 7150433 | TCTEX1D2 | -2.4 | 5090088 | EPRS | 2.3 | 2360753 | TBX2 | -4.0 |
| 1190739 | ITPRIPL2 | 4.6 | 6580474 | TUBB | -2.4 | 3890398 | WBP2 | 2.3 | 1780538 | SLC6A8 | -3.9 |
| 4490475 | SLC30A3 | 4.6 | 4010347 | COPS5 | -2.4 | 1510202 | RWDD1 | 2.3 | 2470689 | SPHK1 | -3.9 |
| 4570102 | BRMS1 | 4.5 | 1940576 | RPS6KB1 | -2.4 | 6840368 | RDH11 | 2.3 | 5870326 | SLC2A8 | -3.9 |
| 5900592 | MGAT1 | 4.5 | 4590356 | HNRNPD | -2.4 | 4810435 | SCARB2 | 2.3 | 2810367 | ABCB6 | -3.9 |
| 7160059 | ATP6V0B | 4.4 | 4480180 | DEK | -2.4 | 150474 | CA12 | 2.3 | 5490356 | NXF1 | -3.9 |
| 4390121 | ADRM1 | 4.3 | 1090239 | SF3B3 | -2.4 | 7330435 | CCDC6 | 2.3 | 1850075 | AGBL5 | -3.9 |
| 2140121 | BASP1 | 4.3 | 290343 | TTC3 | -2.4 | 6130424 | AKR1A1 | 2.2 | 5130162 | AK1 | -3.9 |
| 6220086 | DYNLL1 | 4.3 | 5960021 | HS.57079 | -2.4 | 2190184 | PRSS23 | 2.2 | 1510424 | S100P | -3.8 |
| 6380717 | HSPA1A | 4.3 | 3890475 | CENPN | -2.4 | 5820601 | CCND1 | 2.2 | 1430681 | TLCD1 | -3.8 |
| 3710647 | MXD4 | 4.3 | 5690274 | MCM6 | -2.4 | 7610747 | MRPL24 | 2.2 | 6560441 | XPC | -3.8 |
| 5130162 | AK1 | 4.3 | 5860152 | CD44 | -2.4 | 2510593 | HRASLS3 | 2.2 | 7650047 | SLC29A4 | -3.8 |
| 6660132 | DCXR | 4.3 | 4560600 | C3ORF57 | -2.4 | 6940255 | PLSCR3 | 2.2 | 6250131 | CABYR | -3.8 |
| 4200450 | G6PD | 4.2 | 6580131 | MRPL22 | -2.3 | 6480630 | ATP9A | 2.2 | 2900543 | ENDOG | -3.8 |
| 630706 | BTBD14A | 4.2 | 4200068 | NUP62 | -2.3 | 4760243 | LOC648210 | 2.2 | 1190739 | ITPRIPL2 | -3.7 |
| 5890450 | ATP6V1E1 | 4.2 | 5220767 | FLNB | -2.3 | 5960021 | HS.57079 | 2.2 | 1240221 | POLR2H | -3.7 |
| 6450138 | BCAP31 | 4.2 | 2000220 | HEATR6 | -2.3 | 6370612 | CHURC1 | 2.2 | 6200132 | FZD9 | -3.7 |
| 2470689 | SPHK1 | 4.1 | 3850053 | ANP32B | -2.3 | 4830687 | BCAS3 | 2.2 | 1990546 | KIAA0152 | -3.7 |
| 3170100 | BMP7 | 4.1 | 7150152 | PTPLAD1 | -2.3 | 650605 | LOC388789 | 2.2 | 4850497 | NXF1 | -3.7 |
| 4230520 | DNCL1 | 4.1 | 4210050 | WDR54 | -2.3 | 7330612 | ATP5G2 | 2.2 | 5910364 | TYMS | -3.7 |
| 6180446 | PRDX1 | 4.1 | 3450632 | C6ORF141 | -2.3 | 3390544 | HSP90AA1 | 2.2 | 4210692 | SAC3D1 | -3.7 |
| 4250458 | CFL1 | 4.1 | 2940044 | TMEM14B | -2.3 | 3460739 | RAB17 | 2.2 | 7200242 | SULF2 | -3.7 |
| 270129 | TMEM8 | 4.0 | 1850468 | CCDC56 | -2.3 | 4540349 | LSM2 | 2.2 | 7550070 | LOC730316 | -3.7 |
| 3890521 | DEDD2 | 4.0 | 1510202 | RWDD1 | -2.3 | 3890228 | MPZL2 | 2.2 | 4390121 | ADRM1 | -3.7 |
| 7380706 | NINJ1 | 4.0 | 5900746 | GNL3L | -2.3 | 4890671 | DHRS2 | 2.2 | 20446 | CEBPB | -3.7 |
| 4810128 | PHLDA2 | 4.0 | 6280672 | TMEM49 | -2.3 | 7150433 | TCTEX1D2 | 2.1 | 1500192 | HIST3H2A | -3.7 |
| 6860543 | ATP6V1E1 | 4.0 | 1010048 | CXXC5 | -2.3 | 5090347 | CD24 | 2.1 | 4230520 | DNCL1 | -3.6 |
| 1430681 | TLCD1 | 4.0 | 3360059 | CASP2 | -2.3 | 1260497 | RFTN1 | 2.1 | 6420369 | MDH2 | -3.6 |
| 1780538 | SLC6A8 | 4.0 | 5560246 | TPM1 | -2.3 | 6130138 | POLR3C | 2.1 | 940750 | HS.553217 | -3.6 |
| 4230678 | HIST2H2BE | 4.0 | 7400358 | TINP1 | -2.3 | 5260044 | GMFB | 2.1 | 5690711 | TMEM134 | -3.6 |
| 1990520 | GPS1 | 4.0 | 4900070 | GSTO1 | -2.3 | 6250382 | ANAPC13 | 2.1 | 6660315 | INPP1 | -3.6 |
| 5390411 | PHPT1 | 3.9 | 5340187 | TUBA1A | -2.3 | 7150152 | PTPLAD1 | 2.1 | 3310477 | TPI1 | -3.6 |
| 2060121 | FUCA1 | 3.9 | 3710154 | SLC7A2 | -2.3 | 5690066 | XBP1 | 2.1 | 4210095 | ATP1A1 | -3.5 |
| 7650047 | SLC29A4 | 3.9 | 2940079 | ENY2 | -2.3 | 3170273 | FER1L3 | 2.1 | 610356 | ZDHHC9 | -3.5 |
| 780168 | ISCU | 3.9 | 7320750 | ILVBL | -2.3 | 2940044 | TMEM14B | 2.1 | 670671 | TMEM134 | -3.5 |
| 610356 | ZDHHC9 | 3.9 | 60670 | LXN | -2.2 | 1990253 | TDG | 2.1 | 3710647 | MXD4 | -3.5 |
| 6250280 | PRDX1 | 3.9 | 5810605 | CCNI | -2.2 | 1010048 | CXXC5 | 2.1 | 6280504 | LOC100008589 | -3.5 |
| 20446 | CEBPB | 3.9 | 3420543 | PSMA4 | -2.2 | 50195 | RAB25 | 2.1 | 4490259 | COX8A | -3.5 |
| 4890181 | RAP1GAP | 3.9 | 3780528 | RPS27A | -2.2 | 2940079 | ENY2 | 2.1 | 6560390 | CRELD2 | -3.4 |
| 3140750 | RBM38 | 3.8 | 4780050 | UGDH | -2.2 | 5270619 | FGD3 | 2.1 | 4880433 | IDH2 | -3.4 |
| 6020564 | RRAGA | 3.8 | 5090088 | EPRS | -2.2 | 3450072 | CLIC1 | 2.1 | 990176 | RN7SK | -3.4 |
| 2060291 | STOM | 3.8 | 7150132 | PNN | -2.2 | 3120367 | SQLE | 2.1 | 5560075 | MFGE8 | -3.4 |
| 5490356 | NXF1 | 3.8 | 6040347 | RAMP3 | -2.2 | 7560025 | PSMA1 | 2.1 | 4810128 | PHLDA2 | -3.4 |
| 1500619 | LOC642755 | 3.8 | 2630161 | NOL11 | -2.2 | 6270138 | TACSTD2 | 2.1 | 10133 | SLC9A1 | -3.4 |
| 2470386 | DEXI | 3.8 | 3170184 | RPL36AL | -2.2 | 2340372 | MRFAP1 | 2.1 | 2630768 | LMNA | -3.4 |
| 670671 | TMEM134 | 3.8 | 510341 | VEZF1 | -2.2 | 830735 | CENTA1 | 2.1 | 1780619 | EHD1 | -3.3 |
| 5560131 | ATOX1 | 3.8 | 6180066 | NACA | -2.2 | 1740136 | SLC38A2 | 2.1 | 1820594 | HBEGF | -3.3 |
| 2900543 | ENDOG | 3.7 | 2680450 | HIST1H1D | -2.2 | 7150196 | TMEM87A | 2.1 | 2690047 | ARL6IP1 | -3.3 |
| 1230672 | TFF1 | 3.7 | 650605 | LOC388789 | -2.2 | 4560367 | NDUFA8 | 2.1 | 3610646 | LOC401019 | -3.3 |
| 4850497 | NXF1 | 3.7 | 3610064 | NACA | -2.2 | 5900746 | GNL3L | 2.1 | 4670021 | NPEPL1 | -3.3 |
| 1850075 | AGBL5 | 3.7 | 3840167 | TMEM14C | -2.2 | 4560022 | C2ORF25 | 2.0 | 110661 | TSPAN4 | -3.3 |
| 7570243 | SSR4 | 3.7 | 4850731 | TFPI | -2.2 | 2900441 | ZBTB4 | 2.0 | 2260619 | MIB2 | -3.3 |
| 6560390 | CRELD2 | 3.7 | 4150128 | LOC340598 | -2.2 | 6110754 | ATP5O | 2.0 | 6450605 | DDOST | -3.2 |
| 1990546 | KIAA0152 | 3.7 | 2120452 | MLPH | -2.2 | 3420543 | PSMA4 | 2.0 | 1990520 | GPS1 | -3.2 |
| 3400438 | HLA-A | 3.7 | 290603 | AARS | -2.2 | 6860300 | SMARCA4 | 2.0 | 650634 | SLC25A39 | -3.2 |
| 4200692 | NEU1 | 3.7 | 1230044 | SSBP1 | -2.2 | 4280672 | KRT86 | 2.0 | 60121 | CTSB | -3.2 |
| 7570484 | TFF3 | 3.7 | 6860300 | SMARCA4 | -2.2 | 3060110 | SFRS6 | 2.0 | 4280273 | GM2A | -3.2 |
| 6660315 | INPP1 | 3.6 | 6550754 | EVL | -2.2 | 1770546 | ATIC | 2.0 | 2470386 | DEXI | -3.2 |
| 6200132 | FZD9 | 3.6 | 1470706 | C8ORF55 | -2.2 | 1260162 | DNMT1 | 2.0 | 2710735 | RASD1 | -3.2 |
| 1430647 | TAX1BP3 | 3.6 | 2120524 | IGFBP5 | -2.2 | 2490333 | ZNF467 | 2.0 | 3890521 | DEDD2 | -3.2 |
| 2320689 | LOC653610 | 3.6 | 2340372 | MRFAP1 | -2.2 | 7100220 | XYLT2 | 2.0 | 1510291 | PTTG1 | -3.2 |
| 1510703 | MFSD3 | 3.6 | 940075 | SPDEF | -2.2 | 7380110 | CDK4 | 2.0 | 7550343 | PRDX6 | -3.2 |
| 6020424 | LMNA | 3.5 | 1070215 | CAV1 | -2.2 |  |  |  | 7380706 | NINJ1 | -3.2 |
| 6110630 | HIST1H2BK | 3.5 | 6270138 | TACSTD2 | -2.2 |  |  |  | 1940360 | TPI1 | -3.1 |
| 4730685 | RAB7A | 3.5 | 7150349 | TPD52L1 | -2.2 |  |  |  | 5960682 | RBPMS2 | -3.1 |
| 5560075 | MFGE8 | 3.5 | 6560672 | HNRPR | -2.2 |  |  |  | 870537 | LOC134997 | -3.1 |
| 430465 | G6PD | 3.5 | 4250445 | RPL4 | -2.2 |  |  |  | 780341 | CCDC3 | -3.1 |
| 1780619 | EHD1 | 3.5 | 4180050 | TIMELESS | -2.2 |  |  |  | 3420523 | RHBDF2 | -3.1 |
| 1500192 | HIST3H2A | 3.5 | 5360070 | CCNB2 | -2.1 |  |  |  | 3370164 | ATP1A1 | -3.1 |
| 940750 | HS.553217 | 3.5 | 6560088 | C3ORF14 | -2.1 |  |  |  | 1940129 | SIRPA | -3.1 |
| 3120544 | METRN | 3.5 | 7160753 | SAE1 | -2.1 |  |  |  | 3130079 | EIF2B2 | -3.1 |
| 7200242 | SULF2 | 3.4 | 4560022 | C2ORF25 | -2.1 |  |  |  | 3800050 | ADCY3 | -3.1 |
| 3290338 | POLR2A | 3.4 | 2760292 | PPP1CC | -2.1 |  |  |  | 1110338 | MYL6 | -3.1 |
| 3940482 | CDR2L | 3.4 | 6110754 | ATP5O | -2.1 |  |  |  | 6020564 | RRAGA | -3.1 |
| 1260086 | ID2 | 3.4 | 4880360 | FBL | -2.1 |  |  |  | 6450138 | BCAP31 | -3.1 |
| 6250131 | CABYR | 3.4 | 5220022 | GFRA1 | -2.1 |  |  |  | 1500619 | LOC642755 | -3.1 |
| 1820594 | HBEGF | 3.4 | 3800647 | UGCG | -2.1 |  |  |  | 3940482 | CDR2L | -3.0 |
| 3420523 | RHBDF2 | 3.3 | 5090754 | KIAA0101 | -2.1 |  |  |  | 4070017 | ODC1 | -3.0 |
| 7570494 | CNFN | 3.3 | 620754 | RPS5 | -2.1 |  |  |  | 7570494 | CNFN | -3.0 |
| 4900440 | CIB1 | 3.3 | 6420424 | PAICS | -2.1 |  |  |  | 5090156 | NBL1 | -3.0 |
| 7150475 | WDR1 | 3.3 | 6020735 | GINS2 | -2.1 |  |  |  | 630706 | BTBD14A | -3.0 |
| 7550343 | PRDX6 | 3.3 | 2850575 | RRM1 | -2.1 |  |  |  | 5560494 | RPS15 | -3.0 |
| 4830056 | ARPC5L | 3.3 | 6400270 | HPRT1 | -2.1 |  |  |  | 6450543 | CST3 | -3.0 |
| 1240221 | POLR2H | 3.3 | 1450390 | RPL17 | -2.1 |  |  |  | 1260086 | ID2 | -3.0 |
| 990176 | RN7SK | 3.3 | 7380110 | CDK4 | -2.1 |  |  |  | 840296 | LOC440926 | -3.0 |
| 4260044 | SQSTM1 | 3.2 | 3990619 | TOP2A | -2.1 |  |  |  | 6770309 | MYL6 | -3.0 |
| 4920767 | FTL | 3.2 | 3060110 | SFRS6 | -2.1 |  |  |  | 2600286 | CCS | -3.0 |
| 4210095 | ATP1A1 | 3.2 | 3890255 | TOP2B | -2.1 |  |  |  | 5090021 | HS.535392 | -3.0 |
| 4290072 | SERTAD1 | 3.2 | 1400070 | TBC1D9 | -2.1 |  |  |  | 2850180 | NUDT16L1 | -3.0 |
| 3870630 | ATP6V1F | 3.2 | 4220707 | PMPCB | -2.1 |  |  |  | 3390551 | C14ORF78 | -2.9 |
| 4150687 | NDUFB2 | 3.2 | 4390546 | TINP1 | -2.1 |  |  |  | 1430280 | CEBPA | -2.9 |
| 650634 | SLC25A39 | 3.2 | 6270600 | TMEM64 | -2.1 |  |  |  | 780168 | ISCU | -2.9 |
| 3710609 | TSPAN17 | 3.2 | 5130674 | CSE1L | -2.1 |  |  |  | 4900440 | CIB1 | -2.9 |
| 1780678 | LOC374395 | 3.1 | 4560056 | NONO | -2.1 |  |  |  | 4180079 | CRISPLD2 | -2.9 |
| 780528 | CKS2 | 3.1 | 610324 | DDX1 | -2.1 |  |  |  | 1470608 | ROM1 | -2.9 |
| 2260619 | MIB2 | 3.1 | 780358 | RAB11A | -2.1 |  |  |  | 2850026 | H3F3A | -2.9 |
| 60121 | CTSB | 3.1 | 130022 | CDCA5 | -2.1 |  |  |  | 990161 | CDIPT | -2.9 |
| 6450605 | DDOST | 3.1 | 3610286 | GLTSCR2 | -2.1 |  |  |  | 5870678 | LOC441763 | -2.9 |
| 4280136 | ADRM1 | 3.1 | 3390544 | HSP90AA1 | -2.1 |  |  |  | 4730025 | LOC341457 | -2.9 |
| 670528 | GYG1 | 3.1 | 7380689 | RPL22 | -2.1 |  |  |  | 580132 | LAPTM4B | -2.9 |
| 1030747 | HLA-E | 3.1 | 1660528 | CKLF | -2.1 |  |  |  | 2100196 | ISG15 | -2.9 |
| 1470259 | EXOC7 | 3.1 | 2030315 | RFC4 | -2.1 |  |  |  | 1470086 | RPS19 | -2.8 |
| 2600286 | CCS | 3.1 | 3610259 | TUBB | -2.1 |  |  |  | 5420575 | SLC3A2 | -2.8 |
| 620615 | NDUFA7 | 3.1 | 3780056 | GGCT | -2.1 |  |  |  | 6110630 | HIST1H2BK | -2.8 |
| 3710324 | SURF1 | 3.1 | 3420451 | C15ORF15 | -2.1 |  |  |  | 2060121 | FUCA1 | -2.8 |
| 990161 | CDIPT | 3.1 | 4860692 | SHFM1 | -2.1 |  |  |  | 4850097 | RPLP2 | -2.8 |
| 770541 | EI24 | 3.1 | 4860114 | CXCR7 | -2.1 |  |  |  | 2640719 | RN7SK | -2.8 |
| 4860286 | UBB | 3.1 | 7040184 | CCDC34 | -2.1 |  |  |  | 4860286 | UBB | -2.8 |
| 620433 | C1ORF122 | 3.1 | 6180537 | RBMX | -2.0 |  |  |  | 630259 | VPS37D | -2.8 |
| 1430280 | CEBPA | 3.1 | 4880129 | TOMM7 | -2.0 |  |  |  | 4730685 | RAB7A | -2.8 |
| 4390703 | PTPN1 | 3.0 | 2680471 | ESD | -2.0 |  |  |  | 7570243 | SSR4 | -2.8 |
| 2360020 | FADS1 | 3.0 | 5340129 | AKR1C2 | -2.0 |  |  |  | 4760338 | CDC25B | -2.8 |
| 4480288 | ISG20L1 | 3.0 | 6940377 | TOMM7 | -2.0 |  |  |  | 3710324 | SURF1 | -2.8 |
| 4180079 | CRISPLD2 | 3.0 | 610519 | TPM1 | -2.0 |  |  |  | 4880477 | LOC643031 | -2.8 |
| 4590494 | YIF1A | 3.0 | 6130193 | TMEM109 | -2.0 |  |  |  | 4220519 | UBA52 | -2.8 |
| 5810328 | FTL | 3.0 | 7040095 | RPL17 | -2.0 |  |  |  | 2060440 | MAFB | -2.8 |
| 780341 | CCDC3 | 3.0 | 130519 | STAT2 | -2.0 |  |  |  | 7160239 | FOSB | -2.8 |
| 4010064 | P4HA2 | 3.0 | 3450072 | CLIC1 | -2.0 |  |  |  | 5390161 | DUSP5 | -2.8 |
| 3610397 | ATP6V0C | 3.0 | 1850259 | NFIC | -2.0 |  |  |  | 5670594 | NMB | -2.8 |
| 4070017 | ODC1 | 3.0 | 2650553 | TPD52L1 | -2.0 |  |  |  | 4150687 | NDUFB2 | -2.8 |
| 7550070 | LOC730316 | 3.0 | 4920537 | POLA2 | -2.0 |  |  |  | 1510703 | MFSD3 | -2.8 |
| 2900594 | PGD | 3.0 | 4290605 | SLC44A1 | -2.0 |  |  |  | 5310494 | LOC440733 | -2.8 |
| 7040670 | DHRS3 | 3.0 | 1980246 | MYO5C | -2.0 |  |  |  | 3710609 | TSPAN17 | -2.8 |
| 2320367 | NDUFA13 | 3.0 | 2850482 | ATP6V1B1 | -2.0 |  |  |  | 5490603 | LOC401019 | -2.8 |
| 6450543 | CST3 | 3.0 | 1440300 | SLC27A3 | -2.0 |  |  |  | 270129 | TMEM8 | -2.8 |
| 4880673 | GADD45A | 2.9 | 7610747 | MRPL24 | -2.0 |  |  |  | 2030553 | H3F3A | -2.8 |
| 580132 | LAPTM4B | 2.9 | 6580270 | LOC646723 | -2.0 |  |  |  | 3610397 | ATP6V0C | -2.8 |
| 6420369 | MDH2 | 2.9 |  |  |  |  |  |  | 1990196 | DACT3 | -2.7 |
| 1940129 | SIRPA | 2.9 |  |  |  |  |  |  | 6510519 | IL17D | -2.7 |
| 3310477 | TPI1 | 2.9 |  |  |  |  |  |  | 5270110 | EIF4A3 | -2.7 |
| 6650348 | LAPTM4B | 2.9 |  |  |  |  |  |  | 3450156 | CSNK2B | -2.7 |
| 5960682 | RBPMS2 | 2.9 |  |  |  |  |  |  | 60397 | TRK1 | -2.7 |
| 5670594 | NMB | 2.9 |  |  |  |  |  |  | 3310301 | LOC646531 | -2.7 |
| 2850180 | NUDT16L1 | 2.9 |  |  |  |  |  |  | 6580639 | ACOT7 | -2.7 |
| 5090053 | FDX1L | 2.9 |  |  |  |  |  |  | 1690360 | KREMEN2 | -2.7 |
| 1240750 | SNTB2 | 2.9 |  |  |  |  |  |  | 1230672 | TFF1 | -2.7 |
| 7160246 | LOC339123 | 2.9 |  |  |  |  |  |  | 6940242 | BRPF1 | -2.7 |
| 4250291 | CHPF | 2.9 |  |  |  |  |  |  | 4290072 | SERTAD1 | -2.7 |
| 6420541 | UBL5 | 2.9 |  |  |  |  |  |  | 70767 | BAIAP2 | -2.7 |
| 1470608 | ROM1 | 2.9 |  |  |  |  |  |  | 7160246 | LOC339123 | -2.7 |
| 6330377 | ATP6V0D1 | 2.8 |  |  |  |  |  |  | 5560131 | ATOX1 | -2.7 |
| 2360753 | TBX2 | 2.8 |  |  |  |  |  |  | 7150475 | WDR1 | -2.7 |
| 5390161 | DUSP5 | 2.8 |  |  |  |  |  |  | 380050 | RHBDF2 | -2.7 |
| 6200468 | CKS2 | 2.8 |  |  |  |  |  |  | 4390703 | PTPN1 | -2.7 |
| 4570091 | C3ORF60 | 2.8 |  |  |  |  |  |  | 7400050 | CCM2 | -2.7 |
| 4150309 | ZNHIT1 | 2.8 |  |  |  |  |  |  | 6400138 | PRMT1 | -2.7 |
| 2490754 | MAP1LC3B | 2.8 |  |  |  |  |  |  | 6980685 | LOC730740 | -2.7 |
| 4900333 | HAGH | 2.8 |  |  |  |  |  |  | 1430647 | TAX1BP3 | -2.7 |
| 4210692 | SAC3D1 | 2.8 |  |  |  |  |  |  | 2360020 | FADS1 | -2.7 |
| 1300671 | NCOA4 | 2.8 |  |  |  |  |  |  | 3180541 | LOC440093 | -2.7 |
| 520184 | HPCAL1 | 2.8 |  |  |  |  |  |  | 770541 | EI24 | -2.7 |
| 4210041 | UQCRQ | 2.8 |  |  |  |  |  |  | 6650348 | LAPTM4B | -2.7 |
| 7650477 | STUB1 | 2.8 |  |  |  |  |  |  | 6380220 | MAGMAS | -2.7 |
| 5670661 | MGC71993 | 2.8 |  |  |  |  |  |  | 6180446 | PRDX1 | -2.7 |
| 3520753 | ACAA1 | 2.8 |  |  |  |  |  |  | 6860543 | ATP6V1E1 | -2.6 |
| 5090021 | HS.535392 | 2.8 |  |  |  |  |  |  | 4830056 | ARPC5L | -2.6 |
| 6370538 | WBSCR22 | 2.8 |  |  |  |  |  |  | 5390411 | PHPT1 | -2.6 |
| 5910364 | TYMS | 2.8 |  |  |  |  |  |  | 6580753 | MIB2 | -2.6 |
| 4560164 | PDLIM7 | 2.8 |  |  |  |  |  |  | 7510634 | GPRC5C | -2.6 |
| 4670021 | NPEPL1 | 2.8 |  |  |  |  |  |  | 4850487 | UFSP1 | -2.6 |
| 2370128 | DIRC2 | 2.8 |  |  |  |  |  |  | 2070491 | HS.565887 | -2.6 |
| 60397 | TRK1 | 2.7 |  |  |  |  |  |  | 4890181 | RAP1GAP | -2.6 |
| 1850370 | MRPS12 | 2.7 |  |  |  |  |  |  | 3460477 | H3F3A | -2.6 |
| 380050 | RHBDF2 | 2.7 |  |  |  |  |  |  | 5690554 | PPM1D | -2.6 |
| 2070491 | HS.565887 | 2.7 |  |  |  |  |  |  | 3370575 | NADSYN1 | -2.6 |
| 6100075 | GNAS | 2.7 |  |  |  |  |  |  | 2320689 | LOC653610 | -2.6 |
| 270408 | P4HA2 | 2.7 |  |  |  |  |  |  | 5890450 | ATP6V1E1 | -2.6 |
| 770561 | C20ORF108 | 2.7 |  |  |  |  |  |  | 4920110 | GADD45B | -2.6 |
| 3520092 | BAX | 2.7 |  |  |  |  |  |  | 20022 | SRF | -2.6 |
| 4780040 | MRPL41 | 2.7 |  |  |  |  |  |  | 4280136 | ADRM1 | -2.6 |
| 4200259 | ACLY | 2.7 |  |  |  |  |  |  | 4780707 | OTUB1 | -2.6 |
| 70767 | BAIAP2 | 2.7 |  |  |  |  |  |  | 1010647 | TP53I13 | -2.6 |
| 620300 | LASP1 | 2.7 |  |  |  |  |  |  | 10487 | SLC25A25 | -2.6 |
| 2630056 | PRDX5 | 2.7 |  |  |  |  |  |  | 1030747 | HLA-E | -2.6 |
| 1580093 | SCYL1 | 2.7 |  |  |  |  |  |  | 4260386 | CTSL1 | -2.6 |
| 2710672 | KIAA2013 | 2.7 |  |  |  |  |  |  | 2370341 | LOC91561 | -2.5 |
| 4850487 | UFSP1 | 2.7 |  |  |  |  |  |  | 1440296 | H3F3B | -2.5 |
| 5420367 | SOD1 | 2.7 |  |  |  |  |  |  | 4230201 | CDKN1A | -2.5 |
| 5810201 | ZNF593 | 2.7 |  |  |  |  |  |  | 6770025 | FAU | -2.5 |
| 870537 | LOC134997 | 2.7 |  |  |  |  |  |  | 6370661 | AP2S1 | -2.5 |
| 3460386 | B4GALT1 | 2.7 |  |  |  |  |  |  | 4230678 | HIST2H2BE | -2.5 |
| 3370164 | ATP1A1 | 2.7 |  |  |  |  |  |  | 2900594 | PGD | -2.5 |
| 3830653 | DNAJB2 | 2.7 |  |  |  |  |  |  | 7510608 | C12ORF45 | -2.5 |
| 6200086 | PSAP | 2.7 |  |  |  |  |  |  | 6420541 | UBL5 | -2.5 |
| 5860608 | LOC400948 | 2.7 |  |  |  |  |  |  | 6100075 | GNAS | -2.5 |
| 1990196 | DACT3 | 2.7 |  |  |  |  |  |  | 1780678 | LOC374395 | -2.5 |
| 5570279 | HIST1H1C | 2.7 |  |  |  |  |  |  | 5090053 | FDX1L | -2.5 |
| 110661 | TSPAN4 | 2.7 |  |  |  |  |  |  | 2030678 | HIST2H2AB | -2.5 |
| 4900431 | STUB1 | 2.7 |  |  |  |  |  |  | 6590593 | ATP5J2 | -2.5 |
| 4150670 | CDC37 | 2.6 |  |  |  |  |  |  | 7650477 | STUB1 | -2.5 |
| 6450056 | MCOLN1 | 2.6 |  |  |  |  |  |  | 2480288 | GPAA1 | -2.5 |
| 2230187 | MAPBPIP | 2.6 |  |  |  |  |  |  | 1660296 | ID2 | -2.5 |
| 5820255 | HSPC171 | 2.6 |  |  |  |  |  |  | 6770630 | DHDH | -2.5 |
| 6590463 | UBB | 2.6 |  |  |  |  |  |  | 4200259 | ACLY | -2.5 |
| 6380220 | MAGMAS | 2.6 |  |  |  |  |  |  | 4730356 | C19ORF31 | -2.5 |
| 5690554 | PPM1D | 2.6 |  |  |  |  |  |  | 6900079 | PCNA | -2.5 |
| 2060440 | MAFB | 2.6 |  |  |  |  |  |  | 4150670 | CDC37 | -2.5 |
| 2320129 | CSDA | 2.6 |  |  |  |  |  |  | 6860753 | TSPO | -2.5 |
| 630259 | VPS37D | 2.6 |  |  |  |  |  |  | 1010195 | DBI | -2.5 |
| 3450156 | CSNK2B | 2.6 |  |  |  |  |  |  | 620615 | NDUFA7 | -2.5 |
| 4280471 | GUK1 | 2.6 |  |  |  |  |  |  | 5570494 | MRPL33 | -2.5 |
| 7650333 | PSAP | 2.6 |  |  |  |  |  |  | 3120544 | METRN | -2.5 |
| 6510519 | IL17D | 2.6 |  |  |  |  |  |  | 5810088 | AURKAIP1 | -2.5 |
| 1430477 | CTSL2 | 2.6 |  |  |  |  |  |  | 7550470 | KIAA0101 | -2.5 |
| 5570494 | MRPL33 | 2.6 |  |  |  |  |  |  | 60138 | CTH | -2.5 |
| 1780661 | MAN1B1 | 2.6 |  |  |  |  |  |  | 3400438 | HLA-A | -2.5 |
| 6580753 | MIB2 | 2.6 |  |  |  |  |  |  | 3460386 | B4GALT1 | -2.5 |
| 7510634 | GPRC5C | 2.6 |  |  |  |  |  |  | 1770520 | CYC1 | -2.4 |
| 5670400 | PEPD | 2.6 |  |  |  |  |  |  | 4570482 | RPS10 | -2.4 |
| 2470367 | INPPL1 | 2.6 |  |  |  |  |  |  | 2340452 | RN7SL1 | -2.4 |
| 3180053 | FAM129B | 2.6 |  |  |  |  |  |  | 1510468 | GRPEL1 | -2.4 |
| 7510608 | C12ORF45 | 2.6 |  |  |  |  |  |  | 3440452 | ADAM15 | -2.4 |
| 2710161 | SELK | 2.6 |  |  |  |  |  |  | 2640091 | GAPDH | -2.4 |
| 1660296 | ID2 | 2.6 |  |  |  |  |  |  | 4570091 | C3ORF60 | -2.4 |
| 4490017 | GLTP | 2.6 |  |  |  |  |  |  | 7160059 | ATP6V0B | -2.4 |
| 6330132 | ISG20 | 2.6 |  |  |  |  |  |  | 2320129 | CSDA | -2.4 |
| 3390551 | C14ORF78 | 2.5 |  |  |  |  |  |  | 5090561 | ATP5EP2 | -2.4 |
| 1940360 | TPI1 | 2.5 |  |  |  |  |  |  | 5080167 | NOTCH1 | -2.4 |
| 4780707 | OTUB1 | 2.5 |  |  |  |  |  |  | 2320367 | NDUFA13 | -2.4 |
| 6380370 | CCND3 | 2.5 |  |  |  |  |  |  | 1470259 | EXOC7 | -2.4 |
| 5550136 | SMAP2 | 2.5 |  |  |  |  |  |  | 4150189 | CTSL1 | -2.4 |
| 5310170 | CORO1B | 2.5 |  |  |  |  |  |  | 2230626 | MRPL33 | -2.4 |
| 4860315 | TESK1 | 2.5 |  |  |  |  |  |  | 1740576 | LMF2 | -2.4 |
| 1510088 | ATP1B3 | 2.5 |  |  |  |  |  |  | 1230164 | HS.534061 | -2.4 |
| 6400138 | PRMT1 | 2.5 |  |  |  |  |  |  | 6590253 | ALDOA | -2.4 |
| 7160327 | ARPC2 | 2.5 |  |  |  |  |  |  | 5220438 | C20ORF52 | -2.4 |
| 6590593 | ATP5J2 | 2.5 |  |  |  |  |  |  | 3870630 | ATP6V1F | -2.4 |
| 1770520 | CYC1 | 2.5 |  |  |  |  |  |  | 5050681 | TESC | -2.4 |
| 5270110 | EIF4A3 | 2.5 |  |  |  |  |  |  | 4060041 | MYST1 | -2.4 |
| 2650019 | FAM53C | 2.5 |  |  |  |  |  |  | 5340154 | LOC643509 | -2.4 |
| 5900445 | ARFGAP1 | 2.5 |  |  |  |  |  |  | 4480220 | FGFRL1 | -2.4 |
| 3460278 | C22ORF13 | 2.5 |  |  |  |  |  |  | 2360491 | NARF | -2.4 |
| 4490528 | CKAP4 | 2.5 |  |  |  |  |  |  | 3800035 | GLS2 | -2.4 |
| 2480288 | GPAA1 | 2.5 |  |  |  |  |  |  | 3140019 | LOC645317 | -2.4 |
| 6580639 | ACOT7 | 2.5 |  |  |  |  |  |  | 2710161 | SELK | -2.4 |
| 1470427 | ALDH4A1 | 2.5 |  |  |  |  |  |  | 4200450 | G6PD | -2.4 |
| 2000445 | PSMB10 | 2.5 |  |  |  |  |  |  | 4280307 | LAIR1 | -2.3 |
| 4490259 | COX8A | 2.5 |  |  |  |  |  |  | 3390022 | COX4I1 | -2.3 |
| 4730025 | LOC341457 | 2.5 |  |  |  |  |  |  | 5570152 | ATP1A1 | -2.3 |
| 2810082 | C20ORF111 | 2.5 |  |  |  |  |  |  | 7100010 | NBL1 | -2.3 |
| 7040079 | AP1S1 | 2.5 |  |  |  |  |  |  | 6520128 | GPX4 | -2.3 |
| 1030458 | C19ORF10 | 2.5 |  |  |  |  |  |  | 1580093 | SCYL1 | -2.3 |
| 5220398 | PH-4 | 2.5 |  |  |  |  |  |  | 3940133 | FAM46A | -2.3 |
| 1510564 | TMEM4 | 2.5 |  |  |  |  |  |  | 2680484 | RPL41 | -2.3 |
| 1770433 | YWHAG | 2.5 |  |  |  |  |  |  | 870338 | EGR1 | -2.3 |
| 6770630 | DHDH | 2.5 |  |  |  |  |  |  | 7650152 | GNB2L1 | -2.3 |
| 6130725 | ARHGEF18 | 2.5 |  |  |  |  |  |  | 2650019 | FAM53C | -2.3 |
| 4670343 | JAG2 | 2.4 |  |  |  |  |  |  | 1230600 | LOC651064 | -2.3 |
| 3170093 | KIAA0247 | 2.4 |  |  |  |  |  |  | 5890605 | FAM109A | -2.3 |
| 1510468 | GRPEL1 | 2.4 |  |  |  |  |  |  | 2640048 | GAPDH | -2.3 |
| 4230554 | REXO2 | 2.4 |  |  |  |  |  |  | 5870328 | LOC440589 | -2.3 |
| 4220519 | UBA52 | 2.4 |  |  |  |  |  |  | 2760735 | SYNM | -2.3 |
| 3890408 | RRBP1 | 2.4 |  |  |  |  |  |  | 2230037 | NAG18 | -2.3 |
| 5310707 | EI24 | 2.4 |  |  |  |  |  |  | 4150309 | ZNHIT1 | -2.3 |
| 770168 | CLPTM1 | 2.4 |  |  |  |  |  |  | 5900682 | SUMO3 | -2.3 |
| 3800035 | GLS2 | 2.4 |  |  |  |  |  |  | 430369 | TEX264 | -2.3 |
| 3180600 | PNPO | 2.4 |  |  |  |  |  |  | 6550139 | LOC441034 | -2.3 |
| 1980360 | SDF4 | 2.4 |  |  |  |  |  |  | 7100239 | OAZ1 | -2.3 |
| 1010647 | TP53I13 | 2.4 |  |  |  |  |  |  | 6180681 | AKR7A2 | -2.3 |
| 3140193 | NELF | 2.4 |  |  |  |  |  |  | 4490594 | LOC399900 | -2.3 |
| 520358 | IKBKG | 2.4 |  |  |  |  |  |  | 5550136 | SMAP2 | -2.3 |
| 5860242 | LOC642755 | 2.4 |  |  |  |  |  |  | 1070731 | LOC440567 | -2.3 |
| 4010433 | LOC650832 | 2.4 |  |  |  |  |  |  | 2470367 | INPPL1 | -2.3 |
| 1110338 | MYL6 | 2.4 |  |  |  |  |  |  | 6510487 | FAM96B | -2.3 |
| 6330474 | CD151 | 2.4 |  |  |  |  |  |  | 5700370 | POLDIP3 | -2.3 |
| 5220438 | C20ORF52 | 2.4 |  |  |  |  |  |  | 5090068 | STX5 | -2.3 |
| 2230626 | MRPL33 | 2.4 |  |  |  |  |  |  | 5670465 | ADM | -2.3 |
| 160170 | PKM2 | 2.4 |  |  |  |  |  |  | 7040079 | AP1S1 | -2.3 |
| 1440296 | H3F3B | 2.4 |  |  |  |  |  |  | 2760753 | MSH3 | -2.3 |
| 1260360 | LOC729776 | 2.4 |  |  |  |  |  |  | 2370128 | DIRC2 | -2.3 |
| 3460181 | MTCH1 | 2.4 |  |  |  |  |  |  | 770561 | C20ORF108 | -2.3 |
| 4760338 | CDC25B | 2.4 |  |  |  |  |  |  | 4860719 | ROCK2 | -2.3 |
| 3370575 | NADSYN1 | 2.4 |  |  |  |  |  |  | 3930326 | LOC728014 | -2.3 |
| 4250327 | RHOC | 2.4 |  |  |  |  |  |  | 1850370 | MRPS12 | -2.3 |
| 6620437 | HOXA5 | 2.4 |  |  |  |  |  |  | 2710672 | KIAA2013 | -2.3 |
| 2680064 | SYVN1 | 2.4 |  |  |  |  |  |  | 2070349 | SNRPB | -2.3 |
| 4780615 | ANXA2 | 2.4 |  |  |  |  |  |  | 6250280 | PRDX1 | -2.2 |
| 1500600 | RAB37 | 2.4 |  |  |  |  |  |  | 7160504 | GPC1 | -2.2 |
| 6940242 | BRPF1 | 2.4 |  |  |  |  |  |  | 6380370 | CCND3 | -2.2 |
| 5570114 | GADD45G | 2.4 |  |  |  |  |  |  | 610440 | CD81 | -2.2 |
| 70634 | RABAC1 | 2.4 |  |  |  |  |  |  | 6620437 | HOXA5 | -2.2 |
| 1010487 | BTG2 | 2.4 |  |  |  |  |  |  | 1300369 | LOC255783 | -2.2 |
| 1230192 | SPNS1 | 2.4 |  |  |  |  |  |  | 2070376 | RFNG | -2.2 |
| 1010735 | DDX24 | 2.4 |  |  |  |  |  |  | 3140193 | NELF | -2.2 |
| 3310564 | PRDX5 | 2.4 |  |  |  |  |  |  | 6550593 | RPL38 | -2.2 |
| 1580309 | SERF2 | 2.4 |  |  |  |  |  |  | 4860315 | TESK1 | -2.2 |
| 6520128 | GPX4 | 2.4 |  |  |  |  |  |  | 4490671 | MIF | -2.2 |
| 6770309 | MYL6 | 2.4 |  |  |  |  |  |  | 2480709 | ADCK5 | -2.2 |
| 60138 | CTH | 2.4 |  |  |  |  |  |  | 5310634 | FASN | -2.2 |
| 1010195 | DBI | 2.3 |  |  |  |  |  |  | 4670343 | JAG2 | -2.2 |
| 2760735 | SYNM | 2.3 |  |  |  |  |  |  | 3400487 | LOC388474 | -2.2 |
| 4220632 | ATP1B3 | 2.3 |  |  |  |  |  |  | 1230754 | POR | -2.2 |
| 2970563 | RPRC1 | 2.3 |  |  |  |  |  |  | 1580309 | SERF2 | -2.2 |
| 430242 | DGUOK | 2.3 |  |  |  |  |  |  | 6330474 | CD151 | -2.2 |
| 7400050 | CCM2 | 2.3 |  |  |  |  |  |  | 670528 | GYG1 | -2.2 |
| 5050681 | TESC | 2.3 |  |  |  |  |  |  | 4210041 | UQCRQ | -2.2 |
| 1770753 | HRAS | 2.3 |  |  |  |  |  |  | 990747 | PSCD2 | -2.2 |
| 1230754 | POR | 2.3 |  |  |  |  |  |  | 5820360 | IMAA | -2.2 |
| 990747 | PSCD2 | 2.3 |  |  |  |  |  |  | 620433 | C1ORF122 | -2.2 |
| 5090176 | C9ORF89 | 2.3 |  |  |  |  |  |  | 6860661 | MGC16703 | -2.2 |
| 7510356 | DGKQ | 2.3 |  |  |  |  |  |  | 4730086 | SHCBP1 | -2.2 |
| 5820634 | LOC441150 | 2.3 |  |  |  |  |  |  | 1450273 | CLUAP1 | -2.2 |
| 580494 | PSMB4 | 2.3 |  |  |  |  |  |  | 7510356 | DGKQ | -2.2 |
| 650369 | PSMD8 | 2.3 |  |  |  |  |  |  | 1780661 | MAN1B1 | -2.2 |
| 5090068 | STX5 | 2.3 |  |  |  |  |  |  | 7200608 | HSPB1 | -2.2 |
| 5290500 | CAPNS1 | 2.3 |  |  |  |  |  |  | 650519 | TMBIM6 | -2.2 |
| 10487 | SLC25A25 | 2.3 |  |  |  |  |  |  | 5860608 | LOC400948 | -2.2 |
| 610440 | CD81 | 2.3 |  |  |  |  |  |  | 4780612 | UNC93B1 | -2.2 |
| 2750309 | MRPL53 | 2.3 |  |  |  |  |  |  | 4200692 | NEU1 | -2.2 |
| 3180541 | LOC440093 | 2.3 |  |  |  |  |  |  | 2600382 | GPX4 | -2.2 |
| 5670465 | ADM | 2.3 |  |  |  |  |  |  | 3460181 | MTCH1 | -2.2 |
| 4830682 | CCDC92 | 2.3 |  |  |  |  |  |  | 5310170 | CORO1B | -2.2 |
| 4670575 | HTATIP2 | 2.3 |  |  |  |  |  |  | 2000445 | PSMB10 | -2.2 |
| 430411 | TOR3A | 2.3 |  |  |  |  |  |  | 2230187 | MAPBPIP | -2.2 |
| 4880685 | FBXO22 | 2.3 |  |  |  |  |  |  | 3940364 | LOC347376 | -2.2 |
| 4760112 | ATP5J2 | 2.3 |  |  |  |  |  |  | 2680064 | SYVN1 | -2.2 |
| 4850754 | GHITM | 2.3 |  |  |  |  |  |  | 7210192 | ADA | -2.2 |
| 6280504 | LOC100008589 | 2.3 |  |  |  |  |  |  | 5560102 | AGPAT2 | -2.2 |
| 60653 | PRR7 | 2.3 |  |  |  |  |  |  | 610112 | FAM177A1 | -2.2 |
| 3890326 | SOD2 | 2.3 |  |  |  |  |  |  | 4900431 | STUB1 | -2.2 |
| 1820504 | NME1 | 2.3 |  |  |  |  |  |  | 3180470 | SDHA | -2.2 |
| 4920110 | GADD45B | 2.3 |  |  |  |  |  |  | 5960224 | PTTG3 | -2.2 |
| 5900575 | CD276 | 2.3 |  |  |  |  |  |  | 4280471 | GUK1 | -2.1 |
| 1660541 | ESRRA | 2.3 |  |  |  |  |  |  | 160170 | PKM2 | -2.1 |
| 20491 | UBE2F | 2.2 |  |  |  |  |  |  | 4760112 | ATP5J2 | -2.1 |
| 3930326 | LOC728014 | 2.2 |  |  |  |  |  |  | 7650017 | SERPINH1 | -2.1 |
| 5900438 | HOXC13 | 2.2 |  |  |  |  |  |  | 6510608 | BTBD11 | -2.1 |
| 4220180 | WDR68 | 2.2 |  |  |  |  |  |  | 770554 | LOC400963 | -2.1 |
| 5310634 | FASN | 2.2 |  |  |  |  |  |  | 2230296 | ORC6L | -2.1 |
| 5860215 | ABHD5 | 2.2 |  |  |  |  |  |  | 5910154 | NOL5A | -2.1 |
| 940300 | C17ORF90 | 2.2 |  |  |  |  |  |  | 2970521 | PGRMC1 | -2.1 |
| 4780612 | UNC93B1 | 2.2 |  |  |  |  |  |  | 4900333 | HAGH | -2.1 |
| 2450639 | TSC22D1 | 2.2 |  |  |  |  |  |  | 3120114 | HIST1H2AM | -2.1 |
| 5490608 | SELS | 2.2 |  |  |  |  |  |  | 1170609 | ITIH5 | -2.1 |
| 4730343 | ATP6V0E1 | 2.2 |  |  |  |  |  |  | 1940709 | LOC645895 | -2.1 |
| 7160504 | GPC1 | 2.2 |  |  |  |  |  |  | 1300671 | NCOA4 | -2.1 |
| 5890605 | FAM109A | 2.2 |  |  |  |  |  |  | 4880673 | GADD45A | -2.1 |
| 3610646 | LOC401019 | 2.2 |  |  |  |  |  |  | 60653 | PRR7 | -2.1 |
| 2650524 | RPL34 | 2.2 |  |  |  |  |  |  | 1170736 | MMP15 | -2.1 |
| 5870521 | HLA-H | 2.2 |  |  |  |  |  |  | 1510609 | NME1-NME2 | -2.1 |
| 3840689 | PIGT | 2.2 |  |  |  |  |  |  | 3130072 | RHOT2 | -2.1 |
| 6280168 | SERPINA3 | 2.2 |  |  |  |  |  |  | 2190537 | WDR74 | -2.1 |
| 2190341 | AP1S1 | 2.2 |  |  |  |  |  |  | 2810082 | C20ORF111 | -2.1 |
| 6370369 | CD14 | 2.2 |  |  |  |  |  |  | 4010433 | LOC650832 | -2.1 |
| 4560064 | GLB1 | 2.2 |  |  |  |  |  |  | 3440630 | ESPN | -2.1 |
| 1110095 | CLTB | 2.2 |  |  |  |  |  |  | 1170164 | RPL35 | -2.1 |
| 5810088 | AURKAIP1 | 2.2 |  |  |  |  |  |  | 3460278 | C22ORF13 | -2.1 |
| 4120609 | DGUOK | 2.2 |  |  |  |  |  |  | 3520092 | BAX | -2.1 |
| 6590201 | ATP6AP1 | 2.2 |  |  |  |  |  |  | 2710292 | H2AFZ | -2.1 |
| 2600382 | GPX4 | 2.2 |  |  |  |  |  |  | 3170093 | KIAA0247 | -2.1 |
| 4880433 | IDH2 | 2.2 |  |  |  |  |  |  | 6220180 | CABC1 | -2.1 |
| 1230220 | LOC729466 | 2.2 |  |  |  |  |  |  | 1500600 | RAB37 | -2.1 |
| 1690059 | CALML5 | 2.2 |  |  |  |  |  |  | 7160743 | F2R | -2.1 |
| 5570152 | ATP1A1 | 2.2 |  |  |  |  |  |  | 6330377 | ATP6V0D1 | -2.1 |
| 4610431 | ACTG2 | 2.2 |  |  |  |  |  |  | 5310707 | EI24 | -2.1 |
| 7650017 | SERPINH1 | 2.2 |  |  |  |  |  |  | 2650524 | RPL34 | -2.1 |
| 6510608 | BTBD11 | 2.2 |  |  |  |  |  |  | 5670093 | ECHS1 | -2.1 |
| 1690360 | KREMEN2 | 2.2 |  |  |  |  |  |  | 160242 | C13ORF15 | -2.1 |
| 160242 | C13ORF15 | 2.2 |  |  |  |  |  |  | 4220180 | WDR68 | -2.1 |
| 1940228 | FAM58A | 2.2 |  |  |  |  |  |  | 6620403 | AGBL5 | -2.1 |
| 1170736 | MMP15 | 2.2 |  |  |  |  |  |  | 7040139 | ZNF324 | -2.1 |
| 3800050 | ADCY3 | 2.2 |  |  |  |  |  |  | 4860367 | ATRIP | -2.1 |
| 2350066 | HLA-A | 2.2 |  |  |  |  |  |  | 6620392 | LFNG | -2.1 |
| 5700370 | POLDIP3 | 2.2 |  |  |  |  |  |  | 2630022 | RNPS1 | -2.1 |
| 5570324 | BMP1 | 2.2 |  |  |  |  |  |  | 5090671 | GDF15 | -2.1 |
| 6380128 | CLTA | 2.2 |  |  |  |  |  |  | 4230360 | AP3B2 | -2.1 |
| 4880551 | RBM42 | 2.2 |  |  |  |  |  |  | 6370762 | POLR2F | -2.1 |
| 840296 | LOC440926 | 2.2 |  |  |  |  |  |  | 5900445 | ARFGAP1 | -2.1 |
| 7100136 | HES2 | 2.2 |  |  |  |  |  |  | 5820634 | LOC441150 | -2.1 |
| 3440452 | ADAM15 | 2.2 |  |  |  |  |  |  | 4900170 | GPS2 | -2.1 |
| 1710189 | RHBDF1 | 2.2 |  |  |  |  |  |  | 520184 | HPCAL1 | -2.1 |
| 7210017 | NOMO2 | 2.2 |  |  |  |  |  |  | 3940692 | SBF1 | -2.1 |
| 3940692 | SBF1 | 2.2 |  |  |  |  |  |  | 3830477 | LOC441246 | -2.1 |
| 1740326 | CSGLCA-T | 2.2 |  |  |  |  |  |  | 6450056 | MCOLN1 | -2.1 |
| 1740576 | LMF2 | 2.2 |  |  |  |  |  |  | 5810201 | ZNF593 | -2.1 |
| 3930189 | EIF4G1 | 2.2 |  |  |  |  |  |  | 5860148 | S100A13 | -2.1 |
| 4250095 | COX6B1 | 2.2 |  |  |  |  |  |  | 2340241 | IMPA2 | -2.1 |
| 5360553 | ECH1 | 2.2 |  |  |  |  |  |  | 6400437 | RPL32 | -2.1 |
| 5080692 | HLA-A29.1 | 2.2 |  |  |  |  |  |  | 7400747 | FAM89A | -2.1 |
| 3890017 | CTNNA1 | 2.2 |  |  |  |  |  |  | 5810746 | MATN2 | -2.1 |
| 4810129 | BRMS1 | 2.2 |  |  |  |  |  |  | 830066 | RPS24 | -2.1 |
| 5560102 | AGPAT2 | 2.2 |  |  |  |  |  |  | 3520753 | ACAA1 | -2.1 |
| 3060128 | CHSY1 | 2.2 |  |  |  |  |  |  | 6590463 | UBB | -2.1 |
| 2940301 | HPS6 | 2.1 |  |  |  |  |  |  | 3060128 | CHSY1 | -2.1 |
| 1170440 | AHCYL1 | 2.1 |  |  |  |  |  |  | 7100136 | HES2 | -2.1 |
| 4480220 | FGFRL1 | 2.1 |  |  |  |  |  |  | 2570156 | GGA1 | -2.1 |
| 7400747 | FAM89A | 2.1 |  |  |  |  |  |  | 5130253 | HGS | -2.1 |
| 6900195 | CNO | 2.1 |  |  |  |  |  |  | 5420367 | SOD1 | -2.1 |
| 240195 | B2M | 2.1 |  |  |  |  |  |  | 70661 | CCDC72 | -2.1 |
| 1190220 | COX5B | 2.1 |  |  |  |  |  |  | 3460441 | EDF1 | -2.1 |
| 4880168 | PTTG1IP | 2.1 |  |  |  |  |  |  | 2750309 | MRPL53 | -2.1 |
| 3190112 | SERPINB1 | 2.1 |  |  |  |  |  |  | 3990368 | PPP1CA | -2.1 |
| 1340689 | RPN1 | 2.1 |  |  |  |  |  |  | 770168 | CLPTM1 | -2.1 |
| 7210192 | ADA | 2.1 |  |  |  |  |  |  | 1300678 | GRIN2C | -2.1 |
| 4590154 | ZDHHC8 | 2.1 |  |  |  |  |  |  | 630327 | RET | -2.1 |
| 650519 | TMBIM6 | 2.1 |  |  |  |  |  |  | 1990717 | C1ORF63 | -2.1 |
| 2850100 | RNASET2 | 2.1 |  |  |  |  |  |  | 1710189 | RHBDF1 | -2.1 |
| 6040156 | C6ORF52 | 2.1 |  |  |  |  |  |  | 6130725 | ARHGEF18 | -2.1 |
| 2360491 | NARF | 2.1 |  |  |  |  |  |  | 6980224 | HS.128463 | -2.1 |
| 6510487 | FAM96B | 2.1 |  |  |  |  |  |  | 2470619 | AKT1 | -2.1 |
| 20673 | BSG | 2.1 |  |  |  |  |  |  | 1510088 | ATP1B3 | -2.1 |
| 1510609 | NME1-NME2 | 2.1 |  |  |  |  |  |  | 4490161 | GAPDH | -2.1 |
| 4560110 | ARMET | 2.1 |  |  |  |  |  |  | 4810129 | BRMS1 | -2.0 |
| 4120086 | LAMC1 | 2.1 |  |  |  |  |  |  | 3420372 | RBM38 | -2.0 |
| 2600279 | GPS1 | 2.1 |  |  |  |  |  |  | 3890326 | SOD2 | -2.0 |
| 3310301 | LOC646531 | 2.1 |  |  |  |  |  |  | 240309 | CTSB | -2.0 |
| 5820672 | HIC2 | 2.1 |  |  |  |  |  |  | 4040008 | LOC646195 | -2.0 |
| 5570678 | RPS27L | 2.1 |  |  |  |  |  |  | 5820672 | HIC2 | -2.0 |
| 1940021 | GRN | 2.1 |  |  |  |  |  |  | 5820528 | TSEN34 | -2.0 |
| 7040139 | ZNF324 | 2.1 |  |  |  |  |  |  | 1190220 | COX5B | -2.0 |
| 5390347 | ZNF79 | 2.1 |  |  |  |  |  |  | 1090026 | PMPCA | -2.0 |
| 3120139 | NENF | 2.1 |  |  |  |  |  |  | 6200431 | AKT1 | -2.0 |
| 270133 | C1ORF128 | 2.1 |  |  |  |  |  |  | 5860215 | ABHD5 | -2.0 |
| 6110392 | GNS | 2.1 |  |  |  |  |  |  | 5860242 | LOC642755 | -2.0 |
| 5310494 | LOC440733 | 2.1 |  |  |  |  |  |  | 6650564 | RPS27 | -2.0 |
| 6100768 | CYFIP2 | 2.1 |  |  |  |  |  |  | 7040670 | DHRS3 | -2.0 |
| 6290168 | C10ORF116 | 2.1 |  |  |  |  |  |  | 290132 | C20ORF117 | -2.0 |
| 240309 | CTSB | 2.1 |  |  |  |  |  |  | 3830605 | DNLZ | -2.0 |
| 6980685 | LOC730740 | 2.1 |  |  |  |  |  |  | 3390477 | C17ORF79 | -2.0 |
| 2070376 | RFNG | 2.1 |  |  |  |  |  |  | 6040634 | IRX2 | -2.0 |
| 3120520 | GABARAPL2 | 2.1 |  |  |  |  |  |  | 7330026 | FRAT2 | -2.0 |
| 2030678 | HIST2H2AB | 2.1 |  |  |  |  |  |  | 4250095 | COX6B1 | -2.0 |
| 6860593 | HS.568928 | 2.1 |  |  |  |  |  |  | 3890017 | CTNNA1 | -2.0 |
| 1260440 | PRKAB1 | 2.1 |  |  |  |  |  |  | 630474 | ZP3 | -2.0 |
| 4890487 | B2M | 2.1 |  |  |  |  |  |  | 6770286 | MCART1 | -2.0 |
| 7040315 | STX16 | 2.1 |  |  |  |  |  |  | 7610131 | EPAS1 | -2.0 |
| 2970026 | ZNF622 | 2.1 |  |  |  |  |  |  | 4480288 | ISG20L1 | -2.0 |
| 1300678 | GRIN2C | 2.1 |  |  |  |  |  |  | 1770753 | HRAS | -2.0 |
| 6280167 | LOC401115 | 2.1 |  |  |  |  |  |  | 4540600 | FAM115A | -2.0 |
| 3370112 | RPN2 | 2.1 |  |  |  |  |  |  | 2510411 | SLC44A4 | -2.0 |
| 2030093 | PKM2 | 2.1 |  |  |  |  |  |  | 540491 | COX7A2 | -2.0 |
| 4230360 | AP3B2 | 2.1 |  |  |  |  |  |  | 940435 | TRIM8 | -2.0 |
| 6860753 | TSPO | 2.1 |  |  |  |  |  |  | 430242 | DGUOK | -2.0 |
| 6620403 | AGBL5 | 2.1 |  |  |  |  |  |  | 1230220 | LOC729466 | -2.0 |
| 2480709 | ADCK5 | 2.1 |  |  |  |  |  |  | 4120609 | DGUOK | -2.0 |
| 7570671 | UNKL | 2.1 |  |  |  |  |  |  | 2600279 | GPS1 | -2.0 |
| 7550041 | CAPNS1 | 2.1 |  |  |  |  |  |  | 5820255 | HSPC171 | -2.0 |
| 1170647 | ACADVL | 2.1 |  |  |  |  |  |  | 7400025 | MTP18 | -2.0 |
| 6980224 | HS.128463 | 2.1 |  |  |  |  |  |  | 6380445 | SFRS5 | -2.0 |
| 1230025 | CYB5R1 | 2.1 |  |  |  |  |  |  |  |  |  |
| 4760349 | LRP10 | 2.1 |  |  |  |  |  |  |  |  |  |
| 1470397 | C8ORF33 | 2.1 |  |  |  |  |  |  |  |  |  |
| 2710068 | EIF3I | 2.0 |  |  |  |  |  |  |  |  |  |
| 4760474 | TUBA4A | 2.0 |  |  |  |  |  |  |  |  |  |
| 1010068 | FKBP2 | 2.0 |  |  |  |  |  |  |  |  |  |
| 1230600 | LOC651064 | 2.0 |  |  |  |  |  |  |  |  |  |
| 3440630 | ESPN | 2.0 |  |  |  |  |  |  |  |  |  |
| 6040376 | FAM127A | 2.0 |  |  |  |  |  |  |  |  |  |
| 6350632 | TSC22D3 | 2.0 |  |  |  |  |  |  |  |  |  |
| 5390202 | TMED9 | 2.0 |  |  |  |  |  |  |  |  |  |
| 7320435 | DPM3 | 2.0 |  |  |  |  |  |  |  |  |  |
| 3370202 | ANXA2P1 | 2.0 |  |  |  |  |  |  |  |  |  |
| 6580608 | PEX16 | 2.0 |  |  |  |  |  |  |  |  |  |
| 6200017 | POLR2L | 2.0 |  |  |  |  |  |  |  |  |  |
| 2320110 | ATP6V0E1 | 2.0 |  |  |  |  |  |  |  |  |  |
| 5310379 | TMEM115 | 2.0 |  |  |  |  |  |  |  |  |  |
| 2490259 | PINK1 | 2.0 |  |  |  |  |  |  |  |  |  |
| 5310465 | MRPS12 | 2.0 |  |  |  |  |  |  |  |  |  |
| 1450082 | TRAPPC2L | 2.0 |  |  |  |  |  |  |  |  |  |
| 3450427 | MED19 | 2.0 |  |  |  |  |  |  |  |  |  |
| 5860148 | S100A13 | 2.0 |  |  |  |  |  |  |  |  |  |
| 5090561 | ATP5EP2 | 2.0 |  |  |  |  |  |  |  |  |  |
| 3890681 | SLC41A3 | 2.0 |  |  |  |  |  |  |  |  |  |
| 4040564 | IRAK1 | 2.0 |  |  |  |  |  |  |  |  |  |
| 20022 | SRF | 2.0 |  |  |  |  |  |  |  |  |  |
| 1300369 | LOC255783 | 2.0 |  |  |  |  |  |  |  |  |  |
| 4900170 | GPS2 | 2.0 |  |  |  |  |  |  |  |  |  |
| 4570468 | ZNF263 | 2.0 |  |  |  |  |  |  |  |  |  |
| 3940364 | LOC347376 | 2.0 |  |  |  |  |  |  |  |  |  |
| 3420372 | RBM38 | 2.0 |  |  |  |  |  |  |  |  |  |
| 2850026 | H3F3A | 2.0 |  |  |  |  |  |  |  |  |  |
| 5050437 | SCAND1 | 2.0 |  |  |  |  |  |  |  |  |  |
| 2360682 | PSMB6 | 2.0 |  |  |  |  |  |  |  |  |  |
| 3400709 | HS.531457 | 2.0 |  |  |  |  |  |  |  |  |  |
| 2650193 | SDC4 | 2.0 |  |  |  |  |  |  |  |  |  |
| 6450162 | C9ORF169 | 2.0 |  |  |  |  |  |  |  |  |  |
| 70661 | CCDC72 | 2.0 |  |  |  |  |  |  |  |  |  |
| 5080167 | NOTCH1 | 2.0 |  |  |  |  |  |  |  |  |  |
| 2120544 | IMP3 | 2.0 |  |  |  |  |  |  |  |  |  |
| 6040634 | IRX2 | 2.0 |  |  |  |  |  |  |  |  |  |
| 6370661 | AP2S1 | 2.0 |  |  |  |  |  |  |  |  |  |
| 3520671 | AGPAT2 | 2.0 |  |  |  |  |  |  |  |  |  |
| 70592 | VCL | 2.0 |  |  |  |  |  |  |  |  |  |
| 6590253 | ALDOA | 2.0 |  |  |  |  |  |  |  |  |  |
| 6270100 | LAMP1 | 2.0 |  |  |  |  |  |  |  |  |  |
| 4060041 | MYST1 | 2.0 |  |  |  |  |  |  |  |  |  |
