# Supplementary material for: TFPI1 Mediates Resistance to Doxorubicin in Breast Cancer Cells by Inducing a Hypoxic-Like Response
Source: PLoS One. 2014 Jan 28;9(1):e84611. doi: 10.1371/journal.pone.0084611 (PMC3904823; doi:10.1371/journal.pone.0084611)
Supplement: Table S3 — Reversion of gene expression changes following the 2-week chronic exposure to 1 nM DOX. Edges 3–7 and 4–8 refer to the numbering system described in Fig. S1. (DOCX) [file pone.0084611.s010.docx]

**Supplementary Table 3 Reversion of gene expression changes following the 2-week chronic exposure to 1 nM DOX.** Edges 3-7 and 4-8 refer to the numbering system described in Suppl. Fig. 1.

| **Edge 3-7 (396) - UP, DOWN** | |  |  | **Edge 4-8 (68) - DOWN, UP** | |  |
| --- | --- | --- | --- | --- | --- | --- |
|  |  |  |  |  |  |  |
| **Probe.ID** | **TargetID** | **FC** |  | **Probe.ID** | **TargetID** | **FC** |
| 50309 | DPYSL4 | -23.5 |  | 3170286 | LOC647000 | -6.0 |
| 3130220 | TMEM158 | -22.6 |  | 6510176 | TUBA1B | -5.4 |
| 4560328 | FSCN1 | -19.2 |  | 2510019 | EIF3E | -5.0 |
| 1260020 | TP53I3 | -17.1 |  | 4230091 | HNRPA2B1 | -5.0 |
| 6520139 | FGFR3 | -14.8 |  | 450424 | CXCR7 | -4.9 |
| 2900274 | VASN | -12.7 |  | 6650079 | C3ORF14 | -4.6 |
| 1010333 | MSX1 | -12.3 |  | 6860047 | HS.579631 | -4.3 |
| 7330392 | TAP1 | -11.0 |  | 6580577 | HNRNPD | -4.0 |
| 2070360 | C17ORF82 | -9.9 |  | 270593 | GPER | -3.9 |
| 6480059 | ACTA2 | -9.7 |  | 3190092 | LDHA | -3.9 |
| 3710040 | SFN | -9.6 |  | 520463 | GPER | -3.2 |
| 610451 | HIST2H2AA3 | -9.0 |  | 4760243 | LOC648210 | -3.2 |
| 3870678 | HIST1H2AE | -8.8 |  | 5870474 | RHOBTB3 | -3.2 |
| 4290050 | ACOT7 | -8.7 |  | 1260162 | DNMT1 | -3.1 |
| 1510008 | GALR2 | -8.7 |  | 520209 | SRP9 | -3.1 |
| 6100022 | HIST2H2AC | -8.3 |  | 610437 | CD24 | -3.0 |
| 5090750 | FOXC1 | -7.7 |  | 4640689 | EIF4A2 | -3.0 |
| 7610615 | SLC6A10P | -7.1 |  | 1940593 | LOC653226 | -3.0 |
| 7570484 | TFF3 | -7.0 |  | 6960025 | DPY30 | -2.9 |
| 780528 | CKS2 | -6.8 |  | 4540349 | LSM2 | -2.9 |
| 7550064 | KCTD5 | -6.8 |  | 5820601 | CCND1 | -2.8 |
| 5310411 | H2AFJ | -6.4 |  | 2710192 | MRPL3 | -2.8 |
| 3170100 | BMP7 | -6.1 |  | 6760037 | SYTL2 | -2.8 |
| 830278 | GLIPR2 | -6.0 |  | 630243 | ATAD4 | -2.7 |
| 610201 | HES6 | -6.0 |  | 7330612 | ATP5G2 | -2.7 |
| 4490475 | SLC30A3 | -5.4 |  | 7320382 | SLC38A1 | -2.7 |
| 6840075 | NP | -5.3 |  | 1740136 | SLC38A2 | -2.7 |
| 5080192 | SERPINE2 | -5.3 |  | 2680128 | TMBIM4 | -2.7 |
| 3990598 | DRAP1 | -5.1 |  | 5690066 | XBP1 | -2.6 |
| 6650746 | RHBDD2 | -5.0 |  | 650168 | C17ORF58 | -2.5 |
| 270152 | SLC7A5 | -5.0 |  | 6840577 | KPNB1 | -2.5 |
| 4780128 | ATF3 | -4.9 |  | 4010347 | COPS5 | -2.4 |
| 6020424 | LMNA | -4.7 |  | 5960021 | HS.57079 | -2.4 |
| 4560717 | MDK | -4.7 |  | 7150433 | TCTEX1D2 | -2.4 |
| 510373 | RHBDD2 | -4.7 |  | 4120553 | WISP2 | -2.4 |
| 990500 | AVPI1 | -4.6 |  | 1850468 | CCDC56 | -2.3 |
| 3290338 | POLR2A | -4.6 |  | 1010048 | CXXC5 | -2.3 |
| 6590592 | SDC1 | -4.6 |  | 2940079 | ENY2 | -2.3 |
| 2600463 | TNFRSF10B | -4.6 |  | 5900746 | GNL3L | -2.3 |
| 6200468 | CKS2 | -4.5 |  | 4900070 | GSTO1 | -2.3 |
| 2650521 | LRPAP1 | -4.5 |  | 2000220 | HEATR6 | -2.3 |
| 6660132 | DCXR | -4.4 |  | 6580131 | MRPL22 | -2.3 |
| 4570102 | BRMS1 | -4.3 |  | 7150152 | PTPLAD1 | -2.3 |
| 3390730 | DOLK | -4.3 |  | 1510202 | RWDD1 | -2.3 |
| 6650035 | LOC338758 | -4.3 |  | 3710154 | SLC7A2 | -2.3 |
| 4560164 | PDLIM7 | -4.3 |  | 2940044 | TMEM14B | -2.3 |
| 5490431 | SAT1 | -4.3 |  | 290603 | AARS | -2.2 |
| 5490673 | AP1S1 | -4.2 |  | 1470706 | C8ORF55 | -2.2 |
| 6380717 | HSPA1A | -4.2 |  | 5090088 | EPRS | -2.2 |
| 6400195 | MRPS6 | -4.2 |  | 650605 | LOC388789 | -2.2 |
| 5860187 | PHYH | -4.2 |  | 60670 | LXN | -2.2 |
| 7570673 | UPP1 | -4.2 |  | 2120452 | MLPH | -2.2 |
| 6220086 | DYNLL1 | -4.1 |  | 2340372 | MRFAP1 | -2.2 |
| 2140735 | RALGDS | -4.1 |  | 3420543 | PSMA4 | -2.2 |
| 4250458 | CFL1 | -4.0 |  | 6860300 | SMARCA4 | -2.2 |
| 5900592 | MGAT1 | -4.0 |  | 6270138 | TACSTD2 | -2.2 |
| 3170162 | PRNP | -4.0 |  | 4850731 | TFPI | -2.2 |
| 3140750 | RBM38 | -4.0 |  | 6110754 | ATP5O | -2.1 |
| 2360753 | TBX2 | -4.0 |  | 4560022 | C2ORF25 | -2.1 |
| 2810367 | ABCB6 | -3.9 |  | 7380110 | CDK4 | -2.1 |
| 1850075 | AGBL5 | -3.9 |  | 4860114 | CXCR7 | -2.1 |
| 5130162 | AK1 | -3.9 |  | 610324 | DDX1 | -2.1 |
| 5490356 | NXF1 | -3.9 |  | 3390544 | HSP90AA1 | -2.1 |
| 5870326 | SLC2A8 | -3.9 |  | 3060110 | SFRS6 | -2.1 |
| 1780538 | SLC6A8 | -3.9 |  | 1400070 | TBC1D9 | -2.1 |
| 2470689 | SPHK1 | -3.9 |  | 3450072 | CLIC1 | -2.0 |
| 6250131 | CABYR | -3.8 |  | 7610747 | MRPL24 | -2.0 |
| 2900543 | ENDOG | -3.8 |  | 4290605 | SLC44A1 | -2.0 |
| 1510424 | S100P | -3.8 |  |  |  |  |
| 7650047 | SLC29A4 | -3.8 |  |  |  |  |
| 1430681 | TLCD1 | -3.8 |  |  |  |  |
| 6560441 | XPC | -3.8 |  |  |  |  |
| 4390121 | ADRM1 | -3.7 |  |  |  |  |
| 20446 | CEBPB | -3.7 |  |  |  |  |
| 6200132 | FZD9 | -3.7 |  |  |  |  |
| 1500192 | HIST3H2A | -3.7 |  |  |  |  |
| 1190739 | ITPRIPL2 | -3.7 |  |  |  |  |
| 1990546 | KIAA0152 | -3.7 |  |  |  |  |
| 7550070 | LOC730316 | -3.7 |  |  |  |  |
| 4850497 | NXF1 | -3.7 |  |  |  |  |
| 1240221 | POLR2H | -3.7 |  |  |  |  |
| 4210692 | SAC3D1 | -3.7 |  |  |  |  |
| 7200242 | SULF2 | -3.7 |  |  |  |  |
| 5910364 | TYMS | -3.7 |  |  |  |  |
| 4230520 | DNCL1 | -3.6 |  |  |  |  |
| 940750 | HS.553217 | -3.6 |  |  |  |  |
| 6660315 | INPP1 | -3.6 |  |  |  |  |
| 6420369 | MDH2 | -3.6 |  |  |  |  |
| 5690711 | TMEM134 | -3.6 |  |  |  |  |
| 3310477 | TPI1 | -3.6 |  |  |  |  |
| 4210095 | ATP1A1 | -3.5 |  |  |  |  |
| 4490259 | COX8A | -3.5 |  |  |  |  |
| 6280504 | LOC100008589 | -3.5 |  |  |  |  |
| 3710647 | MXD4 | -3.5 |  |  |  |  |
| 670671 | TMEM134 | -3.5 |  |  |  |  |
| 610356 | ZDHHC9 | -3.5 |  |  |  |  |
| 6560390 | CRELD2 | -3.4 |  |  |  |  |
| 4880433 | IDH2 | -3.4 |  |  |  |  |
| 2630768 | LMNA | -3.4 |  |  |  |  |
| 5560075 | MFGE8 | -3.4 |  |  |  |  |
| 4810128 | PHLDA2 | -3.4 |  |  |  |  |
| 990176 | RN7SK | -3.4 |  |  |  |  |
| 10133 | SLC9A1 | -3.4 |  |  |  |  |
| 1780619 | EHD1 | -3.3 |  |  |  |  |
| 1820594 | HBEGF | -3.3 |  |  |  |  |
| 3610646 | LOC401019 | -3.3 |  |  |  |  |
| 2260619 | MIB2 | -3.3 |  |  |  |  |
| 4670021 | NPEPL1 | -3.3 |  |  |  |  |
| 110661 | TSPAN4 | -3.3 |  |  |  |  |
| 60121 | CTSB | -3.2 |  |  |  |  |
| 6450605 | DDOST | -3.2 |  |  |  |  |
| 3890521 | DEDD2 | -3.2 |  |  |  |  |
| 2470386 | DEXI | -3.2 |  |  |  |  |
| 4280273 | GM2A | -3.2 |  |  |  |  |
| 1990520 | GPS1 | -3.2 |  |  |  |  |
| 7380706 | NINJ1 | -3.2 |  |  |  |  |
| 7550343 | PRDX6 | -3.2 |  |  |  |  |
| 2710735 | RASD1 | -3.2 |  |  |  |  |
| 650634 | SLC25A39 | -3.2 |  |  |  |  |
| 3800050 | ADCY3 | -3.1 |  |  |  |  |
| 3370164 | ATP1A1 | -3.1 |  |  |  |  |
| 6450138 | BCAP31 | -3.1 |  |  |  |  |
| 780341 | CCDC3 | -3.1 |  |  |  |  |
| 3130079 | EIF2B2 | -3.1 |  |  |  |  |
| 870537 | LOC134997 | -3.1 |  |  |  |  |
| 1500619 | LOC642755 | -3.1 |  |  |  |  |
| 1110338 | MYL6 | -3.1 |  |  |  |  |
| 5960682 | RBPMS2 | -3.1 |  |  |  |  |
| 3420523 | RHBDF2 | -3.1 |  |  |  |  |
| 6020564 | RRAGA | -3.1 |  |  |  |  |
| 1940129 | SIRPA | -3.1 |  |  |  |  |
| 1940360 | TPI1 | -3.1 |  |  |  |  |
| 630706 | BTBD14A | -3.0 |  |  |  |  |
| 2600286 | CCS | -3.0 |  |  |  |  |
| 3940482 | CDR2L | -3.0 |  |  |  |  |
| 7570494 | CNFN | -3.0 |  |  |  |  |
| 6450543 | CST3 | -3.0 |  |  |  |  |
| 5090021 | HS.535392 | -3.0 |  |  |  |  |
| 1260086 | ID2 | -3.0 |  |  |  |  |
| 840296 | LOC440926 | -3.0 |  |  |  |  |
| 6770309 | MYL6 | -3.0 |  |  |  |  |
| 2850180 | NUDT16L1 | -3.0 |  |  |  |  |
| 4070017 | ODC1 | -3.0 |  |  |  |  |
| 3390551 | C14ORF78 | -2.9 |  |  |  |  |
| 990161 | CDIPT | -2.9 |  |  |  |  |
| 1430280 | CEBPA | -2.9 |  |  |  |  |
| 4900440 | CIB1 | -2.9 |  |  |  |  |
| 4180079 | CRISPLD2 | -2.9 |  |  |  |  |
| 2850026 | H3F3A | -2.9 |  |  |  |  |
| 780168 | ISCU | -2.9 |  |  |  |  |
| 2100196 | ISG15 | -2.9 |  |  |  |  |
| 580132 | LAPTM4B | -2.9 |  |  |  |  |
| 4730025 | LOC341457 | -2.9 |  |  |  |  |
| 1470608 | ROM1 | -2.9 |  |  |  |  |
| 3610397 | ATP6V0C | -2.8 |  |  |  |  |
| 4760338 | CDC25B | -2.8 |  |  |  |  |
| 5390161 | DUSP5 | -2.8 |  |  |  |  |
| 2060121 | FUCA1 | -2.8 |  |  |  |  |
| 6110630 | HIST1H2BK | -2.8 |  |  |  |  |
| 5310494 | LOC440733 | -2.8 |  |  |  |  |
| 2060440 | MAFB | -2.8 |  |  |  |  |
| 1510703 | MFSD3 | -2.8 |  |  |  |  |
| 4150687 | NDUFB2 | -2.8 |  |  |  |  |
| 5670594 | NMB | -2.8 |  |  |  |  |
| 4730685 | RAB7A | -2.8 |  |  |  |  |
| 5420575 | SLC3A2 | -2.8 |  |  |  |  |
| 7570243 | SSR4 | -2.8 |  |  |  |  |
| 3710324 | SURF1 | -2.8 |  |  |  |  |
| 270129 | TMEM8 | -2.8 |  |  |  |  |
| 3710609 | TSPAN17 | -2.8 |  |  |  |  |
| 4220519 | UBA52 | -2.8 |  |  |  |  |
| 4860286 | UBB | -2.8 |  |  |  |  |
| 630259 | VPS37D | -2.8 |  |  |  |  |
| 6580639 | ACOT7 | -2.7 |  |  |  |  |
| 5560131 | ATOX1 | -2.7 |  |  |  |  |
| 70767 | BAIAP2 | -2.7 |  |  |  |  |
| 6940242 | BRPF1 | -2.7 |  |  |  |  |
| 7400050 | CCM2 | -2.7 |  |  |  |  |
| 3450156 | CSNK2B | -2.7 |  |  |  |  |
| 1990196 | DACT3 | -2.7 |  |  |  |  |
| 770541 | EI24 | -2.7 |  |  |  |  |
| 5270110 | EIF4A3 | -2.7 |  |  |  |  |
| 2360020 | FADS1 | -2.7 |  |  |  |  |
| 6510519 | IL17D | -2.7 |  |  |  |  |
| 1690360 | KREMEN2 | -2.7 |  |  |  |  |
| 6650348 | LAPTM4B | -2.7 |  |  |  |  |
| 7160246 | LOC339123 | -2.7 |  |  |  |  |
| 3180541 | LOC440093 | -2.7 |  |  |  |  |
| 3310301 | LOC646531 | -2.7 |  |  |  |  |
| 6980685 | LOC730740 | -2.7 |  |  |  |  |
| 6380220 | MAGMAS | -2.7 |  |  |  |  |
| 6180446 | PRDX1 | -2.7 |  |  |  |  |
| 6400138 | PRMT1 | -2.7 |  |  |  |  |
| 4390703 | PTPN1 | -2.7 |  |  |  |  |
| 380050 | RHBDF2 | -2.7 |  |  |  |  |
| 4290072 | SERTAD1 | -2.7 |  |  |  |  |
| 1430647 | TAX1BP3 | -2.7 |  |  |  |  |
| 1230672 | TFF1 | -2.7 |  |  |  |  |
| 60397 | TRK1 | -2.7 |  |  |  |  |
| 7150475 | WDR1 | -2.7 |  |  |  |  |
| 4280136 | ADRM1 | -2.6 |  |  |  |  |
| 4830056 | ARPC5L | -2.6 |  |  |  |  |
| 6860543 | ATP6V1E1 | -2.6 |  |  |  |  |
| 4260386 | CTSL1 | -2.6 |  |  |  |  |
| 4920110 | GADD45B | -2.6 |  |  |  |  |
| 7510634 | GPRC5C | -2.6 |  |  |  |  |
| 1030747 | HLA-E | -2.6 |  |  |  |  |
| 2070491 | HS.565887 | -2.6 |  |  |  |  |
| 2320689 | LOC653610 | -2.6 |  |  |  |  |
| 6580753 | MIB2 | -2.6 |  |  |  |  |
| 3370575 | NADSYN1 | -2.6 |  |  |  |  |
| 4780707 | OTUB1 | -2.6 |  |  |  |  |
| 5390411 | PHPT1 | -2.6 |  |  |  |  |
| 5690554 | PPM1D | -2.6 |  |  |  |  |
| 4890181 | RAP1GAP | -2.6 |  |  |  |  |
| 10487 | SLC25A25 | -2.6 |  |  |  |  |
| 20022 | SRF | -2.6 |  |  |  |  |
| 1010647 | TP53I13 | -2.6 |  |  |  |  |
| 4850487 | UFSP1 | -2.6 |  |  |  |  |
| 4200259 | ACLY | -2.5 |  |  |  |  |
| 6370661 | AP2S1 | -2.5 |  |  |  |  |
| 6590593 | ATP5J2 | -2.5 |  |  |  |  |
| 5810088 | AURKAIP1 | -2.5 |  |  |  |  |
| 3460386 | B4GALT1 | -2.5 |  |  |  |  |
| 7510608 | C12ORF45 | -2.5 |  |  |  |  |
| 4150670 | CDC37 | -2.5 |  |  |  |  |
| 4230201 | CDKN1A | -2.5 |  |  |  |  |
| 60138 | CTH | -2.5 |  |  |  |  |
| 1010195 | DBI | -2.5 |  |  |  |  |
| 6770630 | DHDH | -2.5 |  |  |  |  |
| 5090053 | FDX1L | -2.5 |  |  |  |  |
| 6100075 | GNAS | -2.5 |  |  |  |  |
| 2480288 | GPAA1 | -2.5 |  |  |  |  |
| 1440296 | H3F3B | -2.5 |  |  |  |  |
| 2030678 | HIST2H2AB | -2.5 |  |  |  |  |
| 4230678 | HIST2H2BE | -2.5 |  |  |  |  |
| 3400438 | HLA-A | -2.5 |  |  |  |  |
| 1660296 | ID2 | -2.5 |  |  |  |  |
| 1780678 | LOC374395 | -2.5 |  |  |  |  |
| 3120544 | METRN | -2.5 |  |  |  |  |
| 5570494 | MRPL33 | -2.5 |  |  |  |  |
| 620615 | NDUFA7 | -2.5 |  |  |  |  |
| 2900594 | PGD | -2.5 |  |  |  |  |
| 7650477 | STUB1 | -2.5 |  |  |  |  |
| 6860753 | TSPO | -2.5 |  |  |  |  |
| 6420541 | UBL5 | -2.5 |  |  |  |  |
| 3440452 | ADAM15 | -2.4 |  |  |  |  |
| 6590253 | ALDOA | -2.4 |  |  |  |  |
| 5090561 | ATP5EP2 | -2.4 |  |  |  |  |
| 7160059 | ATP6V0B | -2.4 |  |  |  |  |
| 3870630 | ATP6V1F | -2.4 |  |  |  |  |
| 5220438 | C20ORF52 | -2.4 |  |  |  |  |
| 4570091 | C3ORF60 | -2.4 |  |  |  |  |
| 2320129 | CSDA | -2.4 |  |  |  |  |
| 4150189 | CTSL1 | -2.4 |  |  |  |  |
| 1770520 | CYC1 | -2.4 |  |  |  |  |
| 1470259 | EXOC7 | -2.4 |  |  |  |  |
| 4480220 | FGFRL1 | -2.4 |  |  |  |  |
| 4200450 | G6PD | -2.4 |  |  |  |  |
| 3800035 | GLS2 | -2.4 |  |  |  |  |
| 1510468 | GRPEL1 | -2.4 |  |  |  |  |
| 1740576 | LMF2 | -2.4 |  |  |  |  |
| 2230626 | MRPL33 | -2.4 |  |  |  |  |
| 4060041 | MYST1 | -2.4 |  |  |  |  |
| 2360491 | NARF | -2.4 |  |  |  |  |
| 2320367 | NDUFA13 | -2.4 |  |  |  |  |
| 5080167 | NOTCH1 | -2.4 |  |  |  |  |
| 2710161 | SELK | -2.4 |  |  |  |  |
| 5050681 | TESC | -2.4 |  |  |  |  |
| 5670465 | ADM | -2.3 |  |  |  |  |
| 7040079 | AP1S1 | -2.3 |  |  |  |  |
| 5570152 | ATP1A1 | -2.3 |  |  |  |  |
| 770561 | C20ORF108 | -2.3 |  |  |  |  |
| 2370128 | DIRC2 | -2.3 |  |  |  |  |
| 5890605 | FAM109A | -2.3 |  |  |  |  |
| 3940133 | FAM46A | -2.3 |  |  |  |  |
| 2650019 | FAM53C | -2.3 |  |  |  |  |
| 6510487 | FAM96B | -2.3 |  |  |  |  |
| 6520128 | GPX4 | -2.3 |  |  |  |  |
| 2470367 | INPPL1 | -2.3 |  |  |  |  |
| 2710672 | KIAA2013 | -2.3 |  |  |  |  |
| 1230600 | LOC651064 | -2.3 |  |  |  |  |
| 3930326 | LOC728014 | -2.3 |  |  |  |  |
| 1850370 | MRPS12 | -2.3 |  |  |  |  |
| 5700370 | POLDIP3 | -2.3 |  |  |  |  |
| 1580093 | SCYL1 | -2.3 |  |  |  |  |
| 5550136 | SMAP2 | -2.3 |  |  |  |  |
| 5090068 | STX5 | -2.3 |  |  |  |  |
| 2760735 | SYNM | -2.3 |  |  |  |  |
| 4150309 | ZNHIT1 | -2.3 |  |  |  |  |
| 7210192 | ADA | -2.2 |  |  |  |  |
| 2480709 | ADCK5 | -2.2 |  |  |  |  |
| 5560102 | AGPAT2 | -2.2 |  |  |  |  |
| 620433 | C1ORF122 | -2.2 |  |  |  |  |
| 6380370 | CCND3 | -2.2 |  |  |  |  |
| 6330474 | CD151 | -2.2 |  |  |  |  |
| 610440 | CD81 | -2.2 |  |  |  |  |
| 5310170 | CORO1B | -2.2 |  |  |  |  |
| 7510356 | DGKQ | -2.2 |  |  |  |  |
| 5310634 | FASN | -2.2 |  |  |  |  |
| 7160504 | GPC1 | -2.2 |  |  |  |  |
| 2600382 | GPX4 | -2.2 |  |  |  |  |
| 670528 | GYG1 | -2.2 |  |  |  |  |
| 6620437 | HOXA5 | -2.2 |  |  |  |  |
| 4670343 | JAG2 | -2.2 |  |  |  |  |
| 1300369 | LOC255783 | -2.2 |  |  |  |  |
| 3940364 | LOC347376 | -2.2 |  |  |  |  |
| 5860608 | LOC400948 | -2.2 |  |  |  |  |
| 1780661 | MAN1B1 | -2.2 |  |  |  |  |
| 2230187 | MAPBPIP | -2.2 |  |  |  |  |
| 3460181 | MTCH1 | -2.2 |  |  |  |  |
| 3140193 | NELF | -2.2 |  |  |  |  |
| 4200692 | NEU1 | -2.2 |  |  |  |  |
| 1230754 | POR | -2.2 |  |  |  |  |
| 6250280 | PRDX1 | -2.2 |  |  |  |  |
| 990747 | PSCD2 | -2.2 |  |  |  |  |
| 2000445 | PSMB10 | -2.2 |  |  |  |  |
| 2070376 | RFNG | -2.2 |  |  |  |  |
| 1580309 | SERF2 | -2.2 |  |  |  |  |
| 4900431 | STUB1 | -2.2 |  |  |  |  |
| 2680064 | SYVN1 | -2.2 |  |  |  |  |
| 4860315 | TESK1 | -2.2 |  |  |  |  |
| 650519 | TMBIM6 | -2.2 |  |  |  |  |
| 4780612 | UNC93B1 | -2.2 |  |  |  |  |
| 4210041 | UQCRQ | -2.2 |  |  |  |  |
| 3520753 | ACAA1 | -2.1 |  |  |  |  |
| 6620403 | AGBL5 | -2.1 |  |  |  |  |
| 4230360 | AP3B2 | -2.1 |  |  |  |  |
| 5900445 | ARFGAP1 | -2.1 |  |  |  |  |
| 6130725 | ARHGEF18 | -2.1 |  |  |  |  |
| 1510088 | ATP1B3 | -2.1 |  |  |  |  |
| 4760112 | ATP5J2 | -2.1 |  |  |  |  |
| 6330377 | ATP6V0D1 | -2.1 |  |  |  |  |
| 3520092 | BAX | -2.1 |  |  |  |  |
| 6510608 | BTBD11 | -2.1 |  |  |  |  |
| 160242 | C13ORF15 | -2.1 |  |  |  |  |
| 2810082 | C20ORF111 | -2.1 |  |  |  |  |
| 3460278 | C22ORF13 | -2.1 |  |  |  |  |
| 70661 | CCDC72 | -2.1 |  |  |  |  |
| 3060128 | CHSY1 | -2.1 |  |  |  |  |
| 770168 | CLPTM1 | -2.1 |  |  |  |  |
| 5310707 | EI24 | -2.1 |  |  |  |  |
| 3440630 | ESPN | -2.1 |  |  |  |  |
| 7400747 | FAM89A | -2.1 |  |  |  |  |
| 4880673 | GADD45A | -2.1 |  |  |  |  |
| 5090671 | GDF15 | -2.1 |  |  |  |  |
| 4900170 | GPS2 | -2.1 |  |  |  |  |
| 1300678 | GRIN2C | -2.1 |  |  |  |  |
| 4280471 | GUK1 | -2.1 |  |  |  |  |
| 4900333 | HAGH | -2.1 |  |  |  |  |
| 7100136 | HES2 | -2.1 |  |  |  |  |
| 520184 | HPCAL1 | -2.1 |  |  |  |  |
| 6980224 | HS.128463 | -2.1 |  |  |  |  |
| 3170093 | KIAA0247 | -2.1 |  |  |  |  |
| 5820634 | LOC441150 | -2.1 |  |  |  |  |
| 4010433 | LOC650832 | -2.1 |  |  |  |  |
| 6450056 | MCOLN1 | -2.1 |  |  |  |  |
| 1170736 | MMP15 | -2.1 |  |  |  |  |
| 2750309 | MRPL53 | -2.1 |  |  |  |  |
| 1300671 | NCOA4 | -2.1 |  |  |  |  |
| 1510609 | NME1-NME2 | -2.1 |  |  |  |  |
| 160170 | PKM2 | -2.1 |  |  |  |  |
| 60653 | PRR7 | -2.1 |  |  |  |  |
| 1500600 | RAB37 | -2.1 |  |  |  |  |
| 1710189 | RHBDF1 | -2.1 |  |  |  |  |
| 2650524 | RPL34 | -2.1 |  |  |  |  |
| 5860148 | S100A13 | -2.1 |  |  |  |  |
| 3940692 | SBF1 | -2.1 |  |  |  |  |
| 7650017 | SERPINH1 | -2.1 |  |  |  |  |
| 5420367 | SOD1 | -2.1 |  |  |  |  |
| 6590463 | UBB | -2.1 |  |  |  |  |
| 4220180 | WDR68 | -2.1 |  |  |  |  |
| 7040139 | ZNF324 | -2.1 |  |  |  |  |
| 5810201 | ZNF593 | -2.1 |  |  |  |  |
| 5860215 | ABHD5 | -2.0 |  |  |  |  |
| 4810129 | BRMS1 | -2.0 |  |  |  |  |
| 1190220 | COX5B | -2.0 |  |  |  |  |
| 4250095 | COX6B1 | -2.0 |  |  |  |  |
| 3890017 | CTNNA1 | -2.0 |  |  |  |  |
| 240309 | CTSB | -2.0 |  |  |  |  |
| 430242 | DGUOK | -2.0 |  |  |  |  |
| 4120609 | DGUOK | -2.0 |  |  |  |  |
| 7040670 | DHRS3 | -2.0 |  |  |  |  |
| 7610131 | EPAS1 | -2.0 |  |  |  |  |
| 2600279 | GPS1 | -2.0 |  |  |  |  |
| 5820672 | HIC2 | -2.0 |  |  |  |  |
| 1770753 | HRAS | -2.0 |  |  |  |  |
| 5820255 | HSPC171 | -2.0 |  |  |  |  |
| 6040634 | IRX2 | -2.0 |  |  |  |  |
| 4480288 | ISG20L1 | -2.0 |  |  |  |  |
| 5860242 | LOC642755 | -2.0 |  |  |  |  |
| 1230220 | LOC729466 | -2.0 |  |  |  |  |
| 3420372 | RBM38 | -2.0 |  |  |  |  |
| 3890326 | SOD2 | -2.0 |  |  |  |  |
|  |  |  |  |  |  |  |
