# Supplementary material for: TFPI1 Mediates Resistance to Doxorubicin in Breast Cancer Cells by Inducing a Hypoxic-Like Response
Source: PLoS One. 2014 Jan 28;9(1):e84611. doi: 10.1371/journal.pone.0084611 (PMC3904823; doi:10.1371/journal.pone.0084611)
Supplement: Table S4 — Gene expression changes defining the acute and chronic phases of selection for DOX resistant MCF7 cells. Edges 1–7, 2–4, 3–5 and 4–6 refer to the numbering system described in Fig. S1. For example, Edge 1–7 refers to genes that are unchanged during acute exposure and down-regulated during chronic exposure. (DOCX) [file pone.0084611.s011.docx]

**Supplementary Table 4 Gene expression changes defining the acute and chronic phases of selection for DOX resistant MCF7 cells.** Edges 1-7, 2-4, 3-5 and 4-6 refer to the numbering system described in Suppl. Fig. 1. For example, Edge 1-7 refers to genes that are unchanged during acute exposure and down-regulated during chronic exposure.

| **Edge 1-7 (102)** | |  | **Edge 2-8 (53)** | |  | **Edge 3-5 (154)** | |  | **Edge 4-6 (118)** | |  |
| --- | --- | --- | --- | --- | --- | --- | --- | --- | --- | --- | --- |
| **(down in DOX)** | |  | **(up in DOX)** | |  | **(up in DOX48)** | |  | **(down in DOX48)** | |  |
|  |  |  |  |  |  |  |  |  |  |  |  |
| **Probe.ID** | **TargetID** | **FC** | **Probe.ID** | **TargetID** | **FC** | **Probe.ID** | **TargetID** | **FC** | **Probe.ID** | **TargetID** | **FC** |
| 2690047 | ARL6IP1 | -3.3 | 3990170 | IFI27 | 5.1 | 2140121 | BASP1 | 4.3 | 3890349 | HIST1H4C | -5.5 |
| 1510291 | PTTG1 | -3.2 | 3290630 | SERPINA5 | 3.7 | 2060291 | STOM | 3.8 | 6770438 | HIST1H1B | -4.2 |
| 5090156 | NBL1 | -3.0 | 1340039 | TFPI | 3.4 | 430465 | G6PD | 3.5 | 2070494 | PRC1 | -4.2 |
| 5560494 | RPS15 | -3.0 | 6180259 | HSPB8 | 3.3 | 4920767 | FTL | 3.2 | 2230619 | HIST1H4E | -4.1 |
| 5870678 | LOC441763 | -2.9 | 7150634 | APOD | 3.2 | 4260044 | SQSTM1 | 3.2 | 7650026 | MUC1 | -3.5 |
| 7160239 | FOSB | -2.8 | 6350189 | MGC4677 | 3.2 | 5810328 | FTL | 3.0 | 6650053 | MCM3 | -3.2 |
| 2030553 | H3F3A | -2.8 | 4070647 | ALDH3B2 | 3.1 | 4010064 | P4HA2 | 3.0 | 6280446 | LOC642989 | -3.0 |
| 5490603 | LOC401019 | -2.8 | 7400377 | CEACAM6 | 3.0 | 4590494 | YIF1A | 3.0 | 3830131 | TUBA1C | -3.0 |
| 4880477 | LOC643031 | -2.8 | 7570324 | ID3 | 3.0 | 4250291 | CHPF | 2.9 | 1940164 | LSM5 | -2.9 |
| 2640719 | RN7SK | -2.8 | 1010446 | C1QTNF6 | 2.8 | 1240750 | SNTB2 | 2.9 | 1470195 | MCM7 | -2.7 |
| 4850097 | RPLP2 | -2.8 | 4830520 | KRT80 | 2.8 | 2490754 | MAP1LC3B | 2.8 | 6200402 | MT1A | -2.7 |
| 1470086 | RPS19 | -2.8 | 1230523 | COMMD3 | 2.7 | 5670661 | MGC71993 | 2.8 | 450615 | MT2A | -2.7 |
| 3460477 | H3F3A | -2.6 | 5090215 | IFI6 | 2.7 | 6370538 | WBSCR22 | 2.8 | 1430735 | NET1 | -2.7 |
| 4730356 | C19ORF31 | -2.5 | 3310538 | CD36 | 2.6 | 3830653 | DNAJB2 | 2.7 | 6520424 | HIST1H3C | -2.6 |
| 6770025 | FAU | -2.5 | 7210632 | AKR1C3 | 2.4 | 5570279 | HIST1H1C | 2.7 | 2190674 | IGFBP5 | -2.6 |
| 7550470 | KIAA0101 | -2.5 | 4390372 | BNIPL | 2.4 | 620300 | LASP1 | 2.7 | 7200601 | MUC1 | -2.6 |
| 2370341 | LOC91561 | -2.5 | 3170519 | HARS2 | 2.4 | 4780040 | MRPL41 | 2.7 | 7380670 | MYB | -2.6 |
| 6900079 | PCNA | -2.5 | 1010246 | IFI6 | 2.4 | 270408 | P4HA2 | 2.7 | 3120341 | RAMP3 | -2.6 |
| 2640091 | GAPDH | -2.4 | 2940291 | QPRT | 2.4 | 2630056 | PRDX5 | 2.7 | 1170170 | STC2 | -2.6 |
| 1230164 | HS.534061 | -2.4 | 150474 | CA12 | 2.3 | 6200086 | PSAP | 2.7 | 2450156 | XBP1 | -2.6 |
| 5340154 | LOC643509 | -2.4 | 7330435 | CCDC6 | 2.3 | 1430477 | CTSL2 | 2.6 | 3060646 | C14ORF173 | -2.5 |
| 3140019 | LOC645317 | -2.4 | 6840368 | RDH11 | 2.3 | 3180053 | FAM129B | 2.6 | 5340338 | E2F2 | -2.5 |
| 2340452 | RN7SL1 | -2.4 | 4810435 | SCARB2 | 2.3 | 4490017 | GLTP | 2.6 | 150343 | HS.213061 | -2.5 |
| 4570482 | RPS10 | -2.4 | 3890398 | WBP2 | 2.3 | 6330132 | ISG20 | 2.6 | 870148 | MSH6 | -2.5 |
| 6180681 | AKR7A2 | -2.3 | 6130424 | AKR1A1 | 2.2 | 5670400 | PEPD | 2.6 | 6840056 | MYL6B | -2.5 |
| 3390022 | COX4I1 | -2.3 | 6480630 | ATP9A | 2.2 | 7650333 | PSAP | 2.6 | 4560600 | C3ORF57 | -2.4 |
| 870338 | EGR1 | -2.3 | 4830687 | BCAS3 | 2.2 | 1470427 | ALDH4A1 | 2.5 | 5860152 | CD44 | -2.4 |
| 2640048 | GAPDH | -2.3 | 6370612 | CHURC1 | 2.2 | 7160327 | ARPC2 | 2.5 | 3890475 | CENPN | -2.4 |
| 7650152 | GNB2L1 | -2.3 | 4890671 | DHRS2 | 2.2 | 1030458 | C19ORF10 | 2.5 | 7000634 | CSE1L | -2.4 |
| 4280307 | LAIR1 | -2.3 | 2510593 | HRASLS3 | 2.2 | 4490528 | CKAP4 | 2.5 | 4480180 | DEK | -2.4 |
| 4490594 | LOC399900 | -2.3 | 3890228 | MPZL2 | 2.2 | 5220398 | PH-4 | 2.5 | 4590356 | HNRNPD | -2.4 |
| 1070731 | LOC440567 | -2.3 | 6940255 | PLSCR3 | 2.2 | 1510564 | TMEM4 | 2.5 | 5690274 | MCM6 | -2.4 |
| 5870328 | LOC440589 | -2.3 | 2190184 | PRSS23 | 2.2 | 1770433 | YWHAG | 2.5 | 1940576 | RPS6KB1 | -2.4 |
| 6550139 | LOC441034 | -2.3 | 3460739 | RAB17 | 2.2 | 4780615 | ANXA2 | 2.4 | 1090239 | SF3B3 | -2.4 |
| 2760753 | MSH3 | -2.3 | 6250382 | ANAPC13 | 2.1 | 1010487 | BTG2 | 2.4 | 3710711 | STAG3L2 | -2.4 |
| 2230037 | NAG18 | -2.3 | 5090347 | CD24 | 2.1 | 1010735 | DDX24 | 2.4 | 290343 | TTC3 | -2.4 |
| 7100010 | NBL1 | -2.3 | 830735 | CENTA1 | 2.1 | 5570114 | GADD45G | 2.4 | 6580474 | TUBB | -2.4 |
| 7100239 | OAZ1 | -2.3 | 3170273 | FER1L3 | 2.1 | 520358 | IKBKG | 2.4 | 3850053 | ANP32B | -2.3 |
| 4860719 | ROCK2 | -2.3 | 5270619 | FGD3 | 2.1 | 1260360 | LOC729776 | 2.4 | 3450632 | C6ORF141 | -2.3 |
| 2680484 | RPL41 | -2.3 | 5260044 | GMFB | 2.1 | 3180600 | PNPO | 2.4 | 3360059 | CASP2 | -2.3 |
| 2070349 | SNRPB | -2.3 | 4560367 | NDUFA8 | 2.1 | 3310564 | PRDX5 | 2.4 | 5220767 | FLNB | -2.3 |
| 5900682 | SUMO3 | -2.3 | 6130138 | POLR3C | 2.1 | 70634 | RABAC1 | 2.4 | 7320750 | ILVBL | -2.3 |
| 430369 | TEX264 | -2.3 | 7560025 | PSMA1 | 2.1 | 4230554 | REXO2 | 2.4 | 4200068 | NUP62 | -2.3 |
| 1450273 | CLUAP1 | -2.2 | 50195 | RAB25 | 2.1 | 4250327 | RHOC | 2.4 | 7400358 | TINP1 | -2.3 |
| 610112 | FAM177A1 | -2.2 | 1260497 | RFTN1 | 2.1 | 3890408 | RRBP1 | 2.4 | 6280672 | TMEM49 | -2.3 |
| 7200608 | HSPB1 | -2.2 | 3120367 | SQLE | 2.1 | 1980360 | SDF4 | 2.4 | 5560246 | TPM1 | -2.3 |
| 5820360 | IMAA | -2.2 | 1990253 | TDG | 2.1 | 1230192 | SPNS1 | 2.4 | 5340187 | TUBA1A | -2.3 |
| 3400487 | LOC388474 | -2.2 | 7150196 | TMEM87A | 2.1 | 4220632 | ATP1B3 | 2.3 | 4210050 | WDR54 | -2.3 |
| 6860661 | MGC16703 | -2.2 | 1770546 | ATIC | 2.0 | 5090176 | C9ORF89 | 2.3 | 1070215 | CAV1 | -2.2 |
| 4490671 | MIF | -2.2 | 4280672 | KRT86 | 2.0 | 5290500 | CAPNS1 | 2.3 | 5810605 | CCNI | -2.2 |
| 5960224 | PTTG3 | -2.2 | 7100220 | XYLT2 | 2.0 | 4830682 | CCDC92 | 2.3 | 6550754 | EVL | -2.2 |
| 6550593 | RPL38 | -2.2 | 2900441 | ZBTB4 | 2.0 | 5900575 | CD276 | 2.3 | 2680450 | HIST1H1D | -2.2 |
| 3180470 | SDHA | -2.2 | 2490333 | ZNF467 | 2.0 | 1660541 | ESRRA | 2.3 | 6560672 | HNRPR | -2.2 |
| 4730086 | SHCBP1 | -2.2 |  |  |  | 4880685 | FBXO22 | 2.3 | 2120524 | IGFBP5 | -2.2 |
| 2470619 | AKT1 | -2.1 |  |  |  | 4850754 | GHITM | 2.3 | 4150128 | LOC340598 | -2.2 |
| 4860367 | ATRIP | -2.1 |  |  |  | 4670575 | HTATIP2 | 2.3 | 6180066 | NACA | -2.2 |
| 1990717 | C1ORF63 | -2.1 |  |  |  | 1820504 | NME1 | 2.3 | 3610064 | NACA | -2.2 |
| 6220180 | CABC1 | -2.1 |  |  |  | 580494 | PSMB4 | 2.3 | 2630161 | NOL11 | -2.2 |
| 5670093 | ECHS1 | -2.1 |  |  |  | 650369 | PSMD8 | 2.3 | 7150132 | PNN | -2.2 |
| 3460441 | EDF1 | -2.1 |  |  |  | 2970563 | RPRC1 | 2.3 | 6040347 | RAMP3 | -2.2 |
| 7160743 | F2R | -2.1 |  |  |  | 430411 | TOR3A | 2.3 | 3170184 | RPL36AL | -2.2 |
| 4490161 | GAPDH | -2.1 |  |  |  | 4610431 | ACTG2 | 2.2 | 4250445 | RPL4 | -2.2 |
| 2570156 | GGA1 | -2.1 |  |  |  | 2190341 | AP1S1 | 2.2 | 3780528 | RPS27A | -2.2 |
| 2710292 | H2AFZ | -2.1 |  |  |  | 6590201 | ATP6AP1 | 2.2 | 940075 | SPDEF | -2.2 |
| 5130253 | HGS | -2.1 |  |  |  | 4730343 | ATP6V0E1 | 2.2 | 1230044 | SSBP1 | -2.2 |
| 3120114 | HIST1H2AM | -2.1 |  |  |  | 5570324 | BMP1 | 2.2 | 4180050 | TIMELESS | -2.2 |
| 2340241 | IMPA2 | -2.1 |  |  |  | 940300 | C17ORF90 | 2.2 | 3840167 | TMEM14C | -2.2 |
| 1170609 | ITIH5 | -2.1 |  |  |  | 1690059 | CALML5 | 2.2 | 7150349 | TPD52L1 | -2.2 |
| 6620392 | LFNG | -2.1 |  |  |  | 6370369 | CD14 | 2.2 | 4780050 | UGDH | -2.2 |
| 770554 | LOC400963 | -2.1 |  |  |  | 4390327 | CLTA | 2.2 | 510341 | VEZF1 | -2.2 |
| 3830477 | LOC441246 | -2.1 |  |  |  | 6380128 | CLTA | 2.2 | 3420451 | C15ORF15 | -2.1 |
| 1940709 | LOC645895 | -2.1 |  |  |  | 1110095 | CLTB | 2.2 | 6560088 | C3ORF14 | -2.1 |
| 5810746 | MATN2 | -2.1 |  |  |  | 1740326 | CSGLCA-T | 2.2 | 7040184 | CCDC34 | -2.1 |
| 5910154 | NOL5A | -2.1 |  |  |  | 5360553 | ECH1 | 2.2 | 5360070 | CCNB2 | -2.1 |
| 2230296 | ORC6L | -2.1 |  |  |  | 3930189 | EIF4G1 | 2.2 | 130022 | CDCA5 | -2.1 |
| 2970521 | PGRMC1 | -2.1 |  |  |  | 1940228 | FAM58A | 2.2 | 1660528 | CKLF | -2.1 |
| 6370762 | POLR2F | -2.1 |  |  |  | 4560064 | GLB1 | 2.2 | 5130674 | CSE1L | -2.1 |
| 3990368 | PPP1CA | -2.1 |  |  |  | 2350066 | HLA-A | 2.2 | 4880360 | FBL | -2.1 |
| 630327 | RET | -2.1 |  |  |  | 5080692 | HLA-A29.1 | 2.2 | 5220022 | GFRA1 | -2.1 |
| 3130072 | RHOT2 | -2.1 |  |  |  | 5870521 | HLA-H | 2.2 | 3780056 | GGCT | -2.1 |
| 2630022 | RNPS1 | -2.1 |  |  |  | 5900438 | HOXC13 | 2.2 | 6020735 | GINS2 | -2.1 |
| 6400437 | RPL32 | -2.1 |  |  |  | 7210017 | NOMO2 | 2.2 | 3610286 | GLTSCR2 | -2.1 |
| 1170164 | RPL35 | -2.1 |  |  |  | 3840689 | PIGT | 2.2 | 6400270 | HPRT1 | -2.1 |
| 830066 | RPS24 | -2.1 |  |  |  | 4880551 | RBM42 | 2.2 | 5090754 | KIAA0101 | -2.1 |
| 2190537 | WDR74 | -2.1 |  |  |  | 5490608 | SELS | 2.2 | 4560056 | NONO | -2.1 |
| 3390477 | C17ORF79 | -2.0 |  |  |  | 6280168 | SERPINA3 | 2.2 | 6420424 | PAICS | -2.1 |
| 290132 | C20ORF117 | -2.0 |  |  |  | 2450639 | TSC22D1 | 2.2 | 4220707 | PMPCB | -2.1 |
| 540491 | COX7A2 | -2.0 |  |  |  | 20491 | UBE2F | 2.2 | 2760292 | PPP1CC | -2.1 |
| 3830605 | DNLZ | -2.0 |  |  |  | 1170647 | ACADVL | 2.1 | 780358 | RAB11A | -2.1 |
| 4540600 | FAM115A | -2.0 |  |  |  | 1170440 | AHCYL1 | 2.1 | 2030315 | RFC4 | -2.1 |
| 7330026 | FRAT2 | -2.0 |  |  |  | 4560110 | ARMET | 2.1 | 1450390 | RPL17 | -2.1 |
| 4040008 | LOC646195 | -2.0 |  |  |  | 4890487 | B2M | 2.1 | 7380689 | RPL22 | -2.1 |
| 6770286 | MCART1 | -2.0 |  |  |  | 240195 | B2M | 2.1 | 620754 | RPS5 | -2.1 |
| 7400025 | MTP18 | -2.0 |  |  |  | 20673 | BSG | 2.1 | 2850575 | RRM1 | -2.1 |
| 1090026 | PMPCA | -2.0 |  |  |  | 6290168 | C10ORF116 | 2.1 | 7160753 | SAE1 | -2.1 |
| 6650564 | RPS27 | -2.0 |  |  |  | 270133 | C1ORF128 | 2.1 | 4860692 | SHFM1 | -2.1 |
| 6380445 | SFRS5 | -2.0 |  |  |  | 6040156 | C6ORF52 | 2.1 | 4390546 | TINP1 | -2.1 |
| 2510411 | SLC44A4 | -2.0 |  |  |  | 1470397 | C8ORF33 | 2.1 | 6270600 | TMEM64 | -2.1 |
| 940435 | TRIM8 | -2.0 |  |  |  | 7550041 | CAPNS1 | 2.1 | 3990619 | TOP2A | -2.1 |
| 5820528 | TSEN34 | -2.0 |  |  |  | 6900195 | CNO | 2.1 | 3890255 | TOP2B | -2.1 |
| 630474 | ZP3 | -2.0 |  |  |  | 1230025 | CYB5R1 | 2.1 | 3610259 | TUBB | -2.1 |
|  |  |  |  |  |  | 6100768 | CYFIP2 | 2.1 | 3800647 | UGCG | -2.1 |
|  |  |  |  |  |  | 3120520 | GABARAPL2 | 2.1 | 5340129 | AKR1C2 | -2.0 |
|  |  |  |  |  |  | 670255 | GADD45A | 2.1 | 2850482 | ATP6V1B1 | -2.0 |
|  |  |  |  |  |  | 6110392 | GNS | 2.1 | 2680471 | ESD | -2.0 |
|  |  |  |  |  |  | 1940021 | GRN | 2.1 | 6580270 | LOC646723 | -2.0 |
|  |  |  |  |  |  | 2940301 | HPS6 | 2.1 | 1980246 | MYO5C | -2.0 |
|  |  |  |  |  |  | 6860593 | HS.568928 | 2.1 | 1850259 | NFIC | -2.0 |
|  |  |  |  |  |  | 4120086 | LAMC1 | 2.1 | 4920537 | POLA2 | -2.0 |
|  |  |  |  |  |  | 6280167 | LOC401115 | 2.1 | 6180537 | RBMX | -2.0 |
|  |  |  |  |  |  | 4760349 | LRP10 | 2.1 | 7040095 | RPL17 | -2.0 |
|  |  |  |  |  |  | 3120139 | NENF | 2.1 | 1440300 | SLC27A3 | -2.0 |
|  |  |  |  |  |  | 2030093 | PKM2 | 2.1 | 130519 | STAT2 | -2.0 |
|  |  |  |  |  |  | 1260440 | PRKAB1 | 2.1 | 6130193 | TMEM109 | -2.0 |
|  |  |  |  |  |  | 4880168 | PTTG1IP | 2.1 | 4880129 | TOMM7 | -2.0 |
|  |  |  |  |  |  | 2850100 | RNASET2 | 2.1 | 2650553 | TPD52L1 | -2.0 |
|  |  |  |  |  |  | 1340689 | RPN1 | 2.1 | 610519 | TPM1 | -2.0 |
|  |  |  |  |  |  | 3370112 | RPN2 | 2.1 |  |  |  |
|  |  |  |  |  |  | 5570678 | RPS27L | 2.1 |  |  |  |
|  |  |  |  |  |  | 3190112 | SERPINB1 | 2.1 |  |  |  |
|  |  |  |  |  |  | 7040315 | STX16 | 2.1 |  |  |  |
|  |  |  |  |  |  | 7570671 | UNKL | 2.1 |  |  |  |
|  |  |  |  |  |  | 4590154 | ZDHHC8 | 2.1 |  |  |  |
|  |  |  |  |  |  | 2970026 | ZNF622 | 2.1 |  |  |  |
|  |  |  |  |  |  | 5390347 | ZNF79 | 2.1 |  |  |  |
|  |  |  |  |  |  | 3520671 | AGPAT2 | 2.0 |  |  |  |
|  |  |  |  |  |  | 3370202 | ANXA2P1 | 2.0 |  |  |  |
|  |  |  |  |  |  | 2320110 | ATP6V0E1 | 2.0 |  |  |  |
|  |  |  |  |  |  | 6450162 | C9ORF169 | 2.0 |  |  |  |
|  |  |  |  |  |  | 7320435 | DPM3 | 2.0 |  |  |  |
|  |  |  |  |  |  | 2710068 | EIF3I | 2.0 |  |  |  |
|  |  |  |  |  |  | 6040376 | FAM127A | 2.0 |  |  |  |
|  |  |  |  |  |  | 1010068 | FKBP2 | 2.0 |  |  |  |
|  |  |  |  |  |  | 3400709 | HS.531457 | 2.0 |  |  |  |
|  |  |  |  |  |  | 2120544 | IMP3 | 2.0 |  |  |  |
|  |  |  |  |  |  | 4040564 | IRAK1 | 2.0 |  |  |  |
|  |  |  |  |  |  | 6270100 | LAMP1 | 2.0 |  |  |  |
|  |  |  |  |  |  | 3450427 | MED19 | 2.0 |  |  |  |
|  |  |  |  |  |  | 5310465 | MRPS12 | 2.0 |  |  |  |
|  |  |  |  |  |  | 6580608 | PEX16 | 2.0 |  |  |  |
|  |  |  |  |  |  | 2490259 | PINK1 | 2.0 |  |  |  |
|  |  |  |  |  |  | 6200017 | POLR2L | 2.0 |  |  |  |
|  |  |  |  |  |  | 2360682 | PSMB6 | 2.0 |  |  |  |
|  |  |  |  |  |  | 4390619 | RHOC | 2.0 |  |  |  |
|  |  |  |  |  |  | 5050437 | SCAND1 | 2.0 |  |  |  |
|  |  |  |  |  |  | 2650193 | SDC4 | 2.0 |  |  |  |
|  |  |  |  |  |  | 3890681 | SLC41A3 | 2.0 |  |  |  |
|  |  |  |  |  |  | 5390202 | TMED9 | 2.0 |  |  |  |
|  |  |  |  |  |  | 5310379 | TMEM115 | 2.0 |  |  |  |
|  |  |  |  |  |  | 1450082 | TRAPPC2L | 2.0 |  |  |  |
|  |  |  |  |  |  | 6350632 | TSC22D3 | 2.0 |  |  |  |
|  |  |  |  |  |  | 4760474 | TUBA4A | 2.0 |  |  |  |
|  |  |  |  |  |  | 70592 | VCL | 2.0 |  |  |  |
|  |  |  |  |  |  | 4570468 | ZNF263 | 2.0 |  |  |  |
