# Supplementary material for: TFPI1 Mediates Resistance to Doxorubicin in Breast Cancer Cells by Inducing a Hypoxic-Like Response
Source: PLoS One. 2014 Jan 28;9(1):e84611. doi: 10.1371/journal.pone.0084611 (PMC3904823; doi:10.1371/journal.pone.0084611)
Supplement: Table S5 — Up-regulated processes during acute DOX exposure. (DOCX) [file pone.0084611.s012.docx]

**Supplementary Table 5 Up-regulated processes during acute DOX exposure.**

| Category | Count | Genes |
| --- | --- | --- |
| metabolic process | 40 | ALDH4A1, RPS27L, FBXO22, PINK1, PEPD, SELS, RPN1, RPN2, ACADVL, BMP1, GADD45G, GADD45A, ECH1, POLR2L, SQSTM1, GNS, GABARAPL2, PIGT, ISG20, DNAJB2, AGPAT2, G6PD, IRAK1, UBE2F, RNASET2, REXO2, DPM3, DDX24, TSC22D1, PSAP, MRPL41, RRBP1, PSMB4, PSMB6, PSMD8, BTG2, PNPO, CSGLCA-T, GLB1, PKM2 |
| signal transduction | 31 | IRAK1, AGPAT2, RHOC, C9ORF89, SQSTM1, AGPAT2, YWHAG, C9ORF89, GADD45G, GADD45A, BSG, RPS27L, PINK1, CALML5, CD14, SELS, GRN, IKBKG/NEMO, PRKAB1, IRAK1, LASP1, PEPD, C19ORF10/IL27, YWHAG (14-3-3 family), ATP1B3, ESRRA, GHITM, AHCYL1, LRP10, ZNF622, IMP3 |
| protein and membrane transport | 25 | STX16, AP1S1, SQSTM1, CD14, SDF4 , GABARAPL2, HTATIP2, PEX16, SELS, PTTG1IP, GLTP, HTATIP2, ATP6V0E1, AP1S1, ATP6AP1, PSAP, SQSTM1, P4HA2, YIF1A, RABAC1, CLTA, CLTB, ARMET/MANF, TMED9, TRAPPC2L |
| stress response | 17 | PRDX5, G6PD, SELS, RPS27L, BTG2, GADD45G, GADD45A, ISG20, DNAJB2, IRAK1, SDF4, SERPINA3, PINK1, SQSTM1, EIF4G1, ANAX2, HLA-H |
| inflammatory and immune response | 16 | PRDX5, SERPINA3, SERPINB1, ISG20, C19ORF10/IL27, IRAK1, CD276, IKBKG/NEMO, TOR3A, CD14, HLA-A, HLA-A29.1, B2M, FKBP2, SDC4, TSC22D3 |
| potential oncogenes | 14 | CD276, PTTG1IP, EIF4G1, WBSCR22, LASP1, C19ORF10/IL27, ANAX2, RHOC, HOXC13, PKM2, ZDHHC8, IMP3, MED19, PINK1 |
| proteolysis | 11 | FBXO22, PSMB4, PSMB6, PSMD8, SQSTM1, SELS, RPN1, RPN2, UBE2F, CTSL2, PEPD |
| apoptosis | 9 | CYFIP2, PKM2, GADD45A, CD14, MRPL41, PSAP, FAM129B/MINERVA, GHITM, ZDHHC8 |
| Golgi apparatus | 9 | B2M, STX16, TMEM115, ARPC2, GABARAPL2, SDC4, PSAP, BSG, HLA-A |
| mitochondrial function | 9 | ACADVL, ECH1, MRPL41, MRPS12, PSAP, PRDX5, ALDH4A1, CYB5R1, PINK1 |
| transcriptional control | 8 | POLR2L, TSC22D1, CKAP4/p63, HOXC13, MED19, SCAND1, TSC22D3, ZNF263 |
| cytoskeletal organization | 7 | ARPC2, BASP1, MAP1LC3B, LASP1, FAM129B/MINERVA, ACTG2, CNO |
| coagulation and wound healing | 6 | VCL, FAM129B, SERPINA3, CD14, SDC4 |
| potential tumor suppressors | 6 | NME1, BTG2, HTATIP2/TIP30, GAD45A, BASP1, RNASET2 |
| positive regulation of cell proliferation | 6 | CAPNS1, NME1, LAMC1, CD276, TMEM115, BTG2, |
| ER membrane | 3 | RPN1, PEX16, DPM3, NOMO2 |
| peroxisome | 3 | PRDX5, ECH1, PEX16 |
| UPR and autophagy | 3 | SELS, DNAJB2, LAMP1 |
| RNA binding | 2 | RBM42, IMP3 |
| translation initiation | 2 | EIF3I, EIF4G1 |
| nuclear import | 2 | HTATIP2/TIP30, PTTG1IP |
| proton transport | 2 | ATP6V0E1, ATP6AP1 |
| histone modification | 1 | WBSCR22 |
| chromatin | 1 | HIST1H1C |
| other/unknown | 31 | STOM, SQSTM1, CHPF, SNTB2, MGC71993, PH-4, TMEM4, LOC729776, SPNS1, C9ORF89, CCDC92, RPRC1, C17ORF90, FAM58A, NOMO2, C10ORF116, C1ORF128, C6ORF52, C8ORF33, HS.568928, LOC401115, NENF, UNK, ZNF79L, ANXA2P1, C9ORF169, FAM127A, HS.531457, SLC41A3, TMEM115, TUBA4A |
