# Supplementary material for: TFPI1 Mediates Resistance to Doxorubicin in Breast Cancer Cells by Inducing a Hypoxic-Like Response
Source: PLoS One. 2014 Jan 28;9(1):e84611. doi: 10.1371/journal.pone.0084611 (PMC3904823; doi:10.1371/journal.pone.0084611)
Supplement: Table S6 — Up-regulated processes during chronic DOX exposure. (DOCX) [file pone.0084611.s013.docx]

**Supplementary Table 6 Up-regulated processes during chronic DOX exposure.**

| Category | Count | Genes |
| --- | --- | --- |
| metabolic process | 14 | ATIC, APOD, CD36, CD24, SQLE, TDG, ALDH3B2, QPRT, GMFB, DHRS2, XYLT2, AKR1C3, AKR1A1, NDUFA8 |
| potential oncogenes | 13 | BNIPL, CD24, CD36, TFPI, IFI6, CEACAM6, C1QTNF6, AKR1C3, CA12, CCDC6, BCAS3, PRSS23, RAB25 |
| signal transduction | 11 | CD36,CD24,GMFB,CEACAM6, C1QTNF6, WBP2, BCAS3, RAB17, CENTA1, FGD3, RAB25 |
| potential tumor suppressors | 8 | SERPINA5, HSPB8, ID3, WBP2, HRASLS3, RAB17, ANAPC13, ZBTB4 |
| blood coagulation and wound healing | 6 | CD36, TFPI, SERPINA5, CD24, PLSCR3, BCAS3 |
| mitochondrial function | 5 | IFI6, NDUFA8, IFI27, HARS2, DHRS2/HEP27 |
| immune response | 5 | IFI6, IFI27, DHRS2,CD24, MPZL2 |
| stress response | 5 | ALDH3B2, AKR1C1, AKR1C3, CCDC6, ANAPC13 |
| apoptosis | 4 | BNIPL, IFI27, HSPB8, PLSCR3 |
| protein transport | 3 | SCARB2, RAB17, RAB25 |
| retinoic acid biogenesis | 3 | RDH11, AKR1C1, AKR1C3 |
| regulation of transcription factor activity | 2 | ID3, ZBTB4 |
| negative regulation of caspase activity | 1 | IFI6 |
| proteolysis | 1 | PSMA1 |
| other/unknown | 11 | MGC4677, KRT80, COMMD3, ATP9A, CHURC1, FER1L3, POLR3C, RFTN1, TMEM87A, KRT86, ZNF467 |
