# Supplementary material for: TFPI1 Mediates Resistance to Doxorubicin in Breast Cancer Cells by Inducing a Hypoxic-Like Response
Source: PLoS One. 2014 Jan 28;9(1):e84611. doi: 10.1371/journal.pone.0084611 (PMC3904823; doi:10.1371/journal.pone.0084611)
Supplement: Table S7 — Down-regulated processes during acute DOX exposure. (DOCX) [file pone.0084611.s014.docx]

**Supplementary Table 7 Down-regulated processes during acute DOX exposure.**

| Category | Count | Genes |
| --- | --- | --- |
| metabolic processes | 34 | UGCG, UGDH, SF3B3, RPS27A, SAE1, RPL17, LSM5, RPS5, POLA2, RPL22, RPL4, NONO, SHFM1, HPRT1, PAICS, CASP2, NACA, HNRNPD, RBMX, AKR1C2, FBL, RFC4, TOP2A, MCM7, MCM3, MCM6, MSH6, ATP6V1B1, MT2A, SSBP1, TPM1, SPDEF, ESD, GGCT |
| chromatin binding | 19 | TOP2A, TOP2B, CDCA5, NUP62, HIST1H1D, HIST1H4C, HIST1H1B, HIST1H4E, HIST1H3C, PRC1, TIMELESS, RBMX, MCM3, MCM6, MCM7, DEK, VEZF1, CENPN, GINS2 |
| signal transduction | 19 | CKLF, GFRA1, TUBB, STC2, NUP6, NET1, RAMP3, FLNB, RPS6KB1, DEK, NUP62, IGFBP5, RFC4, TOP2A, STAT2, EVL, RAB11A, PPP1CC, CAV1 |
| RNA binding | 14 | XBP1, LSM1, LSM5, RPS5, RPL22, HNRPR, HNRNPD, RLP4, FBL, SF3B3, RBMX, NONO, NUP62, RPS27A |
| stress response | 14 | VEZF1, TUBB, RFC4, TOP2A, MCM7, TPM1, MSH6, TIMELESS, STC2, NUP62, MT1A, MT2A, CKLF, RRM1 |
| DNA binding | 10 | E2F2, TOP2A, MCM3, MCM7, MSH6, NFIC, RBMX, STAT2, MYB, SSBP1 |
| cell cycle progression | 10 | CSE1L, TIMELESS, TPD52L1, TOP2A, CDCA5, RPS27A, PRC1, MSH6, CCNB2, CAV1 |
| ribosome biogenesis | 9 | TINP1, NOL11, RPL36AL, RPL4, RPL27A, GLTSCR2, RPL17, RPL22, RPS5 |
| regulation of gene expression | 9 | RPS27A, TIMELESS, VEZF1, MYB, HNRNPD, DEK, NUP62, STAT2, NFIC |
| DNA replication | 7 | RFC4, MCM7, TOP2A, MCM3, MCM6, POLA2, GINS2 |
| translation elongation | 5 | RPL17, RPS27A, RPL4, RPS5, RPL22 |
| protein trafficking | 5 | RAB11A, NUP62, CSE1L, RAMP3, MYL6B |
| mitochondrial localization and function | 5 | TOMM7, GGCT, SSBP1, TMEM14C, PMPCB |
| apoptosis | 4 | CSE1L, NUP62, TOP2A, TUBB |
| protein ubiquitination | 3 | SAE1, RPS27A, TTC3 |
| cell adhesion | 2 | CD44, PNN |
| steroid metabolic process | 2 | ANP32B, AKR1C2 |
| cytoskeleton | 2 | TUBA1A, MYO5C |
| tumor suppressors | 2 | CAV1, GLTSCR2 |
| other | 17 | LOC642989, C14ORF173, HS.213061, STAG3L2, ILVBL, TMEM49, WDR54, CCNI, LOC340598, C15ORF15, C3ORF14, CCDC34, KIAA0101, SHFM1, TMEM64, LOC646723, TMEM109 |
