# Supplementary material for: TFPI1 Mediates Resistance to Doxorubicin in Breast Cancer Cells by Inducing a Hypoxic-Like Response
Source: PLoS One. 2014 Jan 28;9(1):e84611. doi: 10.1371/journal.pone.0084611 (PMC3904823; doi:10.1371/journal.pone.0084611)
Supplement: Table S8 — Down-regulated processes during chronic DOX exposure. (DOCX) [file pone.0084611.s015.docx]

**Supplementary Table 8 Down-regulated processes during chronic DOX exposure.**

| Category | Count | Genes |
| --- | --- | --- |
| nuclear function/compartment | 22 | RPS15, PCNA, POLR2F, SNRPB, ORC6L, ATRIP, AKT1, SUMO3, EDF1, RPS27, PGRMC1, PTTG1, RNPS1, EGR1, MSH3, EDF1, FOSB, HSPB1, F2R/PAR-1, H3F3A, H2AFZ, HIST1H2AM |
| ribosome biogenesis | 16 | FAU, RPS19, RPS15, RPS10, RPS27, RPS24, RPL35, RPLP2, RPL38, RPL41, RPL32, WDR74, PGRMC1, POLR2F, HSPB1, GNB2L1/RACK1 |
| RNA binding | 11 | RPLP2, FAU, RPS19, RPS15, RPS10, RPS27, RPS24, RPL41, RPL35, RPL38, SNRPB |
| signal transduction | 10 | F2R/PAR-1, AKT1, ATRIP, PCNA, GNB2L1/RACK1, SHCBP1, LFNG, PPP1CA, ROCK2, RET |
| mitochondrial biogenesis | 8 | SDHA, PMPCA, COX4I1, COX7A2, RHOT2, ECHS1, CABC1, DNLZ |
| stress response | 7 | PCNA, HSPB1, ATRIP, AKT1, MSH3, F2R/PAR-1, KIAA0101/p15PAF |
| apoptosis | 5 | RHOT2, AKT1, HSPB1, MIF, ARL6IP1 |
| protein trafficking | 4 | AKT1, GGA1, F2R/PAR-1, HGS |
| cell cycle | 4 | ROCK2, AKT1, PTTG1/securin, PTTG3/meiotic securin |
| regulation of gene expression | 3 | SFRS5, RNPS1, POLR2F |
| regulation of blood coagulation | 3 | F2R/PAR-1, EGR1, MATN2 |
| tumor suppressor | 3 | NBL1, CLUAP1, ITIH5 |
| sugar metabolism | 3 | AKT1, GAPDH, IMPA2 |
| protein ubiquitination | 2 | PTTG1/mitotic securin, PTTG3/meiotic securin |
| steroid metabolic process | 1 | AKR7A2 |
| Other | 30 | PMPCA, LAIR, OAZ1, LOC441763, LOC401019, LOC643031, RN7SK, C19ORF31, LOC91561, HS.534061, OC643509, LOC645317, RN7SL, LOC399900, LOC440567, LOC440589, LOC441034, NAG18, TEX264, FAM177A1, IMAA, LOC388474, MGC16703, C1ORF63, LOC400963, LOC441246, LOC645895, NOL5A, C17ORF79, C20ORF117 |
