# Supplementary material for: TFPI1 Mediates Resistance to Doxorubicin in Breast Cancer Cells by Inducing a Hypoxic-Like Response
Source: PLoS One. 2014 Jan 28;9(1):e84611. doi: 10.1371/journal.pone.0084611 (PMC3904823; doi:10.1371/journal.pone.0084611)
Supplement: Table S9 — A comparison of up- and down-regulated metabolic processes associated with selection of DOX resistant MCF7 cells. The genes that make up each metabolic process are listed in the right column. (DOCX) [file pone.0084611.s016.docx]

**Supplementary Table 9 A comparison of up- and down-regulated metabolic processes associated with selection of DOX resistant MCF7 cells.** The genes that make up each metabolic process are listed in the right column.

| Category | Count | Genes |
| --- | --- | --- |
| **Up-regulated** |  |  |
| protein metabolic process | 21 | RPS27L, FBXO22, DNAJB2, PINK1, IRAK1, UBE2F, PEPD, SELS, DPM3, RPN1, RPN2, MRPL41, BMP1, SQSTM1, RRBP1, PSMB4, PSMB6, PSMD8, PIGT, PNPO, CSGLCA-T |
| catabolic process | 15 | ISG20, ALDH4A1, PEPD, RNASET2, SELS, FBXO22, ACADVL, BMP1, ECH1, SQSTM1, GNS, PSMB4, PSMB6, GABARAPL2, PSMD8 |
| DNA metabolic process | 13 | ISG20, RPS27L, RNASET2, REXO2, DDX24, TSC22D1, GADD45G, GADD45A, POLR2L, BTG2, CTSL2, WBSCR22, HIST1H1C |
| carbohydrate metabolic process | 10 | G6PD, DPM3, RPN1, RPN2, GNS, GLB1, PKM2, XYLT2, TDG, AKR1A1 |
| lipid biosynthetic process | 9 | AGPAT2, G6PD, DPM3, SQLE, AKR1C3, ALDH3B2, DHRS2, APOD, CD36 |
| ubiquitin-dependent protein catabolic process | 9 | FBXO22, PSMB4, PSMB6, PSMD8, SQSTM1, SELS, UBE2F, RPN1, RPN2 |
| protein catabolic process | 8 | BMP1, PEPD, SQSTM1, SELS, FBXO22, PSMB4, PSMB6, PSMD8 |
| cellular alcohol metabolic process | 4 | TDG, ALDH3B2, SQLE, AKR1A1 |
| carboxylic acid catabolic process | 3 | ACADVL, ECH1, ALDH4A1 |
| phospholipid biosynthetic process | 2 | AGPAT2, DPM3 |
| steroid metabolic process | 2 | DHRS2, SQLE |
|  |  |  |
| **Down-regulated** |  |  |
| protein metabolic process | 29 | CASP2, SAE1, NACA, SHFM1, TINP1, NOL11, RPL36AL, RPL4, RPL27A, GLTSCR2, RPL17, RPL22, RPS5, FAU, RPS19, RPS15, RPS10, RPS27, RPS24, RPL35, RPLP2, RPL38, RPL41, RPL32, WDR74, PGRMC1, POLR2F, HSPB1, GNB2L1/RACK1 |
| nucleic acid metabolic process | 16 | UGDH, SF3B3, LSM5, POLA2, HNRNPD, RBMX, FBL, NONO, RFC4, TOP2A, HPRT1, PAICS, MCM7, MCM3, MCM6, MSH6 |
| RNA metabolic process | 11 | NONO, LSM5, HNRNPD, RBMX, SF3B3, FBL, VEZF1, MYB, DEK, STAT2, NFIC |
| steroid metabolic process | 1 | AKR1C2 |
| glucose metabolic process | 1 | UGCG |
